# Targeted proteomics identifies circulating biomarkers associated with active COVID-19 and post-COVID-19

**DOI:** 10.3389/fimmu.2022.1027122

**Published:** 2022-11-03

**Authors:** Martijn Zoodsma, Aline H. de Nooijer, Inge Grondman, Manoj Kumar Gupta, Agnes Bonifacius, Valerie A. C. M. Koeken, Emma Kooistra, Gizem Kilic, Ozlem Bulut, Nina Gödecke, Nico Janssen, Matthijs Kox, Jorge Domínguez-Andrés, Adriaan J. van Gammeren, Anton A. M. Ermens, Andre J. A. M. van der Ven, Peter Pickkers, Rainer Blasczyk, Georg M. N. Behrens, Frank L. van de Veerdonk, Leo A. B. Joosten, Cheng-Jian Xu, Britta Eiz-Vesper, Mihai G. Netea, Yang Li

**Affiliations:** ^1^ Centre for Individualised Infection Medicine (CiiM), a joint venture between the Helmholtz Centre for Infection Research (HZI) and Hannover Medical School (MHH), Hannover, Germany; ^2^ TWINCORE, a joint venture between the Helmholtz Centre for Infection Research (HZI) and the Hannover Medical School (MHH), Hannover, Germany; ^3^ Department of Internal Medicine and Radboudumc Center for Infectious Diseases, Radboud University Medical Center, Nijmegen, Netherlands; ^4^ Institute of Transfusion Medicine and Transplant Engineering, Hannover Medical School, Hannover, Germany; ^5^ Department of Intensive Care Medicine and Radboudumc Center for Infectious Diseases, Radboud University Medical Center, Nijmegen, Netherlands; ^6^ Department of Clinical Chemistry and Hematology, Amphia Hospital, Breda, Netherlands; ^7^ Department of Rheumatology and Immunology, Hannover Medical School, Hannover, Germany; ^8^ German Center for Infection Research (DZIF), partner site Hannover-Braunschweig, Hannover, Germany; ^9^ Department of Medical Genetics, Iuliu Haţieganu University of Medicine and Pharmacy, Cluj-Napoca, Romania; ^10^ Department for Genomics and Immunoregulation, Life and Medical Sciences Institute (LIMES), University of Bonn, Bonn, Germany

**Keywords:** SARS-CoV-2, inflammation, targeted proteomics, biomarker, post-COVID-19

## Abstract

The ongoing Coronavirus Disease 2019 (COVID-19) pandemic is caused by the highly infectious Severe Acute Respiratory Syndrome Coronavirus-2 (SARS-CoV-2). There is an urgent need for biomarkers that will help in better stratification of patients and contribute to personalized treatments. We performed targeted proteomics using the Olink platform and systematically investigated protein concentrations in 350 hospitalized COVID-19 patients, 186 post-COVID-19 individuals, and 61 healthy individuals from 3 independent cohorts. Results revealed a signature of acute SARS-CoV-2 infection, which is represented by inflammatory biomarkers, chemokines and complement-related factors. Furthermore, the circulating proteome is still significantly affected in post-COVID-19 samples several weeks after infection. Post-COVID-19 individuals are characterized by upregulation of mediators of the tumor necrosis (TNF)-α signaling pathways and proteins related to transforming growth factor (TGF)-ß. In addition, the circulating proteome is able to differentiate between patients with different COVID-19 disease severities, and is associated with the time after infection. These results provide important insights into changes induced by SARS-CoV-2 infection at the proteomic level by integrating several cohorts to obtain a large disease spectrum, including variation in disease severity and time after infection. These findings could guide the development of host-directed therapy in COVID-19.

## 1 Introduction

Severe acute respiratory syndrome coronavirus-2 (SARS-CoV-2) is the causative agent of coronavirus disease-2019 (COVID-19), which was classified as a pandemic in March 2020 (1). While COVID-19 is often asymptomatic or mild in healthy individuals, elderly individuals or those with pre-existing co-morbidities are at risk of severe disease. Earlier outbreaks of related coronaviruses (severe acute respiratory syndrome [SARS] and Middle East respiratory syndrome [MERS]) indicated that the long-term effects of infection are considerable (2). In line with these findings, recent studies also demonstrated that individuals recovering from COVID-19 might experience fatigue or muscle weakness (63%), sleep difficulties (26%), and anxiety/depression (23%) as long as six months after symptom onset (3). Moreover, pulmonary functional and radiological abnormalities were reported four months after infection (4). Strikingly, long-lasting symptoms are also reported in non-critical COVID-19 patients several months after symptom onset (5, 6). These findings raise questions regarding the long-term consequences of infection at the molecular level.

Taken together, there is an urgent need for understanding the mechanisms underlying COVID-19 and establishing biomarkers for patient stratification, to be able to identify patients who will progress to severe disease or take more time to recover. Targeted proteomics has proven successful in identifying key mediators of disease, also in COVID-19 (7–12). However, most of these studies are confined to smaller sample sizes, and few studies include individuals after the initial phase of the disease.

In the present study, we used targeted proteomics to systematically investigate circulating protein concentrations from hospitalized patients (both ICU and non-ICU, *n* = 96 and 254, longitudinally sampled), post-COVID-19 individuals (*n* = 186), and uninfected healthy individuals (*n* = 61) recruited from three independent cohorts of Western European background. We show that the circulating proteome differs between COVID-19 patients with severe or critical disease. Comparison of hospitalized COVID-19 patients to healthy individuals reveals dysregulation of inflammatory and complement-related factors. Interestingly, our study shows that post-COVID-19 individuals are characterized by dysregulation of mediators of the tumor necrosis factor-α (TNFα) pathway and consistent upregulation of matrix metalloproteinases (MMPs). These findings suggest ongoing inflammation in post-COVID-19. Altogether, these results provide insights in the circulating biomarkers associated with disease severity and different phases of disease in COVID-19. Our findings will contribute to the development of host-directed therapy in COVID-19 and improve current healthcare strategies.

## 2 Materials and methods

### 2.1 Subject details and sample collection

**Figure 1:**
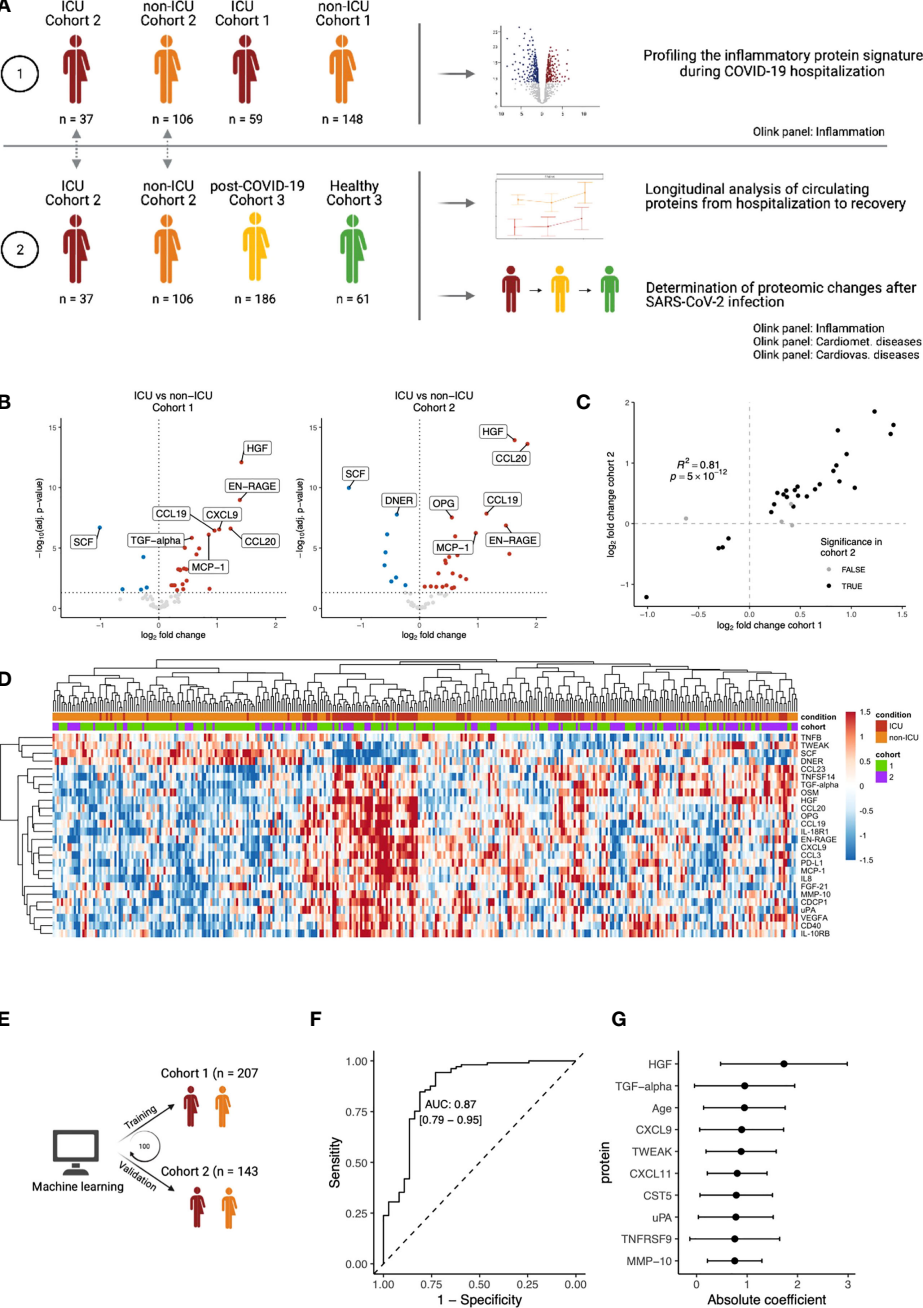
Inflammatory proteomic profile of hospitalized COVID-19 patients. **(A)** Schematic overview of the study design. **(B)** Differential abundance analysis on inflammation-related of ICU vs. non-ICU patients in cohorts 1 and 2, separately. Upregulation (red) indicates higher protein concentrations in ICU patients, whereas downregulation (blue) indicates downregulation in ICU patients. Proteins that are not significantly different are shown in grey. The vertical dotted line indicates log-fold change 0. The horizontal dotted line indicates significance at the adjusted *p*-value level. For both cohorts, only the first time-point per patient was considered. Since we focus on the inflammation-related proteins in this analysis, only proteins belonging to the Olink Inflammation panel are depicted. The complete differential abundance results are included in **Table S2**. **(C)** Replication of the significantly regulated proteins from cohort 1 in cohort 2. The log-fold changes per protein are strongly correlated (Pearson’s r^2^: 0.81). Overall, 62 proteins overlapped between the cohorts. 30 proteins were significant in cohort 1, of which 26 were significantly replicated in cohort 2 (FDR<0.05 and the same direction of regulation, shown in black). **(D)** Heatmap of the 26 replicated proteins (FDR < 0.05 & same direction of regulation in cohorts 1 and 2). Each column represents a patient, whereas rows represent proteins. The column annotations indicate the condition of each patient and the cohort to which they belong. Samples were clustered based on correlation. **(E)** Schematic overview of the training process for an elastic net linear regression model to discriminate between COVID-19 disease severity. The larger cohort 1 was used as a training cohort, and the smaller cohort 2 for validation. 100 independent training iterations were performed and combined to avoid potential bias. **(F)** Receiver-operating characteristic curve produced by one random iteration of the classification model on the independent validation data from cohort 2. The AUC was 0.86 (sensitivity: 0.76, specificity: 0.86). **(G)** Mean coefficients per protein were calculated over 100 independent training runs for the model. The top ten proteins with the highest absolute mean coefficients are shown. Dots indicate the mean absolute mean coefficient per protein, and error bars indicate standard deviation.

Three cohorts from Breda, Nijmegen, and Hannover were used to collect plasma samples from hospitalized COVID-19 patients, post-COVID-19 individuals and healthy controls to perform thorough proteomic profiling. Hospitalized COVID-19 patients were stratified based on treatment in an intensive care unit (ICU; critical COVID-19) or clinical ward (non-ICU; severe COVID-19) (**Figure 1A**). All applicable study protocols were approved by the local ethics board before initiation of the study, and all patients or their legal representatives gave informed consent for participation in this study. Information about age, sex, COVID-19 specific information, and blood cell counts for all cohorts are provided in **Table 1**.

**Table 1:** Quality assessment of individual cohort study.

	Cohort 1: Breda		Cohort 2: Nijmegen		Cohort 3: Hannover		P
	ICU	non-ICU	ICU	non-ICU	Post-COVID-19	Healthy	
**General**							
N	59	148	37	106	186	61	
Age	67 ± 9	70 ± 12	64 ± 12	63 ± 14	43 ± 12	46 ± 14	<2x10^-16^
Gender (M / F)	45 / 14	88 / 60	28 / 9	69 / 37	99 / 87	36 / 25	0.002
BMI	28 ± 4	28 ± 5	27 ± 4	27 ± 4^a^	–	–	0.36
							
**COVID-19**							
PCR-proven COVID-19 (n (%))	59 (100%)	148 (100%)	34 (92%)	100 (93%)	186 (100%)	–	
Anti-SARS-CoV-2 Spike 1 antibody	–	–	–	–	3.23 ± 2.32	0.40 ± 0.20	
Anti-SARS-CoV-2 Nucleocapsid protein antibody	–	–	–	–	2.32 ± 1.45	0.18 ± 0.16	
Days since symptom offset (days)	–	–	–	–	37 ± 11	–	
Hospitalization after COVID-19 symptom onset (days)	–	–	8 ± 3.4	7 ± 11.6	–	–	
Sampling post-hospitalization (days)							
Timepoint 1	3 ± 3.4	3 ± 2.9	5 ± 3.4	3 ± 3.7	–	–	
Timepoint 2	6 ± 2.6	5 ± 2.3	7 ± 3.5	5 ± 4.1	–	–	
Timepoint 3	8 ± 3.4	7 ± 2.4	9 ± 3.6	7 ± 4.2	–	–	
							
Cell counts (* 10^9^ / L)							
Lymphocytes	–	–	0.8 ± 0.5^b^	1.2 ± 2.3^b^	2.1 ± 0.8^c^	1.3 ± 0.4^d^	
Monocytes	–	–	0.5 ± 0.5^b^	0.6 ± 0.9^b^	–	–	
Neutrophils	–	–	6.4 ± 3.8^b^	5.8 ± 3.5^b^	–	–	
Eosinophils	–	–	0.005 ± 0.01^b^	0.01 ± 0.04^b^	–	–	
Basophils	–	–	0.02 ± 0.02^b^	0.01 ± 0.02^b^	–	–	
T cells	–	–	–	–	1.5 ± 0.6^c^	0.9 ± 0.3^d^	
B cells	–	–	–	–	0.17 ± 0.14^c^	0.14 ± 0.06^d^	
Plasmablasts	–	–	–	–	0.002 ± 0.004^c^	0.001 ± 0.001^d^	

Continuous variables are represented as mean ± standard deviation. For the cell count information, superscript denoted values are obtained from (a) 101 / 105 (b) 106 / 143 (c) 156 / 186 (d) 35 / 61 individuals. Cohort 2 (Nijmegen) was described in Janssen et al (2021) (9). Cohort 3 (Hannover) was partially described in Bonifacius et al (2021) (13). P-values were calculated by anova or chi-square test for continuous variables or categorical variables, respectively.

#### 2.1.1 Cohort 1: Breda

In this cohort, 59 COVID-19 ICU (mean age: 67 ± 9 years) and 148 COVID-19 non-ICU (mean age: 70 ± 12 years) patients were recruited in March and April 2020 (**Table S1**). Patients were admitted based on a PCR-proven SARS-CoV-2 infection. Ethylenediaminetetraacetic acid (EDTA) blood was collected during routine venipuncture for laboratory testing and stored at 4°C until further processing in the laboratory. After centrifugation for 10 min (3800rm) at room temperature, plasma was stored at -80°C until further processing.

#### 2.1.2 Cohort 2: Nijmegen

In this cohort, 38 COVID-19 ICU (mean age: 64 ± 12 years) and 106 COVID-19 non-ICU (mean age: 63 ± 14 years) patients were recruited between March and May 2020 (**Table S2**). This cohort was presented and described in a recent publication (9). It is important to note that this study partly repeats the analyses presented by Janssen and colleagues. The novelty of our analyses of cohort 2 lies in the analysis of the longitudinal sampling, which were not considered by Janssen and colleagues. Patients were admitted to the Radboud University Medical Center and diagnosed with COVID-19 based on a PCR-proven SARS-CoV-2 infection or suspected infection based on clinical features and observations of computed tomography (CT) scans. On average, ICU patients were admitted 8 days (sd: 3 days), and non-ICU patients were admitted on average 7 days (sd: 12) days after the first COVID-19 symptoms. EDTA blood was collected three times per week (ICU) or every 48 hours (non-ICU) during routine venipuncture for laboratory testing. After centrifugation for 10 minutes at 3800 rpm (2954 g) at room temperature, plasma was stored at -80°C until further processing

#### 2.1.3 Cohort 3: Hannover

In this cohort, 187 post-COVID-19 individuals (mean age: 43 ± 12) and 61 healthy individuals (mean age: 46 ± 14) were recruited between April and October 2020 in the Institute of Transfusion Medicine and Transplant Engineering (ITT), Hannover Medical School (**Table S3**). This cohort was partially presented in a recent publication (13). Healthy individuals were considered SARS-CoV-2 unexposed since they were (i) recruited early during the pandemic, (ii) regular blood and platelet donors and hence carefully and frequently evaluated with respect to the health status and (iii) SARS-CoV-2 seronegativity. All post-COVID-19 individuals had PCR-proven SARS-CoV-2 infection (**Figure S1**), and were sampled on average 37 days (ranging from 13 – 75 days) after symptom clearance (information available for 176/187 patients). Plasma was collected after centrifugation of whole blood samples and stored at -20°C until further processing. SARS-CoV-2 serology test was performed by ELISA: anti- SARS-CoV-2 Spike protein 1 (S1) IgG and anti-SARS-CoV nucleocapsid protein (NPC), IgG according to the manufacturer’s instructions (Euroimmun, Lübeck, Germany). Antibody amounts are expressed as IgG ratio (optical density divided by calibrator).

### 2.2 Initial demographic analysis

Initial demographic analysis was performed to investigate the differences in age, sex, BMI and blood cell counts between the conditions. In **Table 1**, ANOVA was used to calculate the statistical difference in age and BMI, while the chi-square test was used to test the difference in males and females for each condition. In the text, one-sided Wilcoxon ranked-sum tests were used to compare age and blood cell counts of specific conditions to healthy individuals. P-values<0.05 were considered significant.

### 2.3 Proteomic analysis

The multiplex proximity extension assay (PEA) from Olink Proteomics AB (Uppsala, Sweden) was used to quantify circulating proteins in plasma (14). The PEA is designed for high-throughput protein measurement in liquid samples. In this assay, oligonucleotide-labelled antibodies (“probes”) bind the protein of interest. The close proximity of two antibodies triggers the linking of the probes, thereby limiting cross-reactivity. Upon linking, the probe sequence hybridizes and is extended by DNA polymerase. The resulting sequence acts as a unique identifier for the protein and is quantified by a real-time polymerase chain reaction. Proteins are expressed as normalized protein expression (NPX) values, a relative value on a log2 scale.

In cohort 1, the Inflammation panel (v.3022) was measured. In cohort 2, three unique Olink panels, namely Inflammation v.3022, Cardiometabolic v.3603, and Cardiovascular II v.5006 were measured. In cohort 3, four unique panels, namely Inflammation v.3022, Cardiometabolic v.3603, Cardiovascular II v.5006, and Neurology v.8013 were measured. These targeted proteomic panels were chosen based on their relevance in the context of COVID-19. For instance, the connection between inflammatory and cardiometabolic/cardiovascular processes have been well-described for COVID-19 (15, 16). The neurological panel was chosen in cohort 3 because headaches and taste & smell dysfunctions are prevalent neurological symptoms during COVID-19 (17).

Quality control of the raw data was performed by Olink (Incubation controls, extension controls, and detection controls; detailed description available at: https://www.olink.com/question/are-internal-controls-included-in-the-assay-and-if-so-what-are-they). We removed protein assays where the target protein was detected in less than 80% of the samples and samples that were reported as unreliable by Olink per plate (Cohort 1: 7 samples, cohort 2: 25 samples, cohort 3: 14 samples removed). Later, we manually inspected principal component analysis (PCA) plots and removed apparent outliers (Cohort 1: 0 samples, cohort 2: 1 sample, cohort 3: 1 sample removed). Overall, duplicated proteins across the selected panels were consistent between replicates (**Figure S2**).

Subsequently, we merged three Olink cohorts together by using bridging samples that were included in all cohorts. All normalization procedures were performed in accordance with instructions from Olink (full description available at: www.olink.com/content/uploads/2018/05/Data-normalization-and-standardization_v1.0.pdf). In summary, the median protein abundance per protein is calculated for the bridging samples. Any difference in median protein abundance between two cohorts is considered technical variation and is corrected in one of the two cohorts. We excluded protein assays where the target protein was detected in less than 80% of the samples in either cohort. After merging the cohorts, bridging samples were removed from further analyses

### 2.4 Differential protein abundance analysis

The R (v.4.0.3) package *limma* (v3.46.0 (18)) was used to perform differential protein abundance analysis between (i) COVID-19 ICU vs. COVID-19 non-ICU, (ii) COVID-19 ICU vs. healthy (iii) COVID-19 non-ICU vs. healthy, and (iv) post-COVID-19 vs. healthy using a linear model with age and sex as covariates. *limma* uses an empirical Bayes method to moderate the standard errors of the estimated log-fold changes. Benjamini-Hochberg *post-hoc* correction was used to control the false discovery rate. Adjusted p-values <0.05 were considered statistically significant.

### 2.5 Replication of the post-COVID proteome signature in single-cell RNA sequencing

Post-COVID-19 single-cell RNA sequencing data published by Yoshida and colleagues (19) was downloaded from the COVID-19 cell atlas (https://www.covid19cellatlas.org). The downloaded objects were converted to Seurat objects. Differential expression was performed using Seurat’s Wilcoxon-test, comparing each disease severity (mild, moderate, severe) to healthy individuals. In total, 56 proteins from the post-COVID-19 proteome signature were confidently matched to their respective genes. Proteins were considered replicated when the corresponding gene was nominally significant and directionally concordant with the proteome.

### 2.6 Predictive modelling

Two separate linear regression models were used to predict (i) COVID-19 disease severity and (ii) the time after SARS-CoV-2 infection. The disease severity prediction model’s hyperparameters were selected based on training on all protein concentrations from cohort 1 using a five-fold repeated cross-validation strategy, and the final model’s performance was evaluated in cohort 2. Hyperparameters were selected based on the receiver-operator characteristics (ROC) metric. Both cohorts used only the first timepoint available for each patient. For the time after infection prediction, missing values were imputed using K-nearest neighbor imputation. The model’s hyperparameters were selected on 70% of the post-COVID-19 samples in cohort 3 using a five-fold repeated cross-validation strategy. The final model’s performance was evaluated on the remaining 30%. Hyperparameters were selected based on the “RMSE” metric. The R packages *glmnet* (20) and *caret* (21) were used to fit the models using standard parameters. Hyperparameters alpha and lambda were constrained to ranges [0.01, 0.09] and [0, 1], respectively. This entire process was repeated 100 times to calculate the mean and standard deviations of predictors included in the model between independent runs. Proteins were ranked based on their average coefficient over 100 independent training iterations. Subsequently, receiver-operating characteristics (ROC) analysis was performed to investigate whether the identified proteins had significant diagnostic effectiveness for separating critical patients (ICU) from severe patients (non-ICU). The R package *pROC* was used to generate the ROC curve and calculate the area under the curve (22). To predict the time after infection the correlation between reported and predicted values was taken as a measure of performance.

### 2.7 Longitudinal analysis

Repeated-measure ANOVA was used to investigate protein dynamics over time. We identified a subset of patients within cohort 2 that were consistently sampled over time (n = 78) and used median imputation to even the number of longitudinal measurements per patient (15 measurements imputed, 6.4% of the data). We only considered proteins that were significantly differentially abundant between COVID-19 ICU or COVID-19 non-ICU patients compared to healthy individuals. We tested these proteins for three effects: (i) Time effect, where the protein differs over time regardless of conditions. (ii) Condition effect, where the protein differs between COVID-19 ICU and COVID-19 non-ICU patients. (iii) The interaction between time and condition, where the protein differs over time but in different ways between the disease groups. We fitted an ANOVA model (*aov* function in base R, type I ANOVA) using the formula:

*value ~ condition + time + condition×time + Error(sampleID)*,

where value is the protein abundance level expressed in NPX. Condition is a categorical variable indicating whether patients are admitted to the ICU or not. Time represents the sampling time. We started from cohort 2 where more proteins were measured and replicated the overlapping proteins in cohort 3 (n = 45 proteins in total).

### 2.8 Co-expression networks and protein-protein interactions

The R package *igraph* (23) was employed to calculate Pearson’s correlation between all protein concentrations in both healthy, post-COVID-19 and hospitalized COVID-19 patients, separately. This in turn was used to generate co-expression networks for each condition. Subsequently, the generated networks were imported to Cytoscape 3 for visual alterations (24). We identified hub genes by ranking the proteins based on their betweenness centrality as calculated by Cytoscape. Betweenness centrality is a widely employed measure that captures each node’s role in allowing information to pass from one part of the network to the other and helps us to identify nodes of importance in the network.

## 3 Results

### 3.1 Baseline characteristics of the studied populations

We enrolled hospitalized COVID-19 patients, post-COVID-19 symptom-free individuals and healthy controls from two independent cohorts collected at the Amphia Hospital Breda and Hannover Medical School (n=207 hospitalized COVID-19 patients, n=186 post-COVID-19 patients and n=61 healthy individuals). Furthermore, we included and re-analyzed a recently published cohort of hospitalized COVID-19 patients (n=143) (9) (**Figure 1A**; **Table S4**). Hospitalized patients were included based on polymerase chain reaction (PCR)-proven SARS-CoV-2 infection or clinical features and computed tomography (CT) scan observations, and were admitted either to intensive care units (ICU) or clinical wards (non-ICU). Blood was taken every two to three days from the moment of hospitalization, with a mean of three longitudinal samples per patient. In this study, we include the first three time-points after hospitalization, thus representing the peak of the disease directly after hospitalization. Post-COVID-19 individuals were enrolled on average 37 days (standard deviation: 11 days) after clearance of mild COVID-19 symptoms. The time after infection is defined as the time between symptom offset and sampling, and thus indicates the time after self-reported recovery per individual. Healthy individuals were all SARS-CoV-2 immunoglobulin-G (IgG) seronegative at the time of enrollment. Overall, including the first three longitudinal samples of hospitalized patients, our cohorts include 1002 samples.

In our study, ICU (mean age: 65 ± 10 years) and non-ICU (mean age: 67 ± 13 years) patients were older compared to healthy individuals (mean age: 46 ± 14 years). Blood lymphocyte counts were significantly lower in ICU (0.8×10^9^/L, p=7×10^-5^) and non-ICU patients (1.2×10^9^/L, p=2×10^-9^), whereas post-COVID-19 individuals (2.1×10^9^/L, p=7×10^-13^) displayed higher lymphocyte counts compared to healthy individuals. A detailed description of demographics and clinical characteristics can be found in **Table 1**.

### 3.2 Inflammatory proteomic profiling of hospitalized COVID-19 patients

To characterize the immune response during COVID-19, we used the proximity extension assay (PEA)-based immunoassay (Olink platform) to investigate the inflammatory proteomic signature in both ICU and non-ICU patients from Breda (n=207, cohort 1) and Nijmegen (n=143, cohort 2). Within each cohort, differential abundance analysis on 62 circulating inflammation-related proteins revealed substantial differences between the ICU and non-ICU patients (**Figure 1B**; **Table S5**). To assess the reproducibility of our findings, we used the larger cohort 1 as the discovery cohort and validated the findings in the smaller cohort 2. The log-fold changes per protein were strongly correlated between the two cohorts (r^2 =^ 0.88, **Figure 1C**). Out of 30 significant proteins in cohort 1, 26 proteins replicated significantly in cohort 2 (False discovery rate (FDR) <0.05 and the same direction of regulation). In both cohorts, ICU patients were marked by significantly increased circulating concentrations of hepatocyte growth factor (HGF, adj. p=7×10^-13^ & 1×10^-14^ in cohort 1 and 2, respectively), CCL20 (adj. p=2×10^-7^ & 2×10^-14^), and MMP10 (adj. p=3×10^-5^ & 1×10^-3^). In contrast, ICU patients in both cohorts had significantly lower concentrations of stem cell factor (SCF, adj. p=2×10^-7^ & 1×10^-10^), Delta and Notch-like epidermal growth factor-related receptor (DNER, adj. p=5×10^-5^ & 1×10^-8^), and TNF-related weak inducer of apoptosis (TWEAK, adj. p=2×10^-2^ & 1×10^-2^). Protein concentrations of the 26 replicated proteins were visualized in a heatmap (**Figure 1D**). For both ICU and non-ICU patients, we observed substantial heterogeneity in terms of the circulating protein concentrations. Unsupervised clustering on the protein concentrations revealed that samples did not cluster respective to their cohort, but rather by condition. However, the separation between the conditions was gradual: some ICU patients clustered together with non-ICU patients, and vice-versa. This suggests a gradual change in the inflammatory circulating proteome from severe COVID-19 (non-ICU patients) to critical COVID-19 (ICU patients).

Subsequently, we investigated the capacity of the inflammatory signature to discriminate between ICU and non-ICU patients across cohorts. A linear regression model with elastic net regularization was trained on the protein concentrations of all 62 proteins in cohort 1, and validated in cohort 2 (**Figure 1E**). The model classified 98% of patients correctly in the training cohort and 83% in the validation cohort (**Figure S3**). This demonstrates the capacity of inflammatory proteins to discriminate disease severity in COVID-19 (**Figure 1F**, area under the curve (AUC): 0.87, sensitivity: 0.76, specificity: 0.86). Proteins with the largest power (highest average coefficient over 100 independent iterations) to discriminate between ICU and non-ICU patients are HGF, transforming growth factor (TGF)-α and CXCL9 (**Figure 1G**). Interestingly, we also identified age as a critical determinant of disease severity, in line with previous research that identifies age as a risk factor for COVID-19. Seven out of the ten proteins with the highest average coefficients were also significantly different between ICU and non-ICU patients in both cohorts in our previous analysis. Altogether, these results provide a comprehensive overview of the inflammatory proteomic signature in hospitalized COVID-19 patients.

### 3.3 Identification of biomarkers for COVID-19 disease severity and post-COVID-19

**Figure 2:**
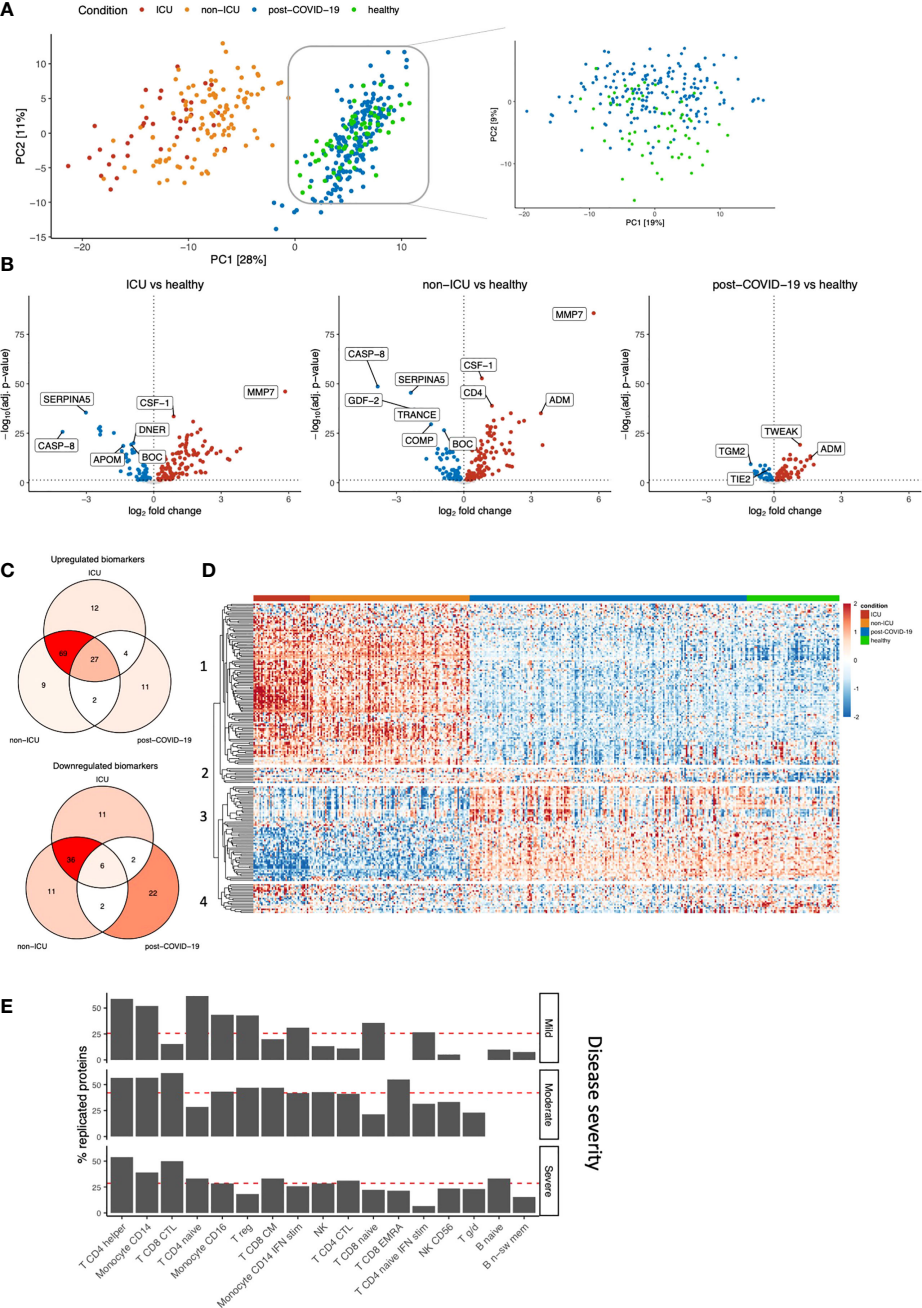
Identification of biomarkers for COVID-19 severity and post-COVID-19. **(A)** PCA on the protein concentrations of COVID-19 hospitalized patients, post-COVID-19 individuals, and healthy individuals (left). We performed PCA-based dimensionality reduction on cohort 3 separately to highlight the differences between healthy and post-COVID-19 individuals (right). **(B)** Significantly different proteins in ICU, non-ICU and post-COVID-19 individuals compared to healthy individuals. Upregulation (red) indicates higher protein concentrations compared to healthy individuals, whereas downregulation (blue) indicates lower proteins concentrations compared to healthy individuals. Proteins that do not significantly differ are shown in grey. The vertical dotted line indicates log-fold change 0. The horizontal dotted line demonstrates significance at the adjusted p-value level. For ICU and non-ICU patients, only the first time-point per patient was considered. **(C)** Shared and exclusive significantly abundant proteins between the conditions, compared to healthy individuals. Venn diagrams are colored with respect to the number of proteins. **(D)** Heatmap of the significantly differentially abundant proteins. Each row represents a protein that is significantly different in any of the conditions: ICU, non-ICU or post-COVID-19 individuals compared to healthy. Columns represent patients, and the column annotations indicate the condition of each patient. For ICU and non-ICU patients, only the first time point was considered. **(E)** Barplot showing replication of the post-COVID-19 proteome signature in publicly available single-cell RNA sequencing data. We replicated the post-COVID-19 signature in post-COVID-19 patients who experienced three different COVID-19 severities: Mild, Moderate or Severe. Proteins were considered replicated when the corresponding gene was nominally significant compared to healthy individuals and directionally concordant with the proteome. Red lines indicate the average replication rate calculated over these cell types. Only the most abundant cell types are shown.

Next, we compared hospitalized patients and post-COVID-19 individuals to healthy controls to investigate proteomic dynamics in the early phase of SARS-CoV-2 infection and after infection, respectively. We examined up to 220 circulating proteins related to inflammation, cardiovascular & cardiometabolic disease in 390 individuals. Principal component analysis (PCA)-based dimensionality reduction revealed a clear separation between hospitalized patients and post-COVID-19/healthy individuals based on the circulating proteome. ICU and non-ICU patients were moderately separated, consistent with our previous findings. Re-embedding of the post-COVID-19 and healthy individuals showed gradual separation between the two groups (**Figure 2A**). These results indicate that the proteome of hospitalized patients is remarkably different from post-COVID-19 or healthy individuals, while the differences between post-COVID-19 and healthy individuals are more subtle.

Subsequently, differential abundance analysis was performed to compare each disease class to healthy individuals separately (**Figure 2B**; **Table S5**). The analysis revealed that both ICU and non-ICU patients are characterized by differences in inflammatory cytokines and chemokines (among others TNFα, CXCL10, CXCL11 and CCL19), complement-related factors (SERPINA5, F2, CR2, C1QTNF1, C2), as well as consistent upregulation of MMP7. Albeit to a lesser extent, the circulating proteome from post-COVID-19 individuals showed clear differences compared to healthy individuals, revealing the differences between these groups at the proteome level. Using a recently published study that compared critical and severe COVID-19 patients to healthy controls (8), 49% and 11% of differentially abundant proteins can be replicated for ICU and non-ICU patients (FDR<0.05 in both studies and the same direction of change), respectively, while 77% (ICU) and 54% (non-ICU) of our significant proteins showed the same direction of abundance change.

We then assessed the overlap between significantly up- and downregulated proteins per group compared to healthy individuals (**Figure 2C**; **Table S6**). In total, 196 proteins were significantly regulated in one or more conditions. We observed a shared disease signature in ICU and non-ICU patients: 97% (191/196) of proteins were significantly regulated in at least one of these conditions, of which 72% (138/191) was shared. 64% (123/191) of the significant proteins in hospitalized patients are no longer significantly different in post-COVID-19 individuals. In post-COVID-19 individuals, 37% (73/196) of proteins are significantly different compared to healthy individuals. Among these, 41% (30/73) are specific to post-COVID-19. 17% (33/196) of proteins are significantly dysregulated among all disease classes in the same direction, suggesting these proteins play an important role during the disease, while potentially influencing immune responses after the infection phase as well. Highly significant proteins in this group are related to the proteolytic balance in the extracellular matrix (ECM) (MMP7 and MMP1) and TNFα signaling pathways (TNFα, TWEAK). Subsequently, the protein concentrations of the differentially regulated proteins were visualized to show their dynamics across conditions (**Figure 2D**). The proteomes of ICU and non-ICU patients followed largely the same patterns, showing that the difference was mainly at the quantitative level. These findings are consistent with earlier claims that the difference between critical and severe COVID-19 patients is a magnitude difference rather than a mechanistic difference (9). Finally, unsupervised clustering on the protein concentrations (**Figure 2D**; **Table S7**) revealed three unique proteomic dynamics:

(i) Clusters 1 and 3 contain 167 proteins with significant regulation in hospitalized patients (97%, 163/167). The concentrations of most of these proteins return to normal levels in post-COVID-19 individuals, although the concentrations of 35% (58/167) of them remain significantly different compared to healthy individuals. These two clusters contain mainly inflammatory markers, such as IL-18, IFN-γ, TNFα and chemokines, including IL-8, CXCL1, CXCL5, CXCL6, CXCL9, CCL3, and CCL17, and represent the acute infection proteomic signature.
(ii) Cluster 2 includes 10 proteins with consistent upregulation in both disease as well as post-COVID-19 compared to healthy individuals. The most significant proteins in this cluster were vascular endothelial growth factor D (VEGFD), MMP1, TWEAK, and proteins related to metabolism, e.g., glutaminyl-peptide cyclotransferase (QPCT) and peptidyl-glycine alpha-amidating monooxygenase (PAM). These proteins are consistently upregulated in both the acute infection phase and after the infection, and thus represent the proteomic signature that remains perturbed over a longer period of time after SARS-CoV-2 infection (together with the 35% proteins from clusters 1-3 that remain upregulated). From here on, we will refer to these proteins as the post-COVID-19 signature.
(iii) Cluster 4 consists of 19 proteins that are mostly significantly downregulated, although not all are significant in all conditions. Proteins in this cluster are related to angiopoietin signaling: angiopoietin like 3 (ANGPTL3) and tyrosine kinase with immunoglobulin-like and EGF-like domains 1 (TIE1) and lymphatic vessel endothelial hyaluronic acid receptor 1 (LYVE1).

Finally, we replicated our proteome post-COVID-19 signature in publicly available single-cell RNA sequencing data (19) (**Figure 2E**, **Figure S4**). From our proteome signature, 69% of the proteins were replicated (nominal P<0.05 & directionally concordant) in at least one celltype, indicating good consistency between targeted proteomics and the single-cell transcriptome. Overall, we found that our post-COVID-19 proteome signature was best replicated in diverse CD4+ T cell populations, as well as CD8+ T cells and monocytes. These results underline the robustness of our proteome signature and highlight the cellular populations that contribute to this proteomic signature.

In summary, these findings reveal the influence of SARS-CoV-2 infection at the proteome level for both hospitalized and post-COVID-19 individuals. Strikingly, post-COVID-19 individuals remained markedly different from healthy individuals at the proteomic level more than one month after SARS-CoV-2 infection.

### 3.4 Circulating protein concentrations are associated to time after SARS-CoV-2 infection

**Figure 3:**
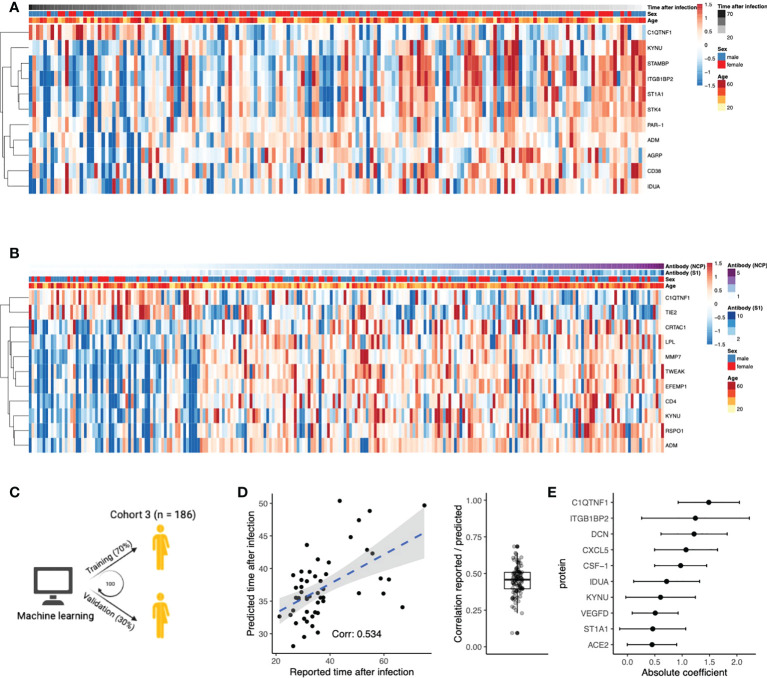
Circulating proteins predict COVID-19 recovery time. **(A)** Proteins significantly correlated to time the after infection in post-COVID-19 individuals. The time after infection is defined as the time between symptom offset and sampling and thus indicates the time after self-reported recovery per individual. Each row represents a protein, and each column represents a sample. Individuals are ordered by increasing time after recovery. **(B)** Proteins significantly correlated to either SARS-CoV-2 Spike 1 (S1) antibody or nucleocapsid protein (NCP) antibody concentrations in post-COVID-19 individuals. Each row represents a protein, and each column represents a sample. Samples are ordered in increasing NCP antibody concentrations. **(C)** Schematic overview of the training process for an elastic net linear regression model to predict time after SARS-CoV-2 infection. 70% of the data from post-COVID-19 individuals was used for training, and 30% for validation. Hundred independent training iterations were performed and combined to avoid potential bias. **(D)** Scatterplot showing the relation between the reported time after SARS-CoV-2 infection in days (x-axis) versus the predicted time after SARS-CoV-2 infection in days (y-axis) of one random run of the predictive model. The blue line represents a linear regression model fitted to the data, with standard error depicted in gray. Correlation between these values is a good indicator of the quality of the prediction. The boxplot shows the correlation between the predicted and reported values in the validation data over 100 independent runs. Boxplot center line: median, box limits: 1^st^ and 3^rd^ quartiles. Whiskers: 1,5 x interquartile range. **(E)** Mean coefficients per protein were calculated over 100 independent training runs for the model. The top ten proteins with the highest absolute mean coefficients are shown. Dots indicate the mean absolute mean coefficient per protein, and error bars indicate standard deviation.

Next, we aimed to identify proteins that play an important role after the infection phase of the disease. We investigated whether protein concentrations correlated with time after infection and anti-SARS-CoV-2 antibody titers in post-COVID-19 individuals, thereby indicating the importance of these proteins in the post-infection phase. In total, eleven proteins were significantly correlated to the time after infection (**Figure 3A**, Pearson’s r^2^, FDR<0.05). For instance, positive correlation indicates protein concentrations increase over time after infection. Subsequently, we assessed the correlations of the proteome with anti-SARS-CoV-2 antibody concentrations (**Figure 3B**). Concentrations of the spike (S1) and nucleocapsid (NCP) antibodies were strongly correlated to each other (Pearson’s r^2 =^ 0.73, p<2×10^-16^). Eleven unique proteins were significantly correlated to either S1 or NCP antibody concentrations (Pearson’s r^2^, FDR<0.05), highlighting the proteins that could be associated to protective immunity against COVID-19.

Furthermore, we intended to strengthen this line of evidence by investigating whether the proteome could predict time after infection. Linear regression models with elastic net regularization were trained on the concentrations of all 304 proteins within cohort 3 (**Figure 3C**). Using cross-validation, the model achieved an average correlation of r=0.45 between the reported and predicted time after infection (**Figure 3D**). The average coefficient in the prediction model was calculated over 100 independent iterations. The top ten most influential proteins, selected based on their coefficient, are shown in **Figure 3E**. We identified VEGFD, C1q tumor necrosis factor-related protein 1 (C1QTNF1), and angiotensin-converting enzyme 2 (ACE2) as proteins of interest because of their previous associations with COVID-19. Recently, VEGFD was proposed as a biomarker for COVID-19 due to growing evidence for its implications in acute respiratory distress syndrome (ARDS) and acute lung injury (25–27). The anticoagulant C1QTNF1 regulates blood coagulation, a well-described complication in COVID-19 patients (28). ACE2 is crucial in SARS-CoV-2 infection as this protein facilitates entry to the cell for the virus (29). Interestingly, predictor variables age and sex were consistently excluded from the model during variable selection, suggesting that their contribution in predicting the time after infection is less significant than plasma proteins. This is consistent with the fact that we do not find significantly different self-reported recovery times between males and females in cohort 3 (Wilcoxon rank-sum test, p=0.9). In summary, we identified proteins that are associated to the time after infection through correlation analyses and supervised machine learning approaches.

### 3.5 Dynamic changes in circulating proteins can be used to monitor disease severity in COVID-19 and serve as potential therapeutic targets

**Figure 4:**
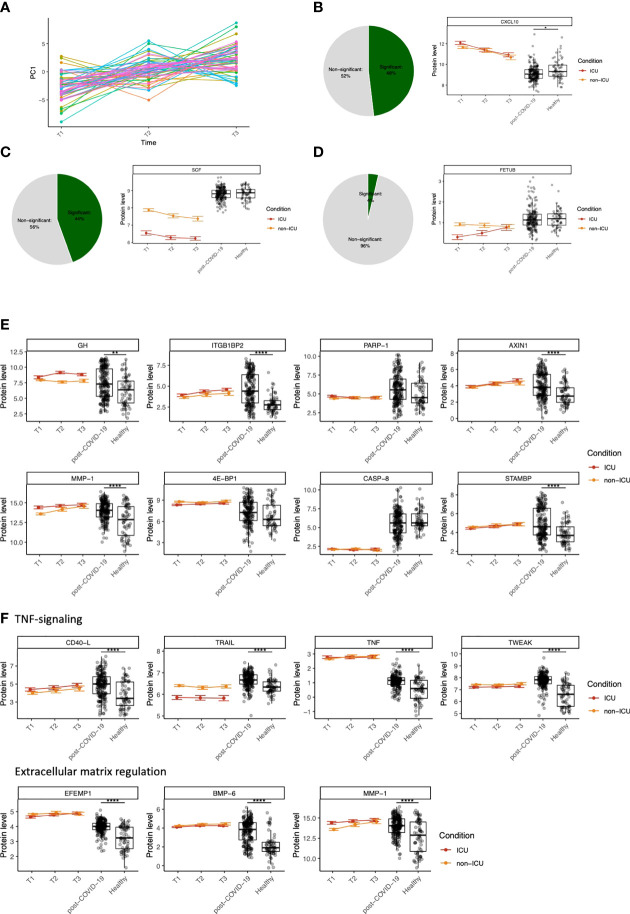
Dynamic changes in circulating proteins can be used to monitor disease severity in COVID-19 and serve as potential therapeutic targets. **(A)** ANOVA-PCA on the protein concentrations of the first three time-points after COVID-19 hospitalization in cohort 2. The time-points are taken at 48-hour intervals, and thus represent the first six days after hospitalization. 61 patients were selected who were consistently samples three times. The mean protein abundance per patient was subtracted from the measurements for these patients. The first principal component of this data was plotted against the time. **(B)** Representative example of a protein (CXCL10) that significantly differs over time during COVID-19 hospitalization, both in ICU and non-ICU patients. The pie chart indicates the proportion of proteins found to be significant for this effect. Red and orange colors indicate protein concentrations in COVID-19 ICU patients and non-ICU patients, respectively. **(C)** Representative example of a protein (stem cell factor, SCF) that significantly differs between conditions (ICU and non-ICU). The pie chart indicates the proportion of proteins found to be significant for this effect. Red and orange colors indicate protein concentrations in COVID-19 ICU patients and non-ICU patients, respectively. **(D)** Representative example of a protein (fetuin B precursor, FETUB) that significantly differs over time *and* between conditions. The pie chart indicates the proportion of proteins found to be significant for this effect. Red and orange colors indicate protein concentrations in COVID-19 ICU patients and non-ICU patients, respectively. **(E)** Longitudinal dynamics of heterogeneous proteins in post-COVID-19 individuals. The variance of normalized protein abundance values was calculated and the top eight were selected for visualization. Five out of eight proteins were significantly upregulated in post-COVID-19 individuals as indicated by the significance stars. **(F)** Longitudinal dynamics of proteins related to TNF-signaling and regulation of the extracellular matrix. These proteins were selected based on their highly significant differential abundance in post-COVID-19 individuals compared to healthy individuals. In all figures, significance is shown when the protein is significantly differentially expressed in post-COVID-19 individuals compared to healthy individuals: *adj. p < 0.05, **adj. p < 0.01, ***adj. p < 0.001, ****adj. p. < 0.0001. Boxplot center line: median, box limits: 1^st^ and 3^rd^ quartiles. Whiskers: 1,5 x interquartile range. For the hospitalized patients, error bars indicate standard deviation.

Intending to present a comprehensive overview of longitudinal proteomic dynamics from the point of hospitalization to post infection, we leveraged the longitudinal sampling of hospitalized patients and compared the circulating protein concentrations in hospitalized patients with post-COVID-19 and healthy individuals, separately. First, we applied ANOVA-PCA (30) to visualize the patterns of plasma proteins during the first three time-points after hospitalization (**Figure 4A**). Samples were collected every two to three days, and thus represent the first week of hospitalization. Overall, the main dynamic observed is upregulation along the first principal component, corresponding to an upregulation of circulating protein concentrations.

Next, we aimed to quantify the longitudinal protein dynamics during hospitalization. We identified three types of dynamic protein patterns: proteins that differ (i) over time in both ICU and non-ICU patients (**Figure 4B**), (ii) between conditions but not over time, (**Figure 4C**), and (iii) over time but in different ways between ICU vs. non-ICU patients (**Figure 4D**). Starting from cohort 2, we systematically tested for these effects in 189 proteins that were significantly different between ICU or non-ICU patients compared to healthy controls. Out of 69 overlapping proteins within cohort 1, 46 proteins were validated (FDR<0.05). Subsequently, we combined the longitudinal dynamics with the protein concentrations in post-COVID-19 and healthy individuals to generate an overview of the dynamic changes per protein over time and condition.

With the availability of good coverage of post-infection sampling times in cohort 3, we further hypothesized that highly variable proteins during the post-COVID-19 phase are likely to be influenced by infection. To assess this, we visualized eight proteins with the highest variance in post-COVID-19 samples (**Figure 4E**). Five out of eight proteins were regulated in a significantly different manner in post-COVID-19 individuals compared to healthy individuals. Six out of eight proteins were significant in the longitudinal analysis for at least one effect (time [ITGB1BP2, AXIN1, MMP1, STAMBP] or condition [GH, ITGB1BP2, 4E-BP1]), suggesting their importance both during hospitalization and after infection. Furthermore, two proteins (STAM binding protein (STAMBP) and ITGB1BP2) were significantly correlated to the time after SARS-CoV-2 infection within cohort 3 (FDR<0.05). None of the eight proteins were significantly correlated with antibody concentrations in post-COVID-19 individuals.

Finally, we highlighted two groups of proteins related to TNF-signaling and regulation of the ECM, respectively, because of their importance in COVID-19 (31, 32) (**Figure 4F**). We identified CD40-Ligand (CD40-L), TNFα, TNF-related apoptosis-inducing ligand (TRAIL), and TWEAK as proteins involved in TNF-signaling. All proteins were significantly upregulated in both ICU and non-ICU patients, except TRAIL, which was significantly downregulated in ICU, but not in non-ICU patients, showing a consistent condition effect over time. Moreover, we identified proteins related to the degradation and regulation of the ECM, namely EFEMP1, bone morphogenetic protein 6 (BMP-6), and MMP1. All proteins associated with these two processes were highly significantly regulated between post-COVID-19 and healthy individuals.

### 3.6 Modules of co-expressed proteins change over conditions

**Figure 5:**
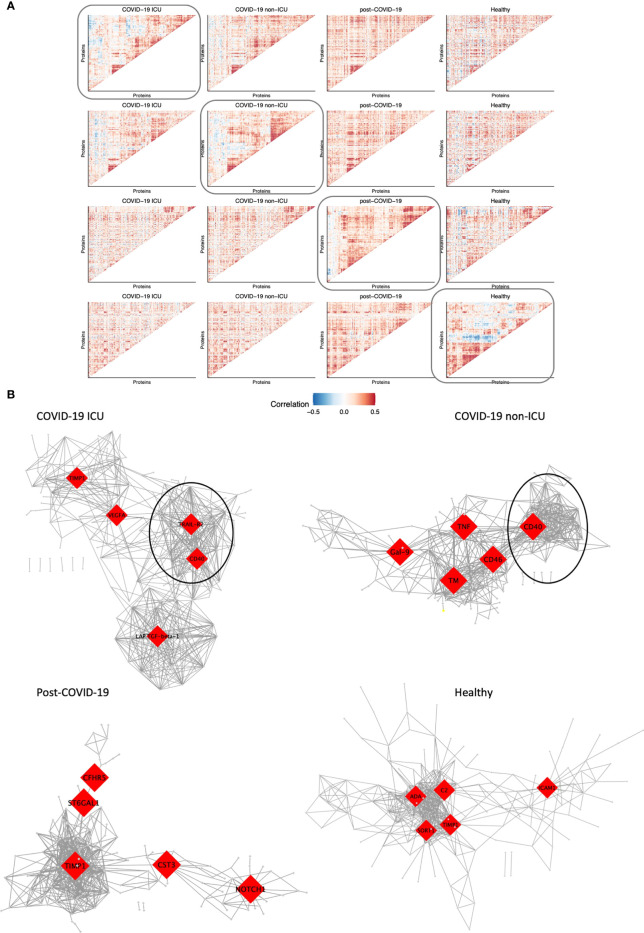
Modules of co-expressed proteins change over conditions. **(A)** Visualization of modules of co-expressed proteins per condition. Protein-protein correlations were calculated for each condition separately. Hierarchical clustering was performed on the matrix of correlation values for one condition as indicated by the box. The same protein order was kept for all heatmaps in the same row to visualize how modules change over condition relative to one condition. This was performed systematically for all four conditions: COVID-19 ICU, non-ICU, post-COVID-19, and healthy individuals. **(B)** Co-expression network of each condition. The top five hub genes for each network are highlighted in red. Only the top 2.5% highest correlated connections are shown, corresponding to 576 edges.

We aimed to assess whether modules of co-expressed proteins differed between hospitalized patients, post-COVID-19 and healthy individuals. To this end, we visualized changes in co-expression between conditions and constructed co-expression networks for each condition. Firstly, we constructed protein-protein correlation matrices per condition. Subsequently, hierarchical clustering was performed to identify strongly co-expressed protein clusters (**Figure 5A**). ICU and non-ICU patients displayed comparable clusters of co-expressed proteins. However, this was not consistent over all conditions as there was little overlap with co-expressed protein clusters in post-COVID-19 and healthy individuals.

Subsequently, we constructed co-expression networks to identify hub proteins per condition, which in turn may help us understand the molecular basis of COVID-19 disease and recovery (**Figure 5B**). Protein co-expression clusters in hospitalized patients were distinct and non-overlapping compared to post-COVID-19 and healthy individuals. Measurement of betweenness centrality suggests that metalloproteinase inhibitor 1 (TIMP1), vascular endothelial growth factor A (VEGFA), TRAIL-R2, CD40, latency-associated transforming growth factor (LAP-TGF-ß), galectin-9 (Gal-9), TNFα, CD46, and thrombomodulin (TM) were hub proteins in ICU and non-ICU networks. Hub proteins for post-COVID-19 individuals included cytastatin 3 (CST3), Notch homolog 1, translocation-associated (NOTCH1), complement factor H-related 5 (CFHR5), and ST6 beta-galactoside alpha-2,6-sialyltransferase 1 (ST6GAL1). Interestingly, TIMP1 was detected as a hub protein in three out of four networks. TIMP1 has been shown to interact with MMPs and inhibit the proteolytic activity of MMPs in the ECM (33). Furthermore, strongly co-expressed clusters with CD40 as hub protein were detected in both ICU and non-ICU patients (marked clusters in **Figure 5B**). In both networks, this cluster contained several proteins related to TNF signaling pathways, such as TNF receptor superfamily member 9 (TNFRSF9, CD137), 11A (TNFRSF11A, RANK), 10A (TNFRSF10A, TRAIL-R1)), and TRAIL-R2. These proteins have been related to various biological processes such as apoptosis, cell proliferation & differentiation (34). Interestingly, this cluster was not detected in post-COVID-19 or healthy individuals. While the individual proteins were contained within all networks, no strong co-expression of these proteins was observed in post-COVID-19/healthy individuals.

## 4 Discussion

**Figure 6:**
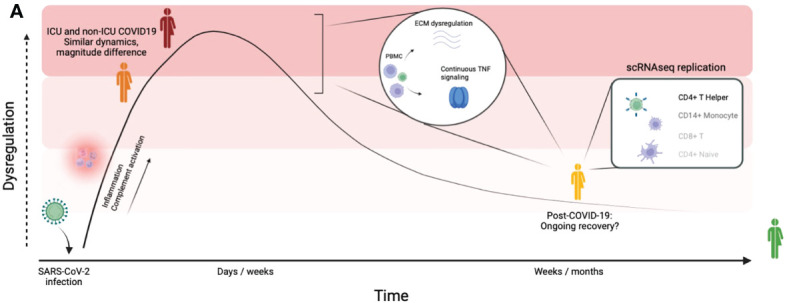
Graphical summary of our findings. Graphical summary of the main findings in this study. By integrating a large disease spectrum including variation in disease severity, course and recovery we show the proteomic changes induced by SARS-CoV-2 infection during the acute phase and the post-COVID-19 phase.

In this study, we associated circulating protein concentrations in COVID-19 hospitalized patients, post-COVID-19 individuals and healthy individuals to disease severity and time after SARS-CoV-2 infection in three independent, large cohorts. Altogether, the data reveal the proteomic changes induced by SARS-CoV-2 infection both during the acute phase and after the infection phase. The main findings in our study are presented in **Figure 6**. In addition to differential abundance analysis, we showed that circulating proteins are associated to disease severities across cohorts, and that the proteome is associated to the time after SARS-CoV-2 infection. Good predictive power of marker proteins suggests a robust contribution to the disease and argues that these proteins are therefore important to further investigate in future studies.

One of the most interesting findings of this study is the identification of a SARS-CoV-2 acute infection signature and a post-COVID-19 signature. The acute effects of SARS-CoV-2 are mainly marked by dysregulation of inflammatory markers and complement factors, as has been described before (9, 35). Consistent with earlier research in COVID-19 patients after the infection phase (12), our results suggest that a substantial proportion of proteins (109/220, ~50%) return to normal levels as they do not show statistical difference compared to controls in the post-infection phase. A note of caution for this conclusion is that we cannot completely exclude that for some proteins this may be caused by other factors such as a lack of statistical power to detect differences. The remaining post-COVID-19 signature shows good agreement with single-cell RNA sequencing data and contains, among others, proteins related to TGFβ signaling, maintenance of the ECM (TGFβ1, BMP-6, MMP1, MMP7), and TNF-signaling (TNFα, TWEAK). These signaling pathways have been related to other chronic inflammatory disease such as chronic fatigue syndrome (36, 37) and Q-fever fatigue syndrome (38), as well as pulmonary complications such as pulmonary fibrosis (39, 40), ARDS (41, 42) and asthma (43). Our data indicates that these signaling pathways regulate important biological processes that remain dysregulated several weeks after SARS-CoV-2 infection. It remains an open question whether modulation of these broad pathways could be beneficial for COVID-19 patients with chronic symptoms.

It is pertinent to note that many of the proteins discussed in this study have been described before in the context of acute COVID-19, attesting to their importance in this disease. Among others, we identified HGF, TWEAK, MMPs and TNFα as proteins that still remain enhanced after the infection phase of the disease. HGF promotes alveolar epithelial and endothelial repair after acute lung injury and was reported to be significantly upregulated in COVID-19 patients (44, 45). TWEAK, a negative regulator of interferon-γ (IFN-γ), was significantly elevated in COVID-19 patients compared to healthy controls and associated with disease severity (46, 47). Together with the current study, these findings may reflect the diminished IFN-response (48) and impaired T-cell functionality observed in COVID-19 (13, 49). MMPs, involved in the proteolytic degradation and maintenance of the ECM, as well as ECM-receptor interactions were found to be enhanced in acute COVID-19 (50, 51). Our analyses suggest these pathways remain enhanced during post-COVID-19.

Our study showed a consistent upregulation of MMPs (MMP1, MMP7, MMP10, and MMP12) in post-COVID-19 individuals, suggesting ongoing remodeling processes in individuals that experienced mild disease (52, 53). Previous research has shown the infiltration of profibrotic macrophages in the lung tissue of severe COVID-19 patients, and also highlighted MMPs as key mediators (54). Furthermore, other respiratory viruses such as Influenza and respiratory syncytial virus (RSV) induce upregulation of MMPs, suggesting similar pathways are influenced by different viruses (55, 56). Our study also identified uPA to be of importance of hospitalized COVID-19 patients. This is important because the interplay between MMPs and uPA, regulated by TGF-ß, plays a key role in the proteolytic degradation of the ECM (57). Progression of COVID-19 patients to respiratory insufficiency has been associated to uPA (58), and uPA-guided treatment with anakinra has proved successful in a large phase 3 randomized trial (59). Further research should consider the mechanisms through which disturbed regulation of the ECM persists after COVID-19 infection, and whether these pathways provide potential therapeutic targets.

Finally, our results support previous findings that highlight TNFα as a key mediator of disease in acute COVID-19 (32, 42). TNFα is a major regulator of immunological processes and has been reported in RSV as a mediator in infection-related illness (60). TNFα, among other pro-inflammatory cytokines, was also upregulated following influenza infection *in vitro* (61). We observed significant dysregulation of TNFα and closely related mediators of the TNF signaling pathways (TNFα, TWEAK, TRAIL and TRANCE) in ICU and non-ICU patients compared to healthy individuals. Strikingly, our data showed that those same proteins remain upregulated in post-COVID-19 individuals. Furthermore, we have shown that TNFα is closely connected to many dysregulated proteins in the post-infection phase, although these results have to be interpreted with caution since TNFα regulates many functions also under healthy conditions. Nevertheless, other studies have associated TNF-mediated inflammation with chronic fatigue before (36), which is one of the most commonly reported symptoms in individuals who suffer from long-lasting COVID-19 symptoms (3).

While most randomized trials in COVID-19 have focused in modulating the IL-1/IL-6 pathway, data from multiple studies suggest that anti-TNF treatments may also play a role in the therapeutic armamentarium (32, 42). At this time, the randomized trials still focus on immunotherapy in acute COVID-19: in contrast, our data suggests continuous upregulation of TNFα after the infection phase. The effect of anti-TNF in long COVID-19 patients could be a potential target for further research to establish the relationship between TNFα and COVID-19 recovery.

Despite the high impact of this research, several important limitations should be considered. First of all, although bridging sample normalization has been performed to alleviate the batch effects between cohorts, comparison between conditions (hospitalized versus post-COVID-19/healthy) could have been confounded between cohorts. Nevertheless, we did not observe significant batch effects within conditions that were properly divided across cohorts. Second, our healthy/post-COVID-19 cohort is younger compared to COVID-19 hospitalized patients, presenting a possible confounding factor. Third, proteomics data on its own are not able to provide a complete overview of COVID-19 pathology and recovery, requiring integrating with other type of omics data (e.g. transcriptome, metabolome) to uncover COVID-19 progression and recovery mechanisms. Fourth, most samples are of Western European ancestry, which may limit the generalization of our conclusions to populations of other ancestries. Fifth, our study demonstrated the associations between circulating protein concentrations, COVID-19 disease severity and time after infection. We are not able to relate the protein concentrations to chronic COVID-19 symptoms since the enrolled post-COVID-19 patients were largely symptom-free. Further research is needed to establish causal relationship between phenotypes and biomarkers. Lastly, individuals from our acute COVID-19 cohorts were admitted into care at the peak of the pandemic in 202022. In this hectic period, ICU/non-ICU stratification was the standard, and we continue with this stratification in the current work. Complementary clinical information could be used in future studies to assist in patient stratification.

In summary, our study provides insight into the changes induced by SARS-CoV-2 infection at the proteomic level by incorporating a large disease spectrum, including variation in disease severity, course and recovery. We highlight the protein biomarker candidates and biological pathways affected immediately upon infection and those that remain perturbed after viral clearance. Acute COVID-19 is primarily characterized by activation of the complement system and inflammatory markers. Post-COVID-19 individuals show continued perturbation of proteins related to maintenance of the ECM and TNF signaling. These results suggest ongoing remodeling processes and inflammation during post-COVID-19. Further research and patient cohorts are needed to investigate the precise connection between these biological pathways and the lingering COVID-19 symptoms previously reported.

## Data availability statement

The targeted proteomics data and code generated to process the data as well as the trained machine learning prediction models are freely available on Github (https://github.com/CiiM-Bioinformatics-group/Postcovid-targeted-proteomics).

## Ethics statement

The studies involving human participants were reviewed and approved by the local independent ethical committees. Approval numbers: CMO 2020-6344 and CMO 2016-2923 for cohort 2. Sample collection for cohort 3 (Hannover, Germany) was approved by the Internal Review Board of Hannover Medical School (approval number 3639_2017, 9001_BO-K, 9255_BO_K_2020). Samples in cohort 1 (Breda, the Netherlands) concerned secondary use of human tissue, and therefore no ethical approval was sought. The patients/participants provided their written informed consent to participate in this study.

## Author contributions

Conceptualization and design: YL, MGN, and BE-V. Data analysis: MZ, supervised by YL and C-JX. Patient recruitment, collection of biological material and experimental work: AN and IG. Patient recruitment and data interpretation: FV, LJ, AB, VK, GK, OB, NJ, MK, JD-A, AG, AE, AV, PP, RB, GB, and NG. Writing – original draft: YL, MGN, BE-V, C-JX, MZ, and MKG. Writing – review and editing: All co-authors. All authors contributed to the article and approved the submitted version.

## Funding

YL was supported by an ERC starting Grant (948207) and a Radboud University Medical Centre Hypatia Grant (2018). C-JX was supported by Helmholtz Initiative and Networking Fund (1800167). MN was supported by an ERC Advanced Grant (833247) and a Spinoza Grant of the Netherlands Organization for Scientific Research. This research was moreover funded in part by grants from the state of Lower Saxony (14 - 76103-184 CORONA-12/20) and the Federal Ministry of Health (ZMVI1-2520COR804). This work was partly supported by the German Federal Ministry of Education and Research NaFoUniMedCovid19”—COVIM (01KX2021).

## Acknowledgments

The authors would like to extend their gratitude to all study participants. Moreover, the authors would like to thank Milena Stietzel, Dörthe Rokitta, Nicole Neumann, Juliane Ebersold, Metodi V. Stankov, and Sophie Meyer for technical assistance in performing the experiments and Lana Sarkar and Helga Dijkstra for their support in sample collection and handling. The authors would like to thank Olink Proteomics AB (Uppsala Sweden) for their donation of multiplex proximity extension assays. Finally, the authors would like to thank the Radboudumc Center for Infectious Diseases COVID-19 study group: Helga Dijkstra, Heidi Lemmers, Liesbeth van Emst, Kiki Schraa, Cor Jacobs, Anneke Hijmans, Trees Jansen, Fieke Weren, Liz Fransman, Jelle Gerretsen, Josephine van de Maat, Gerine Nijman, Simone Moorlag, Esther Taks, Priya Debisarun, Ilse Kouijzer, Heiman Wertheim, Joost Hopman, Janette Rahamat-Langendoen, Chantal Bleeker-Rovers, Jaap ten Oever, Reinout van Crevel, Jos van der Meer, Marcel van Deuren, Jacobien Hoogerwerf, Quirijn de Mast, Monique Reijers, Wouter Hoefsloot, Michel van den Heuvel, Hans van der Hoeven, Hans Koenen, Ruben Smeets, Irma Joosten, Xuehui He, Collins Boahen, Valerie Koeken, Vasiliki Matzaraki, Vinod Kumar, Tim Frenzel, Roger Brüggemann, Jeroen Schouten, Pleun Hemelaar, Remi Beunders, Sjef van der Velde, Emma Kooistra, Nicole Waalders, Wout Claassen, Hidde Heesakkers, Tirsa van Schaik, Hetty van der Eng, Noortje Rovers, and Margreet Klop-Riehl.

## Conflict of interest

The authors declare that the research was conducted in the absence of any commercial or financial relationships that could be construed as a potential conflict of interest.

## Publisher’s note

All claims expressed in this article are solely those of the authors and do not necessarily represent those of their affiliated organizations, or those of the publisher, the editors and the reviewers. Any product that may be evaluated in this article, or claim that may be made by its manufacturer, is not guaranteed or endorsed by the publisher.
