# Supplementary material for: Targeted proteomics identifies circulating biomarkers associated with active COVID-19 and post-COVID-19
Source: Front Immunol. 2022 Nov 3;13:1027122. doi: 10.3389/fimmu.2022.1027122 (PMC9670186; doi:10.3389/fimmu.2022.1027122)
Supplement: Supplementary file 1 [file DataSheet_1.docx]

Supplementary Material

# Supplementary figures

Fig. S1: COVID-19 antibody concentrations in post-COVID-19 patients

**A)** Two antibody concentrations were measured in post-COVID-19 patients and healthy individuals: SARS-CoV Spike 1 (S1) and nucleocapsid protein (NCP). Antibody amounts are expressed as IgG ratio (optical density divided by calibrator). Significance was calculated using the non-parametric Wilcoxon test. Stars indicate significance: * = p < 0.05, ** = p < 0.01, *** = p < 0.001, **** = p. < 0.0001. Boxplot center line: median, box limits: 1^st^ and 3^rd^ quartiles. Whiskers: 1,5 x interquartile range. Measurements outside the range of the whiskers are depicted as single points

Fig. S2: Targeted protein measurements are consistent across panels

**A)** Correlations of five duplicate proteins measured across the Olink panels Cardiovascular II and Inflammation within cohort 1 (Nijmegen). Each dot represents a sample. The grey line is a linear regression model fitted to calculate the adjusted coefficient of determination R^2^.

**B)** Correlations of five duplicate proteins measured across the Olink panels Cardiovascular II and Inflammation within cohort 3 (Hannover). Each dot represents a sample. The grey line is a linear regression model fitted to calculate the adjusted coefficient of determination R^2^.

#

Fig. S3: Confusion matrices for the COVID-19 disease severity classification model

**A)** Conditions on the x-axis represent the ground truth values. Conditions on the y axis represent the predicted values from the fitted model. Cells are colored with respect to their counts. The left matrix is produced by the model from the training data (cohort 1, n = 168 COVID-19 ICU patients and 308 non-ICU), where the model achieved 98% accuracy (sensitivity: 91%, specific: 100%). the validation cohort, the model was 83% accurate (sensitivity: 0.76, specificity: 0.86).

**B**

**A**

Fig. S4: Replication of the post-COVID-19 proteome signature in publicly available single-cell RNA sequencing data.

**A)** Cell counts in the single-cell RNAseq dataset. Celltypes are ordered per abundance, and filled by the disease severity post-COVID-19 individuals experienced.

**B)** Barplots showing the percentage of differentially expressed proteins that were replicated in single-cell RNA sequencing data. We replicated our data in post-COVID-19 patients who experienced three different COVID-19 severities: Mild, Moderate or Severe.

# Supplementary tables

Table S1: Baseline demographics of cohort 1 (Breda)

|  | ICU | Non-ICU | Total |
| --- | --- | --- | --- |
| **N** | 59 | 148 | 207 |
|  |  |  |  |
| **General** |  |  |  |
| Gender (m, %) | 45 (76%) | 88 (59%) | 133 (64%) |
| Age (mean, sd) | 67 (9) | 70 (12) | 68 (11) |
| BMI (mean, sd) | 28 (4) | 28 (5) | 28 (4) |
|  |  |  |  |
| **Medical history** |  |  |  |
| Data available (n, %) | 53 (90%) | 98 (66%) | 151 (73%) |
|  |  |  |  |
| COPD (n, %) | 11 (21%) | 17 (32%) | 28 (18%) |
| Heart failure (n, %) | 14 (26%) | 26 (49%) | 40 (26%) |
| Renal failure (n, %) | 2 (4%) | 6 (11%) | 8 (5%) |
| Diabetes (n, %) | 8 (15%) | 22 (41%) | 30 (20%) |

Table S2: Baseline demographics of cohort 2 (Radboud)

|  | **ICU** | **Non-ICU** | **Total** |
| --- | --- | --- | --- |
| **N** | 37 | 106 | 143 |
|  |  |  |  |
| **General** |  |  |  |
| Gender (male n, %) | 28 (75%) | 69 (65%) | 97 (68%) |
| Age (mean, sd) | 64 (12) | 63 (13) |  |
| Weight (mean, sd) | 88 (14) | 78 (34) |  |
| BMI (mean, sd) | 27 (4) | 27 (4) |  |
|  |  |  |  |
| Ethnicity (n, %) |  |  |  |
| Caucasian | 16 (43%) | 51 | 67 (43%) |
| Black or sub-saharan African | 0 (0%) | 1 (1%) | 1 (1%) |
| Middle Eastern | 0 (0%) | 1 (1%) | 1 (1%) |
| Unknown | 21 (56%) | 53 (50%) | 74 (52%) |
|  |  |  |  |
| Smoking (n, %) |  |  |  |
| No, never | 9 (24%) | 39 (37%) | 48 (34%) |
| Yes, current | 2 (5%) | 7 (7%) | 9 (6%) |
| Yes, Former | 12 (33%) | 38 (36%) | 50 (35%) |
| Unknown | 14 (38%) | 22 (21%) | 36 (25%) |
|  |  |  |  |
| **Medical history** |  |  |  |
| Data available (n) |  |  |  |
|  |  |  |  |
| Pulmonary disease including COPD | 4 (10%) | 29 (27%) | 33 (23%) |
| Diabetes | 10 (27%) | 18 (17%) | 28 (20%) |
| Cardiovascular disease | 19 (51%) | 59 (56%) | 78 (55%) |
| Chronic renal disease (requiring renal replacement) | 0 (0%) | 1 (1%) | 1 (1%) |
| Chronic kidney disease (not requiring renal replacement) | 0 (0%) | 6 (6%) | 6 (4%) |
| Autoimmune disease including IBD (n, %) | 0 (0%) | 15 (14%) | 15 (10%) |
| HIV/AIDS | 0 (0%) | 1 (1%) | 1 (1%) |

Table S3: Baseline demographics of cohort 3 (Hannover)

|  | Post-COVID-19 | Healthy | Total |
| --- | --- | --- | --- |
| **N** | 186 | 61 | 247 |
|  |  |  |  |
| **General** |  |  |  |
| Gender (m, %) | 99 | 36 | 135 (55%) |
| Age (mean, sd) | 43 (12) | 46 (14) | 44 (13) |
|  |  |  |  |
| **Medical history** |  |  |  |
| Data available (n, %) | 170 (91%) | 0 (0%) | 170 (70%) |
|  |  |  |  |
| Asthma | 4 (0%) | 0 (0%) | 4 (0%) |
| High blood pressure | 7 (0%) | 0 (0%) | 7 (0%) |
| Partial thyroid gland, grade 1 ERD. | 1 (0%) | 0 (0%) | 1 (0%) |

Table S4: Overview of the cohorts included in this study.

| **Cohort** | **Origin** | **Cohort size** | **Nr.Proteins measured** | **Disease status** | **Data** | **Status** |
| --- | --- | --- | --- | --- | --- | --- |
| Cohort 1 | Breda (NL) | 207 | 92 | ICU / non-ICU | Cross-sectional + longitudinal | **New data** |
| Cohort 2 | Nijmegen (NL) | 143 | 276 | ICU / non-ICU | Cross-sectional + longitudinal | Published data^#^ |
| Cohort 3 | Hannover (DE) | 247 | 368 | Post-COVID-19 / healthy | Cross-sectional | **New data** |

Table S5: Differential protein abundance results

| **OlinkID** | **Assay** | **Olink.panel** | **logFC** | **P.Value** | **adj.P.Val** | **comparison** |
| --- | --- | --- | --- | --- | --- | --- |
| OID00522 | HGF | Olink INFLAMMATION | 1,413372826 | 1,25111E-14 | 7,88202E-13 | ICU_vs_nonICU_breda |
| OID00541 | EN-RAGE | Olink INFLAMMATION | 1,385965336 | 3,35436E-11 | 1,05662E-09 | ICU_vs_nonICU_breda |
| OID00500 | SCF | Olink INFLAMMATION | -1,01119832 | 9,80085E-09 | 2,05818E-07 | ICU_vs_nonICU_breda |
| OID00556 | CCL20 | Olink INFLAMMATION | 1,22832885 | 1,58956E-08 | 2,50356E-07 | ICU_vs_nonICU_breda |
| OID00490 | CXCL9 | Olink INFLAMMATION | 1,034026169 | 2,32374E-08 | 2,92791E-07 | ICU_vs_nonICU_breda |
| OID00513 | CCL19 | Olink INFLAMMATION | 0,954445988 | 3,49927E-08 | 3,67424E-07 | ICU_vs_nonICU_breda |
| OID00484 | MCP-1 | Olink INFLAMMATION | 0,854133523 | 8,84085E-08 | 7,95676E-07 | ICU_vs_nonICU_breda |
| OID00503 | TGF-alpha | Olink INFLAMMATION | 0,564002673 | 1,84755E-07 | 1,45494E-06 | ICU_vs_nonICU_breda |
| OID00479 | OPG | Olink INFLAMMATION | 0,446196261 | 1,38288E-06 | 9,68018E-06 | ICU_vs_nonICU_breda |
| OID00532 | CCL3 | Olink INFLAMMATION | 0,69043513 | 1,7324E-06 | 1,09141E-05 | ICU_vs_nonICU_breda |
| OID00527 | MMP-10 | Olink INFLAMMATION | 0,644419757 | 5,91921E-06 | 3,39009E-05 | ICU_vs_nonICU_breda |
| OID01213 | DNER | Olink INFLAMMATION | -0,263943331 | 1,05814E-05 | 5,55524E-05 | ICU_vs_nonICU_breda |
| OID00471 | IL8 | Olink INFLAMMATION | 0,824983691 | 4,57149E-05 | 0,000221542 | ICU_vs_nonICU_breda |
| OID00542 | CD40 | Olink INFLAMMATION | 0,432016323 | 0,000112925 | 0,000508161 | ICU_vs_nonICU_breda |
| OID00517 | IL-18R1 | Olink INFLAMMATION | 0,333610712 | 0,000140948 | 0,00058225 | ICU_vs_nonICU_breda |
| OID00476 | CDCP1 | Olink INFLAMMATION | 0,472869553 | 0,000152147 | 0,00058225 | ICU_vs_nonICU_breda |
| OID00494 | OSM | Olink INFLAMMATION | 0,884152702 | 0,000157115 | 0,00058225 | ICU_vs_nonICU_breda |
| OID00472 | VEGFA | Olink INFLAMMATION | 0,3607043 | 0,000173256 | 0,000606398 | ICU_vs_nonICU_breda |
| OID00518 | PD-L1 | Olink INFLAMMATION | 0,366845569 | 0,000206974 | 0,000686283 | ICU_vs_nonICU_breda |
| OID00506 | TNFSF14 | Olink INFLAMMATION | 0,477783489 | 0,001611533 | 0,005076328 | ICU_vs_nonICU_breda |
| OID00498 | CCL4 | Olink INFLAMMATION | 0,408229803 | 0,003331168 | 0,009993505 | ICU_vs_nonICU_breda |
| OID00481 | uPA | Olink INFLAMMATION | 0,242344569 | 0,004530745 | 0,012254498 | ICU_vs_nonICU_breda |
| OID00515 | IL-10RB | Olink INFLAMMATION | 0,213889051 | 0,004552048 | 0,012254498 | ICU_vs_nonICU_breda |
| OID00530 | CCL23 | Olink INFLAMMATION | 0,273412829 | 0,00466838 | 0,012254498 | ICU_vs_nonICU_breda |
| OID00555 | TWEAK | Olink INFLAMMATION | -0,206807182 | 0,007382822 | 0,018604712 | ICU_vs_nonICU_breda |
| OID00512 | FGF-21 | Olink INFLAMMATION | 0,86811497 | 0,00985992 | 0,023891344 | ICU_vs_nonICU_breda |
| OID00504 | MCP-4 | Olink INFLAMMATION | 0,418947286 | 0,010985222 | 0,025632185 | ICU_vs_nonICU_breda |
| OID00520 | CXCL5 | Olink INFLAMMATION | -0,623196255 | 0,011621468 | 0,026148302 | ICU_vs_nonICU_breda |
| OID00561 | TNFB | Olink INFLAMMATION | -0,304004879 | 0,012781005 | 0,027765632 | ICU_vs_nonICU_breda |
| OID00491 | CST5 | Olink INFLAMMATION | 0,314766204 | 0,014517455 | 0,030486656 | ICU_vs_nonICU_breda |
| OID00488 | TRAIL | Olink INFLAMMATION | -0,215216611 | 0,036235857 | 0,072262018 | ICU_vs_nonICU_breda |
| OID00553 | TNFRSF9 | Olink INFLAMMATION | 0,343746567 | 0,036704517 | 0,072262018 | ICU_vs_nonICU_breda |
| OID00501 | IL18 | Olink INFLAMMATION | 0,28635154 | 0,049099218 | 0,09373487 | ICU_vs_nonICU_breda |
| OID00477 | CD244 | Olink INFLAMMATION | -0,141471276 | 0,057131308 | 0,104843892 | ICU_vs_nonICU_breda |
| OID00521 | TRANCE | Olink INFLAMMATION | -0,235175919 | 0,058246607 | 0,104843892 | ICU_vs_nonICU_breda |
| OID05548 | TNF | Olink INFLAMMATION | 0,208796015 | 0,061046835 | 0,106831962 | ICU_vs_nonICU_breda |
| OID05124 | CD8A | Olink INFLAMMATION | -0,274810521 | 0,086079026 | 0,146566991 | ICU_vs_nonICU_breda |
| OID00549 | MCP-2 | Olink INFLAMMATION | 0,325881111 | 0,095057918 | 0,157596023 | ICU_vs_nonICU_breda |
| OID00499 | CD6 | Olink INFLAMMATION | -0,195885148 | 0,109236691 | 0,175058317 | ICU_vs_nonICU_breda |
| OID00511 | LIF-R | Olink INFLAMMATION | -0,111127047 | 0,112065518 | 0,175058317 | ICU_vs_nonICU_breda |
| OID05547 | IFN-gamma | Olink INFLAMMATION | -0,662475582 | 0,113926841 | 0,175058317 | ICU_vs_nonICU_breda |
| OID00562 | CSF-1 | Olink INFLAMMATION | 0,045931747 | 0,176055213 | 0,264082819 | ICU_vs_nonICU_breda |
| OID00560 | ADA | Olink INFLAMMATION | 0,131301289 | 0,196656849 | 0,283829316 | ICU_vs_nonICU_breda |
| OID00510 | MMP-1 | Olink INFLAMMATION | 0,342163796 | 0,200218612 | 0,283829316 | ICU_vs_nonICU_breda |
| OID00545 | FGF-19 | Olink INFLAMMATION | 0,271612652 | 0,202735226 | 0,283829316 | ICU_vs_nonICU_breda |
| OID00533 | Flt3L | Olink INFLAMMATION | 0,12575877 | 0,269219497 | 0,368713659 | ICU_vs_nonICU_breda |
| OID00550 | CASP-8 | Olink INFLAMMATION | 0,131136553 | 0,336436443 | 0,447373452 | ICU_vs_nonICU_breda |
| OID00505 | CCL11 | Olink INFLAMMATION | -0,101432358 | 0,340855963 | 0,447373452 | ICU_vs_nonICU_breda |
| OID00535 | CXCL10 | Olink INFLAMMATION | 0,140925642 | 0,407872531 | 0,52440754 | ICU_vs_nonICU_breda |
| OID00531 | CD5 | Olink INFLAMMATION | 0,086691482 | 0,417002515 | 0,525423169 | ICU_vs_nonICU_breda |
| OID00486 | CXCL11 | Olink INFLAMMATION | -0,136342268 | 0,45067964 | 0,556721908 | ICU_vs_nonICU_breda |
| OID00478 | IL7 | Olink INFLAMMATION | 0,086921514 | 0,474467345 | 0,574835437 | ICU_vs_nonICU_breda |
| OID00487 | AXIN1 | Olink INFLAMMATION | -0,092340936 | 0,596696053 | 0,686835681 | ICU_vs_nonICU_breda |
| OID00496 | CXCL1 | Olink INFLAMMATION | -0,088113378 | 0,598301566 | 0,686835681 | ICU_vs_nonICU_breda |
| OID00523 | IL-12B | Olink INFLAMMATION | 0,102722282 | 0,59970148 | 0,686835681 | ICU_vs_nonICU_breda |
| OID00539 | CCL28 | Olink INFLAMMATION | 0,041795273 | 0,615108222 | 0,686835681 | ICU_vs_nonICU_breda |
| OID00536 | 4E-BP1 | Olink INFLAMMATION | 0,069158969 | 0,621422759 | 0,686835681 | ICU_vs_nonICU_breda |
| OID00551 | CCL25 | Olink INFLAMMATION | 0,061888214 | 0,66835902 | 0,725976177 | ICU_vs_nonICU_breda |
| OID00558 | STAMBP | Olink INFLAMMATION | -0,049113141 | 0,73352485 | 0,783255348 | ICU_vs_nonICU_breda |
| OID00557 | ST1A1 | Olink INFLAMMATION | -0,022698522 | 0,90943782 | 0,939736105 | ICU_vs_nonICU_breda |
| OID00534 | CXCL6 | Olink INFLAMMATION | 0,016389711 | 0,909903212 | 0,939736105 | ICU_vs_nonICU_breda |
| OID00480 | LAP TGF-beta-1 | Olink INFLAMMATION | -0,001985835 | 0,983233697 | 0,991886643 | ICU_vs_nonICU_breda |
| OID00552 | CX3CL1 | Olink INFLAMMATION | -0,001113139 | 0,991886643 | 0,991886643 | ICU_vs_nonICU_breda |
| OID00522 | HGF | Olink INFLAMMATION | 1,627550204 | 5,37579E-17 | 1,18805E-14 | ICU_vs_nonICU_radboud |
| OID00556 | CCL20 | Olink INFLAMMATION | 1,848454489 | 2,18682E-16 | 2,41644E-14 | ICU_vs_nonICU_radboud |
| OID00408 | SCF | Olink CARDIOVASCULAR II | -1,29957356 | 2,84319E-14 | 2,09448E-12 | ICU_vs_nonICU_radboud |
| OID00500 | SCF | Olink INFLAMMATION | -1,208713645 | 1,91455E-12 | 1,05779E-10 | ICU_vs_nonICU_radboud |
| OID01306 | ANGPTL3 | Olink CARDIOMETABOLIC | 0,598871461 | 9,24441E-11 | 4,08603E-09 | ICU_vs_nonICU_radboud |
| OID00396 | TRAIL-R2 | Olink CARDIOVASCULAR II | 1,095071467 | 1,59381E-10 | 5,87054E-09 | ICU_vs_nonICU_radboud |
| OID00468 | VEGFD | Olink CARDIOVASCULAR II | -0,700365234 | 3,34399E-10 | 1,05575E-08 | ICU_vs_nonICU_radboud |
| OID00513 | CCL19 | Olink INFLAMMATION | 1,147685218 | 5,12032E-10 | 1,41449E-08 | ICU_vs_nonICU_radboud |
| OID01213 | DNER | Olink INFLAMMATION | -0,389456914 | 6,78094E-10 | 1,6651E-08 | ICU_vs_nonICU_radboud |
| OID00479 | OPG | Olink INFLAMMATION | 0,553536805 | 1,33451E-09 | 2,94926E-08 | ICU_vs_nonICU_radboud |
| OID01221 | APOM | Olink CARDIOMETABOLIC | -0,622251951 | 2,97878E-09 | 5,98463E-08 | ICU_vs_nonICU_radboud |
| OID01224 | TIMP1 | Olink CARDIOMETABOLIC | 0,573132764 | 7,28337E-09 | 1,34135E-07 | ICU_vs_nonICU_radboud |
| OID00541 | EN-RAGE | Olink INFLAMMATION | 1,479650656 | 8,16063E-09 | 1,38731E-07 | ICU_vs_nonICU_radboud |
| OID00484 | MCP-1 | Olink INFLAMMATION | 0,962121036 | 3,69445E-08 | 5,83196E-07 | ICU_vs_nonICU_radboud |
| OID00488 | TRAIL | Olink INFLAMMATION | -0,554930893 | 5,06812E-08 | 7,46703E-07 | ICU_vs_nonICU_radboud |
| OID00459 | CTSL1 | Olink CARDIOVASCULAR II | 0,680038334 | 6,75574E-08 | 9,33136E-07 | ICU_vs_nonICU_radboud |
| OID00476 | CDCP1 | Olink INFLAMMATION | 0,610309668 | 8,40406E-08 | 1,09253E-06 | ICU_vs_nonICU_radboud |
| OID01302 | CFHR5 | Olink CARDIOMETABOLIC | 0,63072735 | 1,06476E-07 | 1,30728E-06 | ICU_vs_nonICU_radboud |
| OID01265 | TNC | Olink CARDIOMETABOLIC | 0,86527883 | 4,11796E-07 | 4,78983E-06 | ICU_vs_nonICU_radboud |
| OID00472 | VEGFA | Olink INFLAMMATION | 0,546019849 | 1,07948E-06 | 1,14044E-05 | ICU_vs_nonICU_radboud |
| OID00471 | IL8 | Olink INFLAMMATION | 0,871671702 | 1,13397E-06 | 1,14044E-05 | ICU_vs_nonICU_radboud |
| OID00517 | IL-18R1 | Olink INFLAMMATION | 0,482456991 | 1,13528E-06 | 1,14044E-05 | ICU_vs_nonICU_radboud |
| OID00521 | TRANCE | Olink INFLAMMATION | -0,582495281 | 2,387E-06 | 2,2936E-05 | ICU_vs_nonICU_radboud |
| OID00512 | FGF-21 | Olink INFLAMMATION | 1,538610426 | 3,3347E-06 | 3,0707E-05 | ICU_vs_nonICU_radboud |
| OID01230 | ICAM1 | Olink CARDIOMETABOLIC | 0,387016266 | 3,96751E-06 | 3,50728E-05 | ICU_vs_nonICU_radboud |
| OID00532 | CCL3 | Olink INFLAMMATION | 0,649179716 | 4,63664E-06 | 3,94114E-05 | ICU_vs_nonICU_radboud |
| OID00423 | REN | Olink CARDIOVASCULAR II | 0,778649709 | 5,33508E-06 | 4,36686E-05 | ICU_vs_nonICU_radboud |
| OID01262 | GNLY | Olink CARDIOMETABOLIC | 0,404229032 | 6,10312E-06 | 4,81711E-05 | ICU_vs_nonICU_radboud |
| OID01231 | REG1A | Olink CARDIOMETABOLIC | 0,906525818 | 6,37616E-06 | 4,85908E-05 | ICU_vs_nonICU_radboud |
| OID01305 | FETUB | Olink CARDIOMETABOLIC | -0,603267883 | 7,74714E-06 | 5,4442E-05 | ICU_vs_nonICU_radboud |
| OID00530 | CCL23 | Olink INFLAMMATION | 0,507687202 | 7,80039E-06 | 5,4442E-05 | ICU_vs_nonICU_radboud |
| OID01301 | C1QTNF1 | Olink CARDIOMETABOLIC | 0,381394512 | 7,883E-06 | 5,4442E-05 | ICU_vs_nonICU_radboud |
| OID00381 | ADM | Olink CARDIOVASCULAR II | 0,34686748 | 9,74096E-06 | 6,52349E-05 | ICU_vs_nonICU_radboud |
| OID01229 | SERPINA5 | Olink CARDIOMETABOLIC | -0,62264046 | 1,05645E-05 | 6,86691E-05 | ICU_vs_nonICU_radboud |
| OID01298 | TIMD4 | Olink CARDIOMETABOLIC | 0,654074046 | 1,2669E-05 | 7,99954E-05 | ICU_vs_nonICU_radboud |
| OID00389 | IL-1ra | Olink CARDIOVASCULAR II | 0,626991022 | 1,39596E-05 | 8,56962E-05 | ICU_vs_nonICU_radboud |
| OID01269 | SAA4 | Olink CARDIOMETABOLIC | -0,595515702 | 1,68808E-05 | 0,000100828 | ICU_vs_nonICU_radboud |
| OID00518 | PD-L1 | Olink INFLAMMATION | 0,440016444 | 1,84203E-05 | 0,000107129 | ICU_vs_nonICU_radboud |
| OID00387 | IL-4RA | Olink CARDIOVASCULAR II | 0,54523778 | 2,27504E-05 | 0,000128919 | ICU_vs_nonICU_radboud |
| OID00410 | FGF-21 | Olink CARDIOVASCULAR II | 1,502060798 | 2,40168E-05 | 0,000132693 | ICU_vs_nonICU_radboud |
| OID01266 | DPP4 | Olink CARDIOMETABOLIC | -0,331131563 | 2,59505E-05 | 0,000139879 | ICU_vs_nonICU_radboud |
| OID00391 | TNFRSF10A | Olink CARDIOVASCULAR II | 0,48225802 | 2,68376E-05 | 0,000141217 | ICU_vs_nonICU_radboud |
| OID00503 | TGF-alpha | Olink INFLAMMATION | 0,450021637 | 3,45225E-05 | 0,00017743 | ICU_vs_nonICU_radboud |
| OID00440 | CCL3 | Olink CARDIOVASCULAR II | 0,618394152 | 3,83387E-05 | 0,000192565 | ICU_vs_nonICU_radboud |
| OID00499 | CD6 | Olink INFLAMMATION | -0,599214722 | 5,5133E-05 | 0,000270764 | ICU_vs_nonICU_radboud |
| OID01247 | NCAM1 | Olink CARDIOMETABOLIC | -0,218265216 | 0,000106603 | 0,000512159 | ICU_vs_nonICU_radboud |
| OID01250 | NID1 | Olink CARDIOMETABOLIC | 0,336816496 | 0,000117586 | 0,000552907 | ICU_vs_nonICU_radboud |
| OID01252 | ST6GAL1 | Olink CARDIOMETABOLIC | 0,372597975 | 0,000125535 | 0,000577982 | ICU_vs_nonICU_radboud |
| OID01244 | FCGR2A | Olink CARDIOMETABOLIC | 0,533808759 | 0,000152446 | 0,000687563 | ICU_vs_nonICU_radboud |
| OID00465 | HSP 27 | Olink CARDIOVASCULAR II | 0,284591699 | 0,000168179 | 0,000743352 | ICU_vs_nonICU_radboud |
| OID01307 | LYVE1 | Olink CARDIOMETABOLIC | 0,391872904 | 0,000198721 | 0,000861125 | ICU_vs_nonICU_radboud |
| OID01264 | IGFBP6 | Olink CARDIOMETABOLIC | -0,38182096 | 0,000217453 | 0,000924174 | ICU_vs_nonICU_radboud |
| OID00405 | LOX-1 | Olink CARDIOVASCULAR II | 0,553741109 | 0,000248853 | 0,00103767 | ICU_vs_nonICU_radboud |
| OID01274 | COMP | Olink CARDIOMETABOLIC | -0,435177429 | 0,000255158 | 0,001044257 | ICU_vs_nonICU_radboud |
| OID00527 | MMP-10 | Olink INFLAMMATION | 0,567439491 | 0,000323147 | 0,001267907 | ICU_vs_nonICU_radboud |
| OID00481 | uPA | Olink INFLAMMATION | 0,321068934 | 0,000323377 | 0,001267907 | ICU_vs_nonICU_radboud |
| OID01236 | PRSS2 | Olink CARDIOMETABOLIC | 0,770556011 | 0,000327017 | 0,001267907 | ICU_vs_nonICU_radboud |
| OID00416 | SPON2 | Olink CARDIOVASCULAR II | 0,120819016 | 0,000353511 | 0,001346999 | ICU_vs_nonICU_radboud |
| OID01217 | NRP1 | Olink CARDIOMETABOLIC | 0,166716469 | 0,00045496 | 0,001704171 | ICU_vs_nonICU_radboud |
| OID01235 | CA3 | Olink CARDIOMETABOLIC | 0,579985933 | 0,000553184 | 0,002037559 | ICU_vs_nonICU_radboud |
| OID00494 | OSM | Olink INFLAMMATION | 0,69837741 | 0,000608491 | 0,002182454 | ICU_vs_nonICU_radboud |
| OID01218 | PLXNB2 | Olink CARDIOMETABOLIC | 0,180687962 | 0,000612272 | 0,002182454 | ICU_vs_nonICU_radboud |
| OID01228 | PROC | Olink CARDIOMETABOLIC | -0,324026323 | 0,000658042 | 0,00230837 | ICU_vs_nonICU_radboud |
| OID01241 | KIT | Olink CARDIOMETABOLIC | -0,256866137 | 0,000726563 | 0,002508911 | ICU_vs_nonICU_radboud |
| OID01300 | OSMR | Olink CARDIOMETABOLIC | 0,16728824 | 0,00075794 | 0,002576996 | ICU_vs_nonICU_radboud |
| OID01223 | CA1 | Olink CARDIOMETABOLIC | -0,643098094 | 0,000810198 | 0,002712936 | ICU_vs_nonICU_radboud |
| OID00561 | TNFB | Olink INFLAMMATION | -0,40280448 | 0,000844542 | 0,002765152 | ICU_vs_nonICU_radboud |
| OID00462 | TGM2 | Olink CARDIOVASCULAR II | -0,337506742 | 0,000850816 | 0,002765152 | ICU_vs_nonICU_radboud |
| OID01290 | FCN2 | Olink CARDIOMETABOLIC | 0,373302693 | 0,00109763 | 0,003515598 | ICU_vs_nonICU_radboud |
| OID00510 | MMP-1 | Olink INFLAMMATION | 0,796607514 | 0,001199014 | 0,003785457 | ICU_vs_nonICU_radboud |
| OID01232 | SERPINA7 | Olink CARDIOMETABOLIC | -0,255200111 | 0,001463099 | 0,004554152 | ICU_vs_nonICU_radboud |
| OID00536 | 4E-BP1 | Olink INFLAMMATION | -0,483742871 | 0,001914917 | 0,005836823 | ICU_vs_nonICU_radboud |
| OID00400 | IL1RL2 | Olink CARDIOVASCULAR II | 0,299008471 | 0,001928 | 0,005836823 | ICU_vs_nonICU_radboud |
| OID00466 | CD4 | Olink CARDIOVASCULAR II | 0,237938762 | 0,002564966 | 0,007660236 | ICU_vs_nonICU_radboud |
| OID01243 | MBL2 | Olink CARDIOMETABOLIC | 0,69344286 | 0,002988539 | 0,008806229 | ICU_vs_nonICU_radboud |
| OID00399 | TF | Olink CARDIOVASCULAR II | 0,270761534 | 0,00316481 | 0,009202933 | ICU_vs_nonICU_radboud |
| OID00426 | KIM1 | Olink CARDIOVASCULAR II | 0,549723881 | 0,003344248 | 0,009598425 | ICU_vs_nonICU_radboud |
| OID00451 | FABP2 | Olink CARDIOVASCULAR II | -0,736479359 | 0,004068807 | 0,011528287 | ICU_vs_nonICU_radboud |
| OID00555 | TWEAK | Olink INFLAMMATION | -0,242478553 | 0,004291298 | 0,012004769 | ICU_vs_nonICU_radboud |
| OID00506 | TNFSF14 | Olink INFLAMMATION | 0,462172254 | 0,004513976 | 0,012469858 | ICU_vs_nonICU_radboud |
| OID00448 | AGRP | Olink CARDIOVASCULAR II | 0,38509564 | 0,004988794 | 0,013583849 | ICU_vs_nonICU_radboud |
| OID01293 | QPCT | Olink CARDIOMETABOLIC | 0,17348452 | 0,005040161 | 0,013583849 | ICU_vs_nonICU_radboud |
| OID00419 | GLO1 | Olink CARDIOVASCULAR II | -0,379340511 | 0,005507379 | 0,014664225 | ICU_vs_nonICU_radboud |
| OID01270 | TIE1 | Olink CARDIOMETABOLIC | 0,133902116 | 0,005752425 | 0,015025267 | ICU_vs_nonICU_radboud |
| OID00515 | IL-10RB | Olink INFLAMMATION | 0,192393291 | 0,005778949 | 0,015025267 | ICU_vs_nonICU_radboud |
| OID00562 | CSF-1 | Olink INFLAMMATION | 0,087577127 | 0,006137799 | 0,015772715 | ICU_vs_nonICU_radboud |
| OID00501 | IL18 | Olink INFLAMMATION | 0,392782823 | 0,006395219 | 0,016245327 | ICU_vs_nonICU_radboud |
| OID00542 | CD40 | Olink INFLAMMATION | 0,284577194 | 0,006537063 | 0,016416943 | ICU_vs_nonICU_radboud |
| OID00444 | DCN | Olink CARDIOVASCULAR II | 0,238776493 | 0,006692375 | 0,016618144 | ICU_vs_nonICU_radboud |
| OID01253 | IL7R | Olink CARDIOMETABOLIC | -0,243892993 | 0,006882333 | 0,016899952 | ICU_vs_nonICU_radboud |
| OID00490 | CXCL9 | Olink INFLAMMATION | 0,591195205 | 0,007503382 | 0,018222499 | ICU_vs_nonICU_radboud |
| OID01240 | IGLC2 | Olink CARDIOMETABOLIC | 0,366110545 | 0,008061378 | 0,019264928 | ICU_vs_nonICU_radboud |
| OID01299 | CNDP1 | Olink CARDIOMETABOLIC | -0,269687959 | 0,008150226 | 0,019264928 | ICU_vs_nonICU_radboud |
| OID01294 | AOC3 | Olink CARDIOMETABOLIC | -0,163474515 | 0,008194132 | 0,019264928 | ICU_vs_nonICU_radboud |
| OID01287 | SPARCL1 | Olink CARDIOMETABOLIC | -0,170664983 | 0,008740584 | 0,02018879 | ICU_vs_nonICU_radboud |
| OID00436 | CEACAM8 | Olink CARDIOVASCULAR II | 0,451248899 | 0,008778059 | 0,02018879 | ICU_vs_nonICU_radboud |
| OID01254 | ENG | Olink CARDIOMETABOLIC | -0,099798897 | 0,008861143 | 0,02018879 | ICU_vs_nonICU_radboud |
| OID00409 | IL18 | Olink CARDIOVASCULAR II | 0,377847503 | 0,00926147 | 0,02088556 | ICU_vs_nonICU_radboud |
| OID00486 | CXCL11 | Olink INFLAMMATION | 0,554427798 | 0,0094936 | 0,021192784 | ICU_vs_nonICU_radboud |
| OID00385 | ADAM-TS13 | Olink CARDIOVASCULAR II | -0,100098754 | 0,010557947 | 0,023333064 | ICU_vs_nonICU_radboud |
| OID00430 | AMBP | Olink CARDIOVASCULAR II | -0,137721221 | 0,011718062 | 0,025640512 | ICU_vs_nonICU_radboud |
| OID00437 | PTX3 | Olink CARDIOVASCULAR II | 0,271508162 | 0,015331767 | 0,033218829 | ICU_vs_nonICU_radboud |
| OID01227 | F11 | Olink CARDIOMETABOLIC | -0,189229881 | 0,015788302 | 0,033875872 | ICU_vs_nonICU_radboud |
| OID00422 | SERPINA12 | Olink CARDIOVASCULAR II | -0,510101763 | 0,017318342 | 0,036801476 | ICU_vs_nonICU_radboud |
| OID01259 | TCN2 | Olink CARDIOMETABOLIC | -0,26334711 | 0,017882414 | 0,037638224 | ICU_vs_nonICU_radboud |
| OID01303 | MEGF9 | Olink CARDIOMETABOLIC | -0,143103237 | 0,020004778 | 0,041708075 | ICU_vs_nonICU_radboud |
| OID01284 | PTPRS | Olink CARDIOMETABOLIC | -0,096739618 | 0,021420669 | 0,044242689 | ICU_vs_nonICU_radboud |
| OID00397 | PRSS27 | Olink CARDIOVASCULAR II | -0,267203904 | 0,024650012 | 0,050366294 | ICU_vs_nonICU_radboud |
| OID00531 | CD5 | Olink INFLAMMATION | -0,245423689 | 0,024841294 | 0,050366294 | ICU_vs_nonICU_radboud |
| OID01281 | EFEMP1 | Olink CARDIOMETABOLIC | -0,198142165 | 0,02554888 | 0,051330022 | ICU_vs_nonICU_radboud |
| OID00496 | CXCL1 | Olink INFLAMMATION | 0,364639196 | 0,029574987 | 0,058883533 | ICU_vs_nonICU_radboud |
| OID01249 | SELL | Olink CARDIOMETABOLIC | -0,196720196 | 0,030130244 | 0,059086616 | ICU_vs_nonICU_radboud |
| OID00477 | CD244 | Olink INFLAMMATION | -0,157828055 | 0,030240153 | 0,059086616 | ICU_vs_nonICU_radboud |
| OID00498 | CCL4 | Olink INFLAMMATION | 0,325748481 | 0,030479069 | 0,059086616 | ICU_vs_nonICU_radboud |
| OID00461 | TNFRSF13B | Olink CARDIOVASCULAR II | 0,414439664 | 0,0319538 | 0,061406867 | ICU_vs_nonICU_radboud |
| OID01234 | GP1BA | Olink CARDIOMETABOLIC | 0,202481806 | 0,033285732 | 0,063415058 | ICU_vs_nonICU_radboud |
| OID01226 | ANG | Olink CARDIOMETABOLIC | -0,181099743 | 0,033643916 | 0,06354962 | ICU_vs_nonICU_radboud |
| OID01258 | CR2 | Olink CARDIOMETABOLIC | -0,280954202 | 0,034554214 | 0,064715942 | ICU_vs_nonICU_radboud |
| OID00549 | MCP-2 | Olink INFLAMMATION | 0,461157656 | 0,037313581 | 0,06873905 | ICU_vs_nonICU_radboud |
| OID00434 | IL16 | Olink CARDIOVASCULAR II | -0,256128122 | 0,037324371 | 0,06873905 | ICU_vs_nonICU_radboud |
| OID00463 | LEP | Olink CARDIOVASCULAR II | -0,446206872 | 0,040440487 | 0,073862376 | ICU_vs_nonICU_radboud |
| OID00453 | MARCO | Olink CARDIOVASCULAR II | -0,11658933 | 0,04444096 | 0,080503706 | ICU_vs_nonICU_radboud |
| OID00388 | SRC | Olink CARDIOVASCULAR II | 0,42303874 | 0,049324235 | 0,088623219 | ICU_vs_nonICU_radboud |
| OID00535 | CXCL10 | Olink INFLAMMATION | 0,431275247 | 0,050675672 | 0,090317126 | ICU_vs_nonICU_radboud |
| OID00417 | GH | Olink CARDIOVASCULAR II | 0,448377554 | 0,051777246 | 0,091304349 | ICU_vs_nonICU_radboud |
| OID00428 | TM | Olink CARDIOVASCULAR II | 0,170728752 | 0,052055873 | 0,091304349 | ICU_vs_nonICU_radboud |
| OID01268 | THBS4 | Olink CARDIOMETABOLIC | -0,282791119 | 0,053853156 | 0,093712972 | ICU_vs_nonICU_radboud |
| OID00394 | TNFRSF11A | Olink CARDIOVASCULAR II | 0,250620467 | 0,056887122 | 0,098219171 | ICU_vs_nonICU_radboud |
| OID00454 | GT | Olink CARDIOVASCULAR II | -0,333131396 | 0,057872987 | 0,099146744 | ICU_vs_nonICU_radboud |
| OID00480 | LAP TGF-beta-1 | Olink INFLAMMATION | 0,197230548 | 0,058354873 | 0,099203285 | ICU_vs_nonICU_radboud |
| OID00414 | CTRC | Olink CARDIOVASCULAR II | -0,400969505 | 0,060316336 | 0,10175504 | ICU_vs_nonICU_radboud |
| OID00433 | XCL1 | Olink CARDIOVASCULAR II | 0,306044787 | 0,061260182 | 0,102564396 | ICU_vs_nonICU_radboud |
| OID01273 | NOTCH1 | Olink CARDIOMETABOLIC | -0,0868812 | 0,066286318 | 0,110144934 | ICU_vs_nonICU_radboud |
| OID01238 | MET | Olink CARDIOMETABOLIC | 0,09297943 | 0,07680065 | 0,126663758 | ICU_vs_nonICU_radboud |
| OID00457 | ACE2 | Olink CARDIOVASCULAR II | 0,330618279 | 0,079320904 | 0,129851258 | ICU_vs_nonICU_radboud |
| OID00469 | PARP-1 | Olink CARDIOVASCULAR II | 0,278890741 | 0,090698291 | 0,146855803 | ICU_vs_nonICU_radboud |
| OID00384 | PGF | Olink CARDIOVASCULAR II | 0,187612933 | 0,091037308 | 0,146855803 | ICU_vs_nonICU_radboud |
| OID01271 | COL18A1 | Olink CARDIOMETABOLIC | 0,150215312 | 0,092216273 | 0,147679684 | ICU_vs_nonICU_radboud |
| OID00446 | LPL | Olink CARDIOVASCULAR II | 0,145103873 | 0,103034541 | 0,163817507 | ICU_vs_nonICU_radboud |
| OID00418 | FS | Olink CARDIOVASCULAR II | -0,171755478 | 0,108041344 | 0,169675123 | ICU_vs_nonICU_radboud |
| OID01289 | PCOLCE | Olink CARDIOMETABOLIC | -0,143953036 | 0,108254264 | 0,169675123 | ICU_vs_nonICU_radboud |
| OID00398 | TIE2 | Olink CARDIOVASCULAR II | 0,082387703 | 0,125555601 | 0,195406957 | ICU_vs_nonICU_radboud |
| OID01291 | TGFBI | Olink CARDIOMETABOLIC | 0,137389665 | 0,128855877 | 0,1991409 | ICU_vs_nonICU_radboud |
| OID00445 | Dkk-1 | Olink CARDIOVASCULAR II | 0,209854445 | 0,133172096 | 0,203817433 | ICU_vs_nonICU_radboud |
| OID00425 | MERTK | Olink CARDIOVASCULAR II | -0,138486163 | 0,13372637 | 0,203817433 | ICU_vs_nonICU_radboud |
| OID00379 | BMP-6 | Olink CARDIOVASCULAR II | -0,133488779 | 0,13825164 | 0,209271318 | ICU_vs_nonICU_radboud |
| OID00406 | Gal-9 | Olink CARDIOVASCULAR II | 0,10783754 | 0,143118753 | 0,215164929 | ICU_vs_nonICU_radboud |
| OID01295 | VASN | Olink CARDIOMETABOLIC | 0,062369698 | 0,144946952 | 0,216441056 | ICU_vs_nonICU_radboud |
| OID00401 | PDGF subunit B | Olink CARDIOVASCULAR II | 0,335216303 | 0,148182652 | 0,219787691 | ICU_vs_nonICU_radboud |
| OID00382 | CD40-L | Olink CARDIOVASCULAR II | 0,356667827 | 0,169338976 | 0,248409977 | ICU_vs_nonICU_radboud |
| OID00427 | THBS2 | Olink CARDIOVASCULAR II | 0,068055299 | 0,169728084 | 0,248409977 | ICU_vs_nonICU_radboud |
| OID01248 | CD59 | Olink CARDIOMETABOLIC | 0,099815059 | 0,171998627 | 0,25007695 | ICU_vs_nonICU_radboud |
| OID00558 | STAMBP | Olink INFLAMMATION | -0,194443068 | 0,178226259 | 0,256953239 | ICU_vs_nonICU_radboud |
| OID00447 | PRSS8 | Olink CARDIOVASCULAR II | 0,105447437 | 0,180169196 | 0,256953239 | ICU_vs_nonICU_radboud |
| OID00438 | PSGL-1 | Olink CARDIOVASCULAR II | -0,077193887 | 0,180216072 | 0,256953239 | ICU_vs_nonICU_radboud |
| OID01276 | CCL18 | Olink CARDIOMETABOLIC | 0,187676706 | 0,183292763 | 0,259664747 | ICU_vs_nonICU_radboud |
| OID00404 | CXCL1 | Olink CARDIOVASCULAR II | 0,239655198 | 0,188524817 | 0,265375698 | ICU_vs_nonICU_radboud |
| OID00456 | MMP12 | Olink CARDIOVASCULAR II | 0,205383256 | 0,203401816 | 0,284223299 | ICU_vs_nonICU_radboud |
| OID00467 | NEMO | Olink CARDIOVASCULAR II | 0,258454297 | 0,204486446 | 0,284223299 | ICU_vs_nonICU_radboud |
| OID01219 | FCGR3B | Olink CARDIOMETABOLIC | 0,136864633 | 0,213652093 | 0,295106953 | ICU_vs_nonICU_radboud |
| OID01246 | CCL5 | Olink CARDIOMETABOLIC | 0,311234444 | 0,22714234 | 0,310191895 | ICU_vs_nonICU_radboud |
| OID01292 | CCL14 | Olink CARDIOMETABOLIC | 0,133257405 | 0,227380484 | 0,310191895 | ICU_vs_nonICU_radboud |
| OID00386 | BOC | Olink CARDIOVASCULAR II | -0,084640266 | 0,233308797 | 0,316326651 | ICU_vs_nonICU_radboud |
| OID00545 | FGF-19 | Olink INFLAMMATION | 0,229625835 | 0,240877617 | 0,324597277 | ICU_vs_nonICU_radboud |
| OID00560 | ADA | Olink INFLAMMATION | -0,132798781 | 0,250949828 | 0,336120679 | ICU_vs_nonICU_radboud |
| OID00460 | hOSCAR | Olink CARDIOVASCULAR II | 0,054635872 | 0,259516745 | 0,345501209 | ICU_vs_nonICU_radboud |
| OID00402 | IL-27 | Olink CARDIOVASCULAR II | 0,1346099 | 0,261831074 | 0,346495014 | ICU_vs_nonICU_radboud |
| OID00413 | SOD2 | Olink CARDIOVASCULAR II | -0,033062701 | 0,265651826 | 0,349458652 | ICU_vs_nonICU_radboud |
| OID00553 | TNFRSF9 | Olink INFLAMMATION | 0,149397964 | 0,299399734 | 0,391522729 | ICU_vs_nonICU_radboud |
| OID00392 | STK4 | Olink CARDIOVASCULAR II | 0,235303256 | 0,31504843 | 0,409562959 | ICU_vs_nonICU_radboud |
| OID01297 | LILRB1 | Olink CARDIOMETABOLIC | 0,079362743 | 0,323104535 | 0,417579545 | ICU_vs_nonICU_radboud |
| OID00442 | IgG Fc receptor II-b | Olink CARDIOVASCULAR II | 0,13184633 | 0,345455603 | 0,441596857 | ICU_vs_nonICU_radboud |
| OID05548 | TNF | Olink INFLAMMATION | 0,106602308 | 0,345684418 | 0,441596857 | ICU_vs_nonICU_radboud |
| OID00443 | ITGB1BP2 | Olink CARDIOVASCULAR II | 0,210339186 | 0,353282392 | 0,448709244 | ICU_vs_nonICU_radboud |
| OID00534 | CXCL6 | Olink INFLAMMATION | -0,144782932 | 0,404148984 | 0,509388128 | ICU_vs_nonICU_radboud |
| OID00411 | PIgR | Olink CARDIOVASCULAR II | 0,031490494 | 0,406840938 | 0,509388128 | ICU_vs_nonICU_radboud |
| OID01216 | CHL1 | Olink CARDIOMETABOLIC | -0,069238987 | 0,407971487 | 0,509388128 | ICU_vs_nonICU_radboud |
| OID01239 | F7 | Olink CARDIOMETABOLIC | 0,060725069 | 0,410342936 | 0,509470724 | ICU_vs_nonICU_radboud |
| OID00552 | CX3CL1 | Olink INFLAMMATION | -0,098316546 | 0,419143831 | 0,517490429 | ICU_vs_nonICU_radboud |
| OID01225 | CST3 | Olink CARDIOMETABOLIC | 0,097076221 | 0,441486994 | 0,54204792 | ICU_vs_nonICU_radboud |
| OID01255 | IGFBP3 | Olink CARDIOMETABOLIC | -0,083058442 | 0,454338122 | 0,552590893 | ICU_vs_nonICU_radboud |
| OID01233 | C2 | Olink CARDIOMETABOLIC | 0,052629257 | 0,455074853 | 0,552590893 | ICU_vs_nonICU_radboud |
| OID00435 | SORT1 | Olink CARDIOVASCULAR II | 0,05309204 | 0,458605907 | 0,553648635 | ICU_vs_nonICU_radboud |
| OID00407 | GIF | Olink CARDIOVASCULAR II | -0,178329166 | 0,460956329 | 0,553648635 | ICU_vs_nonICU_radboud |
| OID01220 | LILRB5 | Olink CARDIOMETABOLIC | -0,117167891 | 0,482072174 | 0,575880813 | ICU_vs_nonICU_radboud |
| OID05124 | CD8A | Olink INFLAMMATION | -0,106867218 | 0,50319196 | 0,597878619 | ICU_vs_nonICU_radboud |
| OID00452 | THPO | Olink CARDIOVASCULAR II | -0,087874759 | 0,520584486 | 0,61523621 | ICU_vs_nonICU_radboud |
| OID00523 | IL-12B | Olink INFLAMMATION | -0,108251024 | 0,540917483 | 0,634098288 | ICU_vs_nonICU_radboud |
| OID00470 | HAOX1 | Olink CARDIOVASCULAR II | 0,232719555 | 0,542283151 | 0,634098288 | ICU_vs_nonICU_radboud |
| OID00450 | GDF-2 | Olink CARDIOVASCULAR II | -0,079141377 | 0,548855598 | 0,638405722 | ICU_vs_nonICU_radboud |
| OID01251 | CD46 | Olink CARDIOMETABOLIC | 0,038380557 | 0,556884511 | 0,644353282 | ICU_vs_nonICU_radboud |
| OID00439 | CCL17 | Olink CARDIOVASCULAR II | 0,119377132 | 0,616009949 | 0,709053118 | ICU_vs_nonICU_radboud |
| OID01257 | VCAM1 | Olink CARDIOMETABOLIC | -0,045741505 | 0,630714291 | 0,716418639 | ICU_vs_nonICU_radboud |
| OID00395 | PAR-1 | Olink CARDIOVASCULAR II | 0,044242732 | 0,632048212 | 0,716418639 | ICU_vs_nonICU_radboud |
| OID00412 | RAGE | Olink CARDIOVASCULAR II | 0,064215568 | 0,632134093 | 0,716418639 | ICU_vs_nonICU_radboud |
| OID00449 | HB-EGF | Olink CARDIOVASCULAR II | 0,090950509 | 0,637794456 | 0,71914579 | ICU_vs_nonICU_radboud |
| OID00429 | VSIG2 | Olink CARDIOVASCULAR II | -0,062185054 | 0,656261084 | 0,736211673 | ICU_vs_nonICU_radboud |
| OID00505 | CCL11 | Olink INFLAMMATION | 0,049017968 | 0,679572931 | 0,75851322 | ICU_vs_nonICU_radboud |
| OID00380 | ANGPT1 | Olink CARDIOVASCULAR II | 0,087940701 | 0,691833011 | 0,768317062 | ICU_vs_nonICU_radboud |
| OID00511 | LIF-R | Olink INFLAMMATION | -0,03123567 | 0,701031196 | 0,773571235 | ICU_vs_nonICU_radboud |
| OID01296 | LILRB2 | Olink CARDIOMETABOLIC | 0,033712145 | 0,703564789 | 0,773571235 | ICU_vs_nonICU_radboud |
| OID00491 | CST5 | Olink INFLAMMATION | 0,035673107 | 0,750363311 | 0,820942038 | ICU_vs_nonICU_radboud |
| OID01256 | PAM | Olink CARDIOMETABOLIC | -0,016730044 | 0,76104848 | 0,828530611 | ICU_vs_nonICU_radboud |
| OID00420 | CD84 | Olink CARDIOVASCULAR II | -0,025415404 | 0,775647323 | 0,838848667 | ICU_vs_nonICU_radboud |
| OID00520 | CXCL5 | Olink INFLAMMATION | 0,087331448 | 0,778117542 | 0,838848667 | ICU_vs_nonICU_radboud |
| OID01286 | GAS6 | Olink CARDIOMETABOLIC | 0,019721419 | 0,792071844 | 0,849746979 | ICU_vs_nonICU_radboud |
| OID00478 | IL7 | Olink INFLAMMATION | -0,038705538 | 0,799018441 | 0,853058335 | ICU_vs_nonICU_radboud |
| OID00533 | Flt3L | Olink INFLAMMATION | 0,036965499 | 0,811937577 | 0,862683676 | ICU_vs_nonICU_radboud |
| OID01245 | CDH1 | Olink CARDIOMETABOLIC | 0,017151501 | 0,824157041 | 0,871477063 | ICU_vs_nonICU_radboud |
| OID00431 | PRELP | Olink CARDIOVASCULAR II | -0,011066718 | 0,848382736 | 0,892821832 | ICU_vs_nonICU_radboud |
| OID00550 | CASP-8 | Olink INFLAMMATION | 0,021076099 | 0,858271321 | 0,898947686 | ICU_vs_nonICU_radboud |
| OID00504 | MCP-4 | Olink INFLAMMATION | -0,027411317 | 0,874817666 | 0,911956152 | ICU_vs_nonICU_radboud |
| OID00458 | PD-L2 | Olink CARDIOVASCULAR II | -0,011922547 | 0,888281739 | 0,921644433 | ICU_vs_nonICU_radboud |
| OID00441 | MMP7 | Olink CARDIOVASCULAR II | 0,009698905 | 0,931429424 | 0,961896742 | ICU_vs_nonICU_radboud |
| OID00551 | CCL25 | Olink INFLAMMATION | -0,0106167 | 0,939922444 | 0,966152838 | ICU_vs_nonICU_radboud |
| OID00487 | AXIN1 | Olink INFLAMMATION | 0,014735783 | 0,944639064 | 0,966505709 | ICU_vs_nonICU_radboud |
| OID00432 | HO-1 | Olink CARDIOVASCULAR II | 0,004183183 | 0,961756378 | 0,978341836 | ICU_vs_nonICU_radboud |
| OID00393 | IDUA | Olink CARDIOVASCULAR II | 0,005422802 | 0,965061178 | 0,978341836 | ICU_vs_nonICU_radboud |
| OID00539 | CCL28 | Olink INFLAMMATION | 0,000392485 | 0,996509198 | 0,9977221 | ICU_vs_nonICU_radboud |
| OID05547 | IFN-gamma | Olink INFLAMMATION | -0,001140158 | 0,9977221 | 0,9977221 | ICU_vs_nonICU_radboud |
| OID01267 | ICAM3 | Olink CARDIOMETABOLIC | 0,00079632 | 0,991191215 | 0,9977221 | ICU_vs_nonICU_radboud |
| OID00441 | MMP7 | Olink CARDIOVASCULAR II | 5,83897363 | 3,72671E-49 | 8,19876E-47 | ICU_vs_healthy |
| OID01229 | SERPINA5 | Olink CARDIOMETABOLIC | -3,020932864 | 2,99521E-38 | 3,29473E-36 | ICU_vs_healthy |
| OID00562 | CSF-1 | Olink INFLAMMATION | 0,891025501 | 3,77411E-36 | 2,76768E-34 | ICU_vs_healthy |
| OID00518 | PD-L1 | Olink INFLAMMATION | 1,753537298 | 2,2042E-33 | 1,21231E-31 | ICU_vs_healthy |
| OID00408 | SCF | Olink CARDIOVASCULAR II | -2,388024811 | 1,30672E-30 | 5,74956E-29 | ICU_vs_healthy |
| OID00500 | SCF | Olink INFLAMMATION | -2,443705443 | 1,10812E-29 | 4,06312E-28 | ICU_vs_healthy |
| OID00437 | PTX3 | Olink CARDIOVASCULAR II | 2,080346144 | 2,92226E-29 | 9,18424E-28 | ICU_vs_healthy |
| OID00450 | GDF-2 | Olink CARDIOVASCULAR II | -2,366657822 | 2,44772E-28 | 6,73124E-27 | ICU_vs_healthy |
| OID00550 | CASP-8 | Olink INFLAMMATION | -4,054948485 | 6,98007E-28 | 1,70624E-26 | ICU_vs_healthy |
| OID00530 | CCL23 | Olink INFLAMMATION | 1,9456471 | 1,74614E-27 | 3,8415E-26 | ICU_vs_healthy |
| OID01274 | COMP | Olink CARDIOMETABOLIC | -1,777270817 | 3,278E-27 | 6,55599E-26 | ICU_vs_healthy |
| OID00521 | TRANCE | Olink INFLAMMATION | -2,386253661 | 2,65121E-26 | 4,86055E-25 | ICU_vs_healthy |
| OID00517 | IL-18R1 | Olink INFLAMMATION | 1,684750966 | 3,10467E-26 | 5,25406E-25 | ICU_vs_healthy |
| OID00466 | CD4 | Olink CARDIOVASCULAR II | 1,503086086 | 6,42351E-26 | 1,00941E-24 | ICU_vs_healthy |
| OID00391 | TNFRSF10A | Olink CARDIOVASCULAR II | 1,470840893 | 7,04152E-25 | 1,03276E-23 | ICU_vs_healthy |
| OID00406 | Gal-9 | Olink CARDIOVASCULAR II | 1,144773265 | 7,90769E-25 | 1,08731E-23 | ICU_vs_healthy |
| OID01250 | NID1 | Olink CARDIOMETABOLIC | 1,458668087 | 1,50207E-23 | 1,94385E-22 | ICU_vs_healthy |
| OID05548 | TNF | Olink INFLAMMATION | 2,272332577 | 8,40187E-23 | 1,02689E-21 | ICU_vs_healthy |
| OID01213 | DNER | Olink INFLAMMATION | -0,899672781 | 7,77761E-22 | 9,00566E-21 | ICU_vs_healthy |
| OID00459 | CTSL1 | Olink CARDIOVASCULAR II | 1,458541434 | 8,21947E-22 | 9,04142E-21 | ICU_vs_healthy |
| OID01224 | TIMP1 | Olink CARDIOMETABOLIC | 1,275121369 | 3,90709E-21 | 4,09314E-20 | ICU_vs_healthy |
| OID00386 | BOC | Olink CARDIOVASCULAR II | -1,00569707 | 4,99295E-21 | 4,99295E-20 | ICU_vs_healthy |
| OID00402 | IL-27 | Olink CARDIOVASCULAR II | 1,429954484 | 1,00835E-20 | 9,64505E-20 | ICU_vs_healthy |
| OID00522 | HGF | Olink INFLAMMATION | 2,184212518 | 2,47646E-20 | 2,27009E-19 | ICU_vs_healthy |
| OID01221 | APOM | Olink CARDIOMETABOLIC | -1,361516621 | 2,66963E-20 | 2,34927E-19 | ICU_vs_healthy |
| OID01252 | ST6GAL1 | Olink CARDIOMETABOLIC | 1,175228931 | 3,01552E-20 | 2,55159E-19 | ICU_vs_healthy |
| OID01302 | CFHR5 | Olink CARDIOMETABOLIC | 1,407476057 | 1,6251E-19 | 1,32415E-18 | ICU_vs_healthy |
| OID00486 | CXCL11 | Olink INFLAMMATION | 2,589647694 | 2,97734E-19 | 2,33934E-18 | ICU_vs_healthy |
| OID00381 | ADM | Olink CARDIOVASCULAR II | 3,846372316 | 7,61212E-19 | 5,77471E-18 | ICU_vs_healthy |
| OID00535 | CXCL10 | Olink INFLAMMATION | 2,55284045 | 3,24165E-18 | 2,33901E-17 | ICU_vs_healthy |
| OID01266 | DPP4 | Olink CARDIOMETABOLIC | -0,961863259 | 3,29588E-18 | 2,33901E-17 | ICU_vs_healthy |
| OID00448 | AGRP | Olink CARDIOVASCULAR II | 1,761289986 | 4,57854E-18 | 3,14775E-17 | ICU_vs_healthy |
| OID00476 | CDCP1 | Olink INFLAMMATION | 1,432824121 | 1,22218E-17 | 8,14784E-17 | ICU_vs_healthy |
| OID01258 | CR2 | Olink CARDIOMETABOLIC | -1,425686983 | 1,39456E-17 | 9,02364E-17 | ICU_vs_healthy |
| OID00513 | CCL19 | Olink INFLAMMATION | 2,972810635 | 2,19129E-17 | 1,37738E-16 | ICU_vs_healthy |
| OID05547 | IFN-gamma | Olink INFLAMMATION | 3,687509408 | 2,42964E-17 | 1,48478E-16 | ICU_vs_healthy |
| OID00457 | ACE2 | Olink CARDIOVASCULAR II | 1,797822862 | 3,04722E-17 | 1,81186E-16 | ICU_vs_healthy |
| OID00542 | CD40 | Olink INFLAMMATION | 1,223941582 | 5,5187E-17 | 3,19504E-16 | ICU_vs_healthy |
| OID01241 | KIT | Olink CARDIOMETABOLIC | -0,857202074 | 7,03269E-17 | 3,93596E-16 | ICU_vs_healthy |
| OID05124 | CD8A | Olink INFLAMMATION | 2,41860523 | 7,15629E-17 | 3,93596E-16 | ICU_vs_healthy |
| OID00432 | HO-1 | Olink CARDIOVASCULAR II | 1,09208602 | 8,30932E-17 | 4,45866E-16 | ICU_vs_healthy |
| OID01289 | PCOLCE | Olink CARDIOMETABOLIC | -0,950744657 | 1,51019E-16 | 7,9105E-16 | ICU_vs_healthy |
| OID01265 | TNC | Olink CARDIOMETABOLIC | 1,973472299 | 2,3474E-16 | 1,201E-15 | ICU_vs_healthy |
| OID00425 | MERTK | Olink CARDIOVASCULAR II | 1,013895504 | 2,84736E-16 | 1,40199E-15 | ICU_vs_healthy |
| OID00556 | CCL20 | Olink INFLAMMATION | 2,787786688 | 2,86772E-16 | 1,40199E-15 | ICU_vs_healthy |
| OID00479 | OPG | Olink INFLAMMATION | 1,13190183 | 3,17263E-16 | 1,51734E-15 | ICU_vs_healthy |
| OID00472 | VEGFA | Olink INFLAMMATION | 1,465664325 | 3,26176E-16 | 1,52678E-15 | ICU_vs_healthy |
| OID00419 | GLO1 | Olink CARDIOVASCULAR II | 2,353492415 | 5,21075E-16 | 2,38826E-15 | ICU_vs_healthy |
| OID01286 | GAS6 | Olink CARDIOMETABOLIC | 0,843974386 | 6,51257E-16 | 2,92401E-15 | ICU_vs_healthy |
| OID00484 | MCP-1 | Olink INFLAMMATION | 1,834327857 | 7,32964E-16 | 3,22504E-15 | ICU_vs_healthy |
| OID00397 | PRSS27 | Olink CARDIOVASCULAR II | -1,224639055 | 1,07285E-15 | 4,62799E-15 | ICU_vs_healthy |
| OID00426 | KIM1 | Olink CARDIOVASCULAR II | 1,756495969 | 1,81222E-15 | 7,66708E-15 | ICU_vs_healthy |
| OID00433 | XCL1 | Olink CARDIOVASCULAR II | 1,910733367 | 2,58293E-15 | 1,07216E-14 | ICU_vs_healthy |
| OID00396 | TRAIL-R2 | Olink CARDIOVASCULAR II | 1,560151387 | 2,98466E-15 | 1,21597E-14 | ICU_vs_healthy |
| OID00416 | SPON2 | Olink CARDIOVASCULAR II | 0,486093465 | 1,28162E-14 | 5,12649E-14 | ICU_vs_healthy |
| OID01240 | IGLC2 | Olink CARDIOMETABOLIC | 1,172449911 | 4,52203E-14 | 1,77651E-13 | ICU_vs_healthy |
| OID00499 | CD6 | Olink INFLAMMATION | -1,235223722 | 4,91875E-14 | 1,89847E-13 | ICU_vs_healthy |
| OID00532 | CCL3 | Olink INFLAMMATION | 1,384031407 | 1,74525E-13 | 6,61992E-13 | ICU_vs_healthy |
| OID00512 | FGF-21 | Olink INFLAMMATION | 3,291587094 | 2,18702E-13 | 8,155E-13 | ICU_vs_healthy |
| OID00431 | PRELP | Olink CARDIOVASCULAR II | 0,541410945 | 3,38946E-13 | 1,2428E-12 | ICU_vs_healthy |
| OID00552 | CX3CL1 | Olink INFLAMMATION | 0,915491331 | 6,6377E-13 | 2,39392E-12 | ICU_vs_healthy |
| OID01259 | TCN2 | Olink CARDIOMETABOLIC | 0,919902682 | 6,84139E-13 | 2,42759E-12 | ICU_vs_healthy |
| OID00410 | FGF-21 | Olink CARDIOVASCULAR II | 3,370605744 | 7,01371E-13 | 2,44923E-12 | ICU_vs_healthy |
| OID00481 | uPA | Olink INFLAMMATION | 0,864216911 | 8,93222E-13 | 3,07045E-12 | ICU_vs_healthy |
| OID01253 | IL7R | Olink CARDIOMETABOLIC | -1,105375408 | 1,18368E-12 | 4,00631E-12 | ICU_vs_healthy |
| OID00379 | BMP-6 | Olink CARDIOVASCULAR II | 2,068564561 | 1,42365E-12 | 4,74549E-12 | ICU_vs_healthy |
| OID01284 | PTPRS | Olink CARDIOMETABOLIC | -0,413679453 | 2,60208E-12 | 8,54416E-12 | ICU_vs_healthy |
| OID01268 | THBS4 | Olink CARDIOMETABOLIC | -1,177175877 | 5,6221E-12 | 1,81892E-11 | ICU_vs_healthy |
| OID00393 | IDUA | Olink CARDIOVASCULAR II | 2,184831081 | 8,17858E-12 | 2,60766E-11 | ICU_vs_healthy |
| OID01299 | CNDP1 | Olink CARDIOMETABOLIC | 0,940093414 | 4,21865E-11 | 1,32586E-10 | ICU_vs_healthy |
| OID00423 | REN | Olink CARDIOVASCULAR II | 1,267006093 | 5,98091E-11 | 1,85324E-10 | ICU_vs_healthy |
| OID01297 | LILRB1 | Olink CARDIOMETABOLIC | 0,624973778 | 8,39112E-11 | 2,56395E-10 | ICU_vs_healthy |
| OID01281 | EFEMP1 | Olink CARDIOMETABOLIC | 1,206683315 | 2,74825E-10 | 8,2824E-10 | ICU_vs_healthy |
| OID00549 | MCP-2 | Olink INFLAMMATION | 1,690098751 | 3,96832E-10 | 1,17977E-09 | ICU_vs_healthy |
| OID01294 | AOC3 | Olink CARDIOMETABOLIC | -0,476182725 | 4,21267E-10 | 1,23572E-09 | ICU_vs_healthy |
| OID00398 | TIE2 | Olink CARDIOVASCULAR II | -0,444484021 | 7,31092E-10 | 2,11632E-09 | ICU_vs_healthy |
| OID01254 | ENG | Olink CARDIOMETABOLIC | -0,391569157 | 7,86262E-10 | 2,24646E-09 | ICU_vs_healthy |
| OID01296 | LILRB2 | Olink CARDIOMETABOLIC | 0,654266253 | 1,84745E-09 | 5,21076E-09 | ICU_vs_healthy |
| OID00460 | hOSCAR | Olink CARDIOVASCULAR II | 0,380777501 | 1,9911E-09 | 5,54483E-09 | ICU_vs_healthy |
| OID01257 | VCAM1 | Olink CARDIOMETABOLIC | 0,626877462 | 2,7794E-09 | 7,64335E-09 | ICU_vs_healthy |
| OID01230 | ICAM1 | Olink CARDIOMETABOLIC | 0,642677512 | 2,97951E-09 | 8,09249E-09 | ICU_vs_healthy |
| OID00443 | ITGB1BP2 | Olink CARDIOVASCULAR II | 1,509760685 | 6,09377E-09 | 1,63491E-08 | ICU_vs_healthy |
| OID00389 | IL-1ra | Olink CARDIOVASCULAR II | 1,424030579 | 8,88667E-09 | 2,3555E-08 | ICU_vs_healthy |
| OID00453 | MARCO | Olink CARDIOVASCULAR II | 0,730987505 | 1,50435E-08 | 3,93997E-08 | ICU_vs_healthy |
| OID00427 | THBS2 | Olink CARDIOVASCULAR II | 0,339466455 | 1,8579E-08 | 4,80867E-08 | ICU_vs_healthy |
| OID01220 | LILRB5 | Olink CARDIOMETABOLIC | -1,074673201 | 2,01445E-08 | 5,15324E-08 | ICU_vs_healthy |
| OID00384 | PGF | Olink CARDIOVASCULAR II | 0,494096223 | 2,36579E-08 | 5,98247E-08 | ICU_vs_healthy |
| OID00440 | CCL3 | Olink CARDIOVASCULAR II | 1,147344885 | 2,6663E-08 | 6,66575E-08 | ICU_vs_healthy |
| OID00385 | ADAM-TS13 | Olink CARDIOVASCULAR II | -0,312651378 | 3,66595E-08 | 9,06189E-08 | ICU_vs_healthy |
| OID00444 | DCN | Olink CARDIOVASCULAR II | 0,548182601 | 4,16368E-08 | 1,01779E-07 | ICU_vs_healthy |
| OID00490 | CXCL9 | Olink INFLAMMATION | 1,444890782 | 6,59076E-08 | 1,59337E-07 | ICU_vs_healthy |
| OID01255 | IGFBP3 | Olink CARDIOMETABOLIC | -0,699725777 | 7,93377E-08 | 1,89721E-07 | ICU_vs_healthy |
| OID00394 | TNFRSF11A | Olink CARDIOVASCULAR II | 0,720697296 | 1,78697E-07 | 4,22724E-07 | ICU_vs_healthy |
| OID01247 | NCAM1 | Olink CARDIOMETABOLIC | -0,377569122 | 1,87601E-07 | 4,39066E-07 | ICU_vs_healthy |
| OID01301 | C1QTNF1 | Olink CARDIOMETABOLIC | 0,73109337 | 3,91625E-07 | 9,06922E-07 | ICU_vs_healthy |
| OID00401 | PDGF subunit B | Olink CARDIOVASCULAR II | -0,647276887 | 4,06037E-07 | 9,30502E-07 | ICU_vs_healthy |
| OID00439 | CCL17 | Olink CARDIOVASCULAR II | -1,47244189 | 6,47333E-07 | 1,45463E-06 | ICU_vs_healthy |
| OID00470 | HAOX1 | Olink CARDIOVASCULAR II | 2,078860267 | 6,4797E-07 | 1,45463E-06 | ICU_vs_healthy |
| OID01218 | PLXNB2 | Olink CARDIOMETABOLIC | 0,359386735 | 9,64046E-07 | 2,14232E-06 | ICU_vs_healthy |
| OID01293 | QPCT | Olink CARDIOMETABOLIC | 0,383579148 | 1,00111E-06 | 2,20245E-06 | ICU_vs_healthy |
| OID00438 | PSGL-1 | Olink CARDIOVASCULAR II | -0,323233189 | 1,95812E-06 | 4,26521E-06 | ICU_vs_healthy |
| OID00536 | 4E-BP1 | Olink INFLAMMATION | 1,871034355 | 2,88804E-06 | 6,2291E-06 | ICU_vs_healthy |
| OID00417 | GH | Olink CARDIOVASCULAR II | 2,29823408 | 3,72413E-06 | 7,95445E-06 | ICU_vs_healthy |
| OID00510 | MMP-1 | Olink INFLAMMATION | 2,113020631 | 4,01387E-06 | 8,49088E-06 | ICU_vs_healthy |
| OID00446 | LPL | Olink CARDIOVASCULAR II | 0,742557666 | 4,11758E-06 | 8,62731E-06 | ICU_vs_healthy |
| OID00412 | RAGE | Olink CARDIOVASCULAR II | 0,750283707 | 5,01477E-06 | 1,0408E-05 | ICU_vs_healthy |
| OID01243 | MBL2 | Olink CARDIOMETABOLIC | 1,281351361 | 5,35129E-06 | 1,10027E-05 | ICU_vs_healthy |
| OID01264 | IGFBP6 | Olink CARDIOMETABOLIC | -0,455686519 | 5,89463E-06 | 1,20076E-05 | ICU_vs_healthy |
| OID00503 | TGF-alpha | Olink INFLAMMATION | 0,819811097 | 8,59105E-06 | 1,73397E-05 | ICU_vs_healthy |
| OID00523 | IL-12B | Olink INFLAMMATION | 0,780532502 | 9,13274E-06 | 1,82655E-05 | ICU_vs_healthy |
| OID01298 | TIMD4 | Olink CARDIOMETABOLIC | 0,792467975 | 9,51629E-06 | 1,88611E-05 | ICU_vs_healthy |
| OID00506 | TNFSF14 | Olink INFLAMMATION | 0,991389411 | 9,74863E-06 | 1,91491E-05 | ICU_vs_healthy |
| OID01236 | PRSS2 | Olink CARDIOMETABOLIC | 1,156185792 | 1,12137E-05 | 2,1832E-05 | ICU_vs_healthy |
| OID01305 | FETUB | Olink CARDIOMETABOLIC | -0,632576357 | 1,17818E-05 | 2,27369E-05 | ICU_vs_healthy |
| OID01262 | GNLY | Olink CARDIOMETABOLIC | 0,496635041 | 1,46662E-05 | 2,80572E-05 | ICU_vs_healthy |
| OID01269 | SAA4 | Olink CARDIOMETABOLIC | -1,033122883 | 1,7371E-05 | 3,2945E-05 | ICU_vs_healthy |
| OID00487 | AXIN1 | Olink INFLAMMATION | 1,281383238 | 1,97296E-05 | 3,70984E-05 | ICU_vs_healthy |
| OID01217 | NRP1 | Olink CARDIOMETABOLIC | 0,234273668 | 2,10932E-05 | 3,93262E-05 | ICU_vs_healthy |
| OID00411 | PIgR | Olink CARDIOVASCULAR II | 0,189183206 | 2,67946E-05 | 4,95362E-05 | ICU_vs_healthy |
| OID00407 | GIF | Olink CARDIOVASCULAR II | -1,121256717 | 2,98531E-05 | 5,47306E-05 | ICU_vs_healthy |
| OID00471 | IL8 | Olink INFLAMMATION | 1,470952044 | 4,52733E-05 | 8,2315E-05 | ICU_vs_healthy |
| OID01216 | CHL1 | Olink CARDIOMETABOLIC | -0,371721965 | 5,98063E-05 | 0,000107847 | ICU_vs_healthy |
| OID01231 | REG1A | Olink CARDIOMETABOLIC | 0,889677546 | 0,000135572 | 0,000242486 | ICU_vs_healthy |
| OID01291 | TGFBI | Olink CARDIOMETABOLIC | 0,450444051 | 0,000144036 | 0,000255548 | ICU_vs_healthy |
| OID00418 | FS | Olink CARDIOVASCULAR II | 0,561369917 | 0,000147798 | 0,000260124 | ICU_vs_healthy |
| OID00555 | TWEAK | Olink INFLAMMATION | 0,78403541 | 0,000226633 | 0,000395708 | ICU_vs_healthy |
| OID01290 | FCN2 | Olink CARDIOMETABOLIC | 0,440667509 | 0,000275731 | 0,000477644 | ICU_vs_healthy |
| OID00560 | ADA | Olink INFLAMMATION | 0,5817 | 0,000385419 | 0,00066244 | ICU_vs_healthy |
| OID01228 | PROC | Olink CARDIOMETABOLIC | -0,374583393 | 0,000410444 | 0,000699982 | ICU_vs_healthy |
| OID01306 | ANGPTL3 | Olink CARDIOMETABOLIC | 0,416426108 | 0,000441593 | 0,000747311 | ICU_vs_healthy |
| OID00468 | VEGFD | Olink CARDIOVASCULAR II | -0,498432098 | 0,00051821 | 0,000870277 | ICU_vs_healthy |
| OID00504 | MCP-4 | Olink INFLAMMATION | -0,764261266 | 0,00063364 | 0,001056067 | ICU_vs_healthy |
| OID00498 | CCL4 | Olink INFLAMMATION | 0,574298535 | 0,001084805 | 0,001794415 | ICU_vs_healthy |
| OID00382 | CD40-L | Olink CARDIOVASCULAR II | 1,101076735 | 0,001093408 | 0,001795147 | ICU_vs_healthy |
| OID00561 | TNFB | Olink INFLAMMATION | -0,399819188 | 0,001124274 | 0,00183215 | ICU_vs_healthy |
| OID00558 | STAMBP | Olink INFLAMMATION | 0,783027128 | 0,001279757 | 0,002070195 | ICU_vs_healthy |
| OID00477 | CD244 | Olink INFLAMMATION | -0,300138767 | 0,001568104 | 0,002518123 | ICU_vs_healthy |
| OID01245 | CDH1 | Olink CARDIOMETABOLIC | -0,253420787 | 0,001781316 | 0,002839779 | ICU_vs_healthy |
| OID00527 | MMP-10 | Olink INFLAMMATION | 0,659783596 | 0,001799946 | 0,002848835 | ICU_vs_healthy |
| OID00435 | SORT1 | Olink CARDIOVASCULAR II | -0,247118989 | 0,002182976 | 0,003430391 | ICU_vs_healthy |
| OID00539 | CCL28 | Olink INFLAMMATION | -0,39804318 | 0,002403428 | 0,003750029 | ICU_vs_healthy |
| OID01235 | CA3 | Olink CARDIOMETABOLIC | 0,534988104 | 0,002690931 | 0,004169048 | ICU_vs_healthy |
| OID01273 | NOTCH1 | Olink CARDIOMETABOLIC | -0,159899118 | 0,002903333 | 0,004466666 | ICU_vs_healthy |
| OID00488 | TRAIL | Olink INFLAMMATION | -0,325419596 | 0,003312358 | 0,005060548 | ICU_vs_healthy |
| OID00531 | CD5 | Olink INFLAMMATION | -0,29510771 | 0,003488727 | 0,005293241 | ICU_vs_healthy |
| OID00409 | IL18 | Olink CARDIOVASCULAR II | 0,846388996 | 0,004665573 | 0,007030315 | ICU_vs_healthy |
| OID00494 | OSM | Olink INFLAMMATION | 0,789837653 | 0,005168035 | 0,007734474 | ICU_vs_healthy |
| OID01238 | MET | Olink CARDIOMETABOLIC | 0,194447433 | 0,005630571 | 0,008369768 | ICU_vs_healthy |
| OID00454 | GT | Olink CARDIOVASCULAR II | 0,553325677 | 0,00677073 | 0,00999705 | ICU_vs_healthy |
| OID00434 | IL16 | Olink CARDIOVASCULAR II | -0,565296357 | 0,007087623 | 0,01039518 | ICU_vs_healthy |
| OID00533 | Flt3L | Olink INFLAMMATION | -0,386387059 | 0,007331707 | 0,010640763 | ICU_vs_healthy |
| OID01225 | CST3 | Olink CARDIOMETABOLIC | 0,337760358 | 0,0073518 | 0,010640763 | ICU_vs_healthy |
| OID00491 | CST5 | Olink INFLAMMATION | -0,309669004 | 0,009590337 | 0,013790028 | ICU_vs_healthy |
| OID00449 | HB-EGF | Olink CARDIOVASCULAR II | -0,57659117 | 0,010365043 | 0,014807204 | ICU_vs_healthy |
| OID00392 | STK4 | Olink CARDIOVASCULAR II | 0,769219064 | 0,011250542 | 0,015968511 | ICU_vs_healthy |
| OID00451 | FABP2 | Olink CARDIOVASCULAR II | -0,687745481 | 0,012657564 | 0,017850411 | ICU_vs_healthy |
| OID00467 | NEMO | Olink CARDIOVASCULAR II | 0,711358226 | 0,012831841 | 0,017980924 | ICU_vs_healthy |
| OID01271 | COL18A1 | Olink CARDIOMETABOLIC | 0,258710199 | 0,01396198 | 0,019440732 | ICU_vs_healthy |
| OID00414 | CTRC | Olink CARDIOVASCULAR II | -0,583705065 | 0,014242332 | 0,019706371 | ICU_vs_healthy |
| OID00456 | MMP12 | Olink CARDIOVASCULAR II | 0,518839118 | 0,016376546 | 0,02251775 | ICU_vs_healthy |
| OID00462 | TGM2 | Olink CARDIOVASCULAR II | -0,479714628 | 0,016922718 | 0,02312421 | ICU_vs_healthy |
| OID00501 | IL18 | Olink INFLAMMATION | 0,704185946 | 0,017351011 | 0,023563101 | ICU_vs_healthy |
| OID01233 | C2 | Olink CARDIOMETABOLIC | -0,353082294 | 0,019379506 | 0,026156389 | ICU_vs_healthy |
| OID00445 | Dkk-1 | Olink CARDIOVASCULAR II | -0,332477802 | 0,024816643 | 0,033290618 | ICU_vs_healthy |
| OID00400 | IL1RL2 | Olink CARDIOVASCULAR II | 0,229887497 | 0,027725514 | 0,036967353 | ICU_vs_healthy |
| OID00452 | THPO | Olink CARDIOVASCULAR II | 0,301987535 | 0,032151228 | 0,042610062 | ICU_vs_healthy |
| OID00520 | CXCL5 | Olink INFLAMMATION | -0,697371274 | 0,032581041 | 0,042921132 | ICU_vs_healthy |
| OID00422 | SERPINA12 | Olink CARDIOVASCULAR II | -0,578667218 | 0,042454741 | 0,055595494 | ICU_vs_healthy |
| OID00380 | ANGPT1 | Olink CARDIOVASCULAR II | -0,448403434 | 0,043652106 | 0,056825226 | ICU_vs_healthy |
| OID01256 | PAM | Olink CARDIOMETABOLIC | 0,164685605 | 0,049963149 | 0,064658193 | ICU_vs_healthy |
| OID00511 | LIF-R | Olink INFLAMMATION | 0,156436337 | 0,054909824 | 0,070644218 | ICU_vs_healthy |
| OID00442 | IgG Fc receptor II-b | Olink CARDIOVASCULAR II | 0,325958279 | 0,064605106 | 0,082634438 | ICU_vs_healthy |
| OID00478 | IL7 | Olink INFLAMMATION | 0,312307511 | 0,065208794 | 0,08272815 | ICU_vs_healthy |
| OID00405 | LOX-1 | Olink CARDIOVASCULAR II | -0,453580938 | 0,065430446 | 0,08272815 | ICU_vs_healthy |
| OID00545 | FGF-19 | Olink INFLAMMATION | -0,433018517 | 0,069295547 | 0,087114402 | ICU_vs_healthy |
| OID00461 | TNFRSF13B | Olink CARDIOVASCULAR II | 0,280320174 | 0,069766032 | 0,08720754 | ICU_vs_healthy |
| OID01226 | ANG | Olink CARDIOMETABOLIC | 0,17669212 | 0,073640057 | 0,091530014 | ICU_vs_healthy |
| OID00399 | TF | Olink CARDIOVASCULAR II | 0,156429309 | 0,081722835 | 0,101005751 | ICU_vs_healthy |
| OID01246 | CCL5 | Olink CARDIOMETABOLIC | -0,403423141 | 0,092874482 | 0,114147408 | ICU_vs_healthy |
| OID00551 | CCL25 | Olink INFLAMMATION | 0,228272503 | 0,1005311 | 0,122871344 | ICU_vs_healthy |
| OID01251 | CD46 | Olink CARDIOMETABOLIC | -0,145137167 | 0,102882379 | 0,125050406 | ICU_vs_healthy |
| OID01232 | SERPINA7 | Olink CARDIOMETABOLIC | -0,169341747 | 0,105578189 | 0,126609281 | ICU_vs_healthy |
| OID00496 | CXCL1 | Olink INFLAMMATION | 0,3670692 | 0,105718194 | 0,126609281 | ICU_vs_healthy |
| OID00553 | TNFRSF9 | Olink INFLAMMATION | 0,214455176 | 0,105891398 | 0,126609281 | ICU_vs_healthy |
| OID01300 | OSMR | Olink CARDIOMETABOLIC | 0,092973845 | 0,128684419 | 0,15303012 | ICU_vs_healthy |
| OID00463 | LEP | Olink CARDIOVASCULAR II | -0,455963182 | 0,141342621 | 0,167179445 | ICU_vs_healthy |
| OID01307 | LYVE1 | Olink CARDIOMETABOLIC | -0,170285478 | 0,143086152 | 0,16833665 | ICU_vs_healthy |
| OID00388 | SRC | Olink CARDIOVASCULAR II | 0,256719411 | 0,161039399 | 0,18845036 | ICU_vs_healthy |
| OID01276 | CCL18 | Olink CARDIOMETABOLIC | 0,269998321 | 0,167129531 | 0,194542311 | ICU_vs_healthy |
| OID00465 | HSP 27 | Olink CARDIOVASCULAR II | -0,131069876 | 0,194286268 | 0,224963048 | ICU_vs_healthy |
| OID00430 | AMBP | Olink CARDIOVASCULAR II | 0,073247641 | 0,20127891 | 0,231839582 | ICU_vs_healthy |
| OID01249 | SELL | Olink CARDIOMETABOLIC | -0,097160613 | 0,210106549 | 0,240747088 | ICU_vs_healthy |
| OID01239 | F7 | Olink CARDIOMETABOLIC | 0,107022892 | 0,22164581 | 0,252653254 | ICU_vs_healthy |
| OID00458 | PD-L2 | Olink CARDIOVASCULAR II | -0,109651638 | 0,226777234 | 0,257170059 | ICU_vs_healthy |
| OID00436 | CEACAM8 | Olink CARDIOVASCULAR II | -0,270358952 | 0,250259382 | 0,282343918 | ICU_vs_healthy |
| OID00447 | PRSS8 | Olink CARDIOVASCULAR II | 0,103865273 | 0,267996697 | 0,300812619 | ICU_vs_healthy |
| OID01292 | CCL14 | Olink CARDIOMETABOLIC | 0,135943741 | 0,275094634 | 0,307212282 | ICU_vs_healthy |
| OID00420 | CD84 | Olink CARDIOVASCULAR II | -0,134549048 | 0,277388068 | 0,308208964 | ICU_vs_healthy |
| OID00480 | LAP TGF-beta-1 | Olink INFLAMMATION | -0,177780094 | 0,2791741 | 0,308634683 | ICU_vs_healthy |
| OID01223 | CA1 | Olink CARDIOMETABOLIC | -0,25335152 | 0,304824959 | 0,335307455 | ICU_vs_healthy |
| OID01248 | CD59 | Olink CARDIOMETABOLIC | 0,077488648 | 0,325899652 | 0,356706086 | ICU_vs_healthy |
| OID01270 | TIE1 | Olink CARDIOMETABOLIC | -0,065551785 | 0,344866331 | 0,375596994 | ICU_vs_healthy |
| OID00541 | EN-RAGE | Olink INFLAMMATION | 0,215897984 | 0,349127107 | 0,378364352 | ICU_vs_healthy |
| OID00404 | CXCL1 | Olink CARDIOVASCULAR II | 0,211941753 | 0,366820917 | 0,395579757 | ICU_vs_healthy |
| OID01267 | ICAM3 | Olink CARDIOMETABOLIC | -0,062928297 | 0,36860841 | 0,395579757 | ICU_vs_healthy |
| OID00505 | CCL11 | Olink INFLAMMATION | -0,116536721 | 0,456058998 | 0,487053298 | ICU_vs_healthy |
| OID01303 | MEGF9 | Olink CARDIOMETABOLIC | 0,04156835 | 0,600910888 | 0,638649253 | ICU_vs_healthy |
| OID01295 | VASN | Olink CARDIOMETABOLIC | 0,024994852 | 0,635468713 | 0,67213037 | ICU_vs_healthy |
| OID00515 | IL-10RB | Olink INFLAMMATION | 0,032596386 | 0,662224128 | 0,697078029 | ICU_vs_healthy |
| OID00534 | CXCL6 | Olink INFLAMMATION | 0,095995669 | 0,682687395 | 0,715196319 | ICU_vs_healthy |
| OID01234 | GP1BA | Olink CARDIOMETABOLIC | -0,047322076 | 0,69862596 | 0,728425172 | ICU_vs_healthy |
| OID00469 | PARP-1 | Olink CARDIOVASCULAR II | -0,114285885 | 0,758345328 | 0,786962133 | ICU_vs_healthy |
| OID00428 | TM | Olink CARDIOVASCULAR II | 0,022622803 | 0,775921466 | 0,801421232 | ICU_vs_healthy |
| OID01227 | F11 | Olink CARDIOMETABOLIC | -0,022424126 | 0,788362293 | 0,810465908 | ICU_vs_healthy |
| OID00413 | SOD2 | Olink CARDIOVASCULAR II | -0,011721223 | 0,798571238 | 0,817142662 | ICU_vs_healthy |
| OID00395 | PAR-1 | Olink CARDIOVASCULAR II | -0,017585991 | 0,862355417 | 0,878324962 | ICU_vs_healthy |
| OID01219 | FCGR3B | Olink CARDIOMETABOLIC | -0,016188492 | 0,867765407 | 0,879762164 | ICU_vs_healthy |
| OID01287 | SPARCL1 | Olink CARDIOMETABOLIC | 0,009622091 | 0,899822877 | 0,908078133 | ICU_vs_healthy |
| OID01244 | FCGR2A | Olink CARDIOMETABOLIC | -0,007783683 | 0,943570964 | 0,947879507 | ICU_vs_healthy |
| OID00429 | VSIG2 | Olink CARDIOVASCULAR II | 0,000337543 | 0,998062032 | 0,998062032 | ICU_vs_healthy |
| OID00441 | MMP7 | Olink CARDIOVASCULAR II | 5,766479521 | 9,68626E-89 | 2,13098E-86 | nonICU_vs_healthy |
| OID00562 | CSF-1 | Olink INFLAMMATION | 0,804958036 | 1,63834E-55 | 1,80217E-53 | nonICU_vs_healthy |
| OID00550 | CASP-8 | Olink INFLAMMATION | -3,831325825 | 3,18529E-51 | 2,33588E-49 | nonICU_vs_healthy |
| OID01229 | SERPINA5 | Olink CARDIOMETABOLIC | -2,360095725 | 6,36865E-48 | 3,50276E-46 | nonICU_vs_healthy |
| OID00466 | CD4 | Olink CARDIOVASCULAR II | 1,251031462 | 2,97825E-41 | 1,31043E-39 | nonICU_vs_healthy |
| OID00450 | GDF-2 | Olink CARDIOVASCULAR II | -2,202103654 | 3,22292E-39 | 1,18174E-37 | nonICU_vs_healthy |
| OID05548 | TNF | Olink INFLAMMATION | 2,08978346 | 2,25759E-37 | 7,09527E-36 | nonICU_vs_healthy |
| OID00381 | ADM | Olink CARDIOVASCULAR II | 3,424124632 | 2,75192E-37 | 7,56779E-36 | nonICU_vs_healthy |
| OID00437 | PTX3 | Olink CARDIOVASCULAR II | 1,725610022 | 1,93507E-34 | 4,73017E-33 | nonICU_vs_healthy |
| OID00521 | TRANCE | Olink INFLAMMATION | -1,80510841 | 3,15072E-34 | 6,93158E-33 | nonICU_vs_healthy |
| OID00518 | PD-L1 | Olink INFLAMMATION | 1,332241875 | 1,69979E-33 | 3,39958E-32 | nonICU_vs_healthy |
| OID00419 | GLO1 | Olink CARDIOVASCULAR II | 2,681197545 | 2,56435E-33 | 4,70131E-32 | nonICU_vs_healthy |
| OID05124 | CD8A | Olink INFLAMMATION | 2,451308928 | 1,41099E-32 | 2,38782E-31 | nonICU_vs_healthy |
| OID01274 | COMP | Olink CARDIOMETABOLIC | -1,464187402 | 1,72048E-31 | 2,70361E-30 | nonICU_vs_healthy |
| OID00406 | Gal-9 | Olink CARDIOVASCULAR II | 1,048771464 | 1,37102E-30 | 2,01084E-29 | nonICU_vs_healthy |
| OID00517 | IL-18R1 | Olink INFLAMMATION | 1,212971354 | 4,72232E-30 | 6,49319E-29 | nonICU_vs_healthy |
| OID00530 | CCL23 | Olink INFLAMMATION | 1,405482614 | 5,36042E-30 | 6,93702E-29 | nonICU_vs_healthy |
| OID00425 | MERTK | Olink CARDIOVASCULAR II | 1,172260931 | 4,0627E-29 | 4,96553E-28 | nonICU_vs_healthy |
| OID00386 | BOC | Olink CARDIOVASCULAR II | -0,881610515 | 2,45891E-28 | 2,84715E-27 | nonICU_vs_healthy |
| OID00432 | HO-1 | Olink CARDIOVASCULAR II | 1,161992663 | 1,94952E-27 | 2,14447E-26 | nonICU_vs_healthy |
| OID00379 | BMP-6 | Olink CARDIOVASCULAR II | 2,094591781 | 2,15846E-27 | 2,26124E-26 | nonICU_vs_healthy |
| OID01250 | NID1 | Olink CARDIOMETABOLIC | 1,055271157 | 7,85668E-27 | 7,85668E-26 | nonICU_vs_healthy |
| OID00402 | IL-27 | Olink CARDIOVASCULAR II | 1,341584147 | 1,4342E-26 | 1,37184E-25 | nonICU_vs_healthy |
| OID01259 | TCN2 | Olink CARDIOMETABOLIC | 1,144088326 | 1,30145E-25 | 1,193E-24 | nonICU_vs_healthy |
| OID01299 | CNDP1 | Olink CARDIOMETABOLIC | 1,231523475 | 2,69266E-25 | 2,36954E-24 | nonICU_vs_healthy |
| OID01281 | EFEMP1 | Olink CARDIOMETABOLIC | 1,365945232 | 1,11877E-23 | 9,46651E-23 | nonICU_vs_healthy |
| OID01286 | GAS6 | Olink CARDIOMETABOLIC | 0,762612195 | 8,47023E-23 | 6,90167E-22 | nonICU_vs_healthy |
| OID00391 | TNFRSF10A | Olink CARDIOVASCULAR II | 1,068616608 | 1,98411E-22 | 1,55894E-21 | nonICU_vs_healthy |
| OID00393 | IDUA | Olink CARDIOVASCULAR II | 2,045120193 | 7,33452E-22 | 5,56412E-21 | nonICU_vs_healthy |
| OID00433 | XCL1 | Olink CARDIOVASCULAR II | 1,688496139 | 4,2902E-21 | 3,14615E-20 | nonICU_vs_healthy |
| OID00453 | MARCO | Olink CARDIOVASCULAR II | 0,821220675 | 7,27354E-21 | 5,16187E-20 | nonICU_vs_healthy |
| OID05547 | IFN-gamma | Olink INFLAMMATION | 3,498738588 | 1,34121E-20 | 9,2208E-20 | nonICU_vs_healthy |
| OID01284 | PTPRS | Olink CARDIOMETABOLIC | -0,406525697 | 8,92788E-20 | 5,95192E-19 | nonICU_vs_healthy |
| OID00448 | AGRP | Olink CARDIOVASCULAR II | 1,270017594 | 2,83437E-19 | 1,83401E-18 | nonICU_vs_healthy |
| OID01224 | TIMP1 | Olink CARDIOMETABOLIC | 0,757261148 | 8,30523E-19 | 5,22043E-18 | nonICU_vs_healthy |
| OID00431 | PRELP | Olink CARDIOVASCULAR II | 0,539882845 | 1,24199E-18 | 7,58995E-18 | nonICU_vs_healthy |
| OID01258 | CR2 | Olink CARDIOMETABOLIC | -1,15649787 | 1,27996E-18 | 7,6106E-18 | nonICU_vs_healthy |
| OID01289 | PCOLCE | Olink CARDIOMETABOLIC | -0,783457814 | 1,629E-18 | 9,43106E-18 | nonICU_vs_healthy |
| OID00513 | CCL19 | Olink INFLAMMATION | 1,803387805 | 5,13745E-18 | 2,89805E-17 | nonICU_vs_healthy |
| OID00416 | SPON2 | Olink CARDIOVASCULAR II | 0,368874765 | 8,5953E-18 | 4,72742E-17 | nonICU_vs_healthy |
| OID00535 | CXCL10 | Olink INFLAMMATION | 2,021951356 | 9,1346E-18 | 4,90149E-17 | nonICU_vs_healthy |
| OID00476 | CDCP1 | Olink INFLAMMATION | 0,995471305 | 1,84663E-17 | 9,67283E-17 | nonICU_vs_healthy |
| OID00479 | OPG | Olink INFLAMMATION | 0,644278318 | 2,5232E-17 | 1,29094E-16 | nonICU_vs_healthy |
| OID00552 | CX3CL1 | Olink INFLAMMATION | 1,003530176 | 3,17898E-17 | 1,58949E-16 | nonICU_vs_healthy |
| OID01266 | DPP4 | Olink CARDIOMETABOLIC | -0,635107734 | 4,19692E-17 | 2,05183E-16 | nonICU_vs_healthy |
| OID00457 | ACE2 | Olink CARDIOVASCULAR II | 1,442006304 | 5,47128E-17 | 2,6167E-16 | nonICU_vs_healthy |
| OID01213 | DNER | Olink INFLAMMATION | -0,492098843 | 7,48639E-17 | 3,50427E-16 | nonICU_vs_healthy |
| OID01265 | TNC | Olink CARDIOMETABOLIC | 1,211417118 | 1,12473E-16 | 5,15501E-16 | nonICU_vs_healthy |
| OID01241 | KIT | Olink CARDIOMETABOLIC | -0,616581643 | 1,16145E-16 | 5,21466E-16 | nonICU_vs_healthy |
| OID01221 | APOM | Olink CARDIOMETABOLIC | -0,756486198 | 1,86705E-16 | 8,21503E-16 | nonICU_vs_healthy |
| OID00486 | CXCL11 | Olink INFLAMMATION | 1,878325914 | 2,40263E-16 | 1,03643E-15 | nonICU_vs_healthy |
| OID01252 | ST6GAL1 | Olink CARDIOMETABOLIC | 0,756093092 | 2,94652E-16 | 1,2466E-15 | nonICU_vs_healthy |
| OID00500 | SCF | Olink INFLAMMATION | -1,196786276 | 3,05034E-16 | 1,26618E-15 | nonICU_vs_healthy |
| OID00536 | 4E-BP1 | Olink INFLAMMATION | 2,166202796 | 7,65013E-16 | 3,11672E-15 | nonICU_vs_healthy |
| OID00397 | PRSS27 | Olink CARDIOVASCULAR II | -0,938491413 | 1,06798E-15 | 4,27191E-15 | nonICU_vs_healthy |
| OID00418 | FS | Olink CARDIOVASCULAR II | 0,9096181 | 1,98336E-15 | 7,79176E-15 | nonICU_vs_healthy |
| OID01257 | VCAM1 | Olink CARDIOMETABOLIC | 0,679342007 | 9,4566E-15 | 3,64992E-14 | nonICU_vs_healthy |
| OID00398 | TIE2 | Olink CARDIOVASCULAR II | -0,436141995 | 2,20826E-14 | 8,37616E-14 | nonICU_vs_healthy |
| OID00459 | CTSL1 | Olink CARDIOVASCULAR II | 0,907771092 | 3,10403E-14 | 1,15743E-13 | nonICU_vs_healthy |
| OID00472 | VEGFA | Olink INFLAMMATION | 0,956098522 | 4,98357E-14 | 1,82731E-13 | nonICU_vs_healthy |
| OID00408 | SCF | Olink CARDIOVASCULAR II | -1,070469548 | 7,39822E-14 | 2,66821E-13 | nonICU_vs_healthy |
| OID00426 | KIM1 | Olink CARDIOVASCULAR II | 1,32390475 | 1,14779E-13 | 4,07279E-13 | nonICU_vs_healthy |
| OID01253 | IL7R | Olink CARDIOMETABOLIC | -0,749276087 | 1,19346E-13 | 4,16764E-13 | nonICU_vs_healthy |
| OID00542 | CD40 | Olink INFLAMMATION | 0,77265934 | 2,09005E-13 | 7,18455E-13 | nonICU_vs_healthy |
| OID00439 | CCL17 | Olink CARDIOVASCULAR II | -1,672172351 | 2,41714E-13 | 8,1811E-13 | nonICU_vs_healthy |
| OID01296 | LILRB2 | Olink CARDIOMETABOLIC | 0,651334784 | 2,52664E-13 | 8,42214E-13 | nonICU_vs_healthy |
| OID00481 | uPA | Olink INFLAMMATION | 0,553087869 | 2,93721E-13 | 9,64456E-13 | nonICU_vs_healthy |
| OID01268 | THBS4 | Olink CARDIOMETABOLIC | -1,011200274 | 6,10022E-13 | 1,9736E-12 | nonICU_vs_healthy |
| OID01302 | CFHR5 | Olink CARDIOMETABOLIC | 0,729452477 | 7,77135E-13 | 2,47782E-12 | nonICU_vs_healthy |
| OID01254 | ENG | Olink CARDIOMETABOLIC | -0,284829026 | 9,51527E-13 | 2,99051E-12 | nonICU_vs_healthy |
| OID01297 | LILRB1 | Olink CARDIOMETABOLIC | 0,512674135 | 1,37306E-12 | 4,25455E-12 | nonICU_vs_healthy |
| OID00460 | hOSCAR | Olink CARDIOVASCULAR II | 0,348702569 | 1,48935E-11 | 4,5508E-11 | nonICU_vs_healthy |
| OID00555 | TWEAK | Olink INFLAMMATION | 0,914318092 | 2,99629E-11 | 9,02993E-11 | nonICU_vs_healthy |
| OID00484 | MCP-1 | Olink INFLAMMATION | 0,911976427 | 3,43634E-11 | 1,02162E-10 | nonICU_vs_healthy |
| OID00512 | FGF-21 | Olink INFLAMMATION | 1,922536559 | 5,66146E-10 | 1,66069E-09 | nonICU_vs_healthy |
| OID00412 | RAGE | Olink CARDIOVASCULAR II | 0,647271343 | 8,41793E-10 | 2,43677E-09 | nonICU_vs_healthy |
| OID00446 | LPL | Olink CARDIOVASCULAR II | 0,653655056 | 9,22027E-10 | 2,63436E-09 | nonICU_vs_healthy |
| OID00410 | FGF-21 | Olink CARDIOVASCULAR II | 2,033165371 | 1,28752E-09 | 3,5931E-09 | nonICU_vs_healthy |
| OID01240 | IGLC2 | Olink CARDIOMETABOLIC | 0,786140263 | 1,29025E-09 | 3,5931E-09 | nonICU_vs_healthy |
| OID00470 | HAOX1 | Olink CARDIOVASCULAR II | 2,144503489 | 2,61239E-09 | 7,18407E-09 | nonICU_vs_healthy |
| OID00556 | CCL20 | Olink INFLAMMATION | 1,020588057 | 4,98817E-09 | 1,35481E-08 | nonICU_vs_healthy |
| OID00541 | EN-RAGE | Olink INFLAMMATION | -1,217150157 | 8,53308E-09 | 2,28936E-08 | nonICU_vs_healthy |
| OID00385 | ADAM-TS13 | Olink CARDIOVASCULAR II | -0,205142406 | 9,51963E-09 | 2,52328E-08 | nonICU_vs_healthy |
| OID01220 | LILRB5 | Olink CARDIOMETABOLIC | -0,89501881 | 1,3329E-08 | 3,49092E-08 | nonICU_vs_healthy |
| OID00532 | CCL3 | Olink INFLAMMATION | 0,709608012 | 1,4272E-08 | 3,69393E-08 | nonICU_vs_healthy |
| OID00549 | MCP-2 | Olink INFLAMMATION | 1,103235257 | 3,09429E-08 | 7,91564E-08 | nonICU_vs_healthy |
| OID00560 | ADA | Olink INFLAMMATION | 0,699543058 | 6,67592E-08 | 1,68816E-07 | nonICU_vs_healthy |
| OID00396 | TRAIL-R2 | Olink CARDIOVASCULAR II | 0,674724987 | 9,24772E-08 | 2,31193E-07 | nonICU_vs_healthy |
| OID00394 | TNFRSF11A | Olink CARDIOVASCULAR II | 0,604791461 | 9,98261E-08 | 2,46761E-07 | nonICU_vs_healthy |
| OID00465 | HSP 27 | Olink CARDIOVASCULAR II | -0,452313502 | 1,13396E-07 | 2,7719E-07 | nonICU_vs_healthy |
| OID00407 | GIF | Olink CARDIOVASCULAR II | -1,070464157 | 1,25113E-07 | 3,02472E-07 | nonICU_vs_healthy |
| OID01255 | IGFBP3 | Olink CARDIOMETABOLIC | -0,530316282 | 1,37267E-07 | 3,28247E-07 | nonICU_vs_healthy |
| OID00389 | IL-1ra | Olink CARDIOVASCULAR II | 0,91784853 | 2,08395E-07 | 4,92978E-07 | nonICU_vs_healthy |
| OID00417 | GH | Olink CARDIOVASCULAR II | 1,54856406 | 2,64064E-07 | 6,18022E-07 | nonICU_vs_healthy |
| OID01294 | AOC3 | Olink CARDIOMETABOLIC | -0,295200698 | 3,30675E-07 | 7,65773E-07 | nonICU_vs_healthy |
| OID00427 | THBS2 | Olink CARDIOVASCULAR II | 0,226257468 | 5,48824E-07 | 1,25772E-06 | nonICU_vs_healthy |
| OID01216 | CHL1 | Olink CARDIOMETABOLIC | -0,35702427 | 5,61657E-07 | 1,27386E-06 | nonICU_vs_healthy |
| OID01307 | LYVE1 | Olink CARDIOMETABOLIC | -0,405587058 | 6,64739E-07 | 1,49227E-06 | nonICU_vs_healthy |
| OID00523 | IL-12B | Olink INFLAMMATION | 0,788916409 | 1,52448E-06 | 3,38774E-06 | nonICU_vs_healthy |
| OID00454 | GT | Olink CARDIOVASCULAR II | 0,812754593 | 1,66881E-06 | 3,67137E-06 | nonICU_vs_healthy |
| OID00504 | MCP-4 | Olink INFLAMMATION | -0,778655664 | 1,74336E-06 | 3,79741E-06 | nonICU_vs_healthy |
| OID00438 | PSGL-1 | Olink CARDIOVASCULAR II | -0,245463148 | 4,71022E-06 | 1,01593E-05 | nonICU_vs_healthy |
| OID00522 | HGF | Olink INFLAMMATION | 0,654790316 | 5,93083E-06 | 1,26678E-05 | nonICU_vs_healthy |
| OID01291 | TGFBI | Olink CARDIOMETABOLIC | 0,357376446 | 6,34246E-06 | 1,34167E-05 | nonICU_vs_healthy |
| OID00384 | PGF | Olink CARDIOVASCULAR II | 0,453651796 | 7,00737E-06 | 1,46821E-05 | nonICU_vs_healthy |
| OID01301 | C1QTNF1 | Olink CARDIOMETABOLIC | 0,404570939 | 1,09564E-05 | 2,27397E-05 | nonICU_vs_healthy |
| OID00401 | PDGF subunit B | Olink CARDIOVASCULAR II | -0,937520469 | 1,12559E-05 | 2,3143E-05 | nonICU_vs_healthy |
| OID01230 | ICAM1 | Olink CARDIOMETABOLIC | 0,322008812 | 1,32933E-05 | 2,70789E-05 | nonICU_vs_healthy |
| OID00558 | STAMBP | Olink INFLAMMATION | 0,769934414 | 1,65871E-05 | 3,34786E-05 | nonICU_vs_healthy |
| OID01226 | ANG | Olink CARDIOMETABOLIC | 0,324647903 | 1,77591E-05 | 3,55183E-05 | nonICU_vs_healthy |
| OID00499 | CD6 | Olink INFLAMMATION | -0,624954173 | 2,07884E-05 | 4,12023E-05 | nonICU_vs_healthy |
| OID00490 | CXCL9 | Olink INFLAMMATION | 0,864218069 | 2,1008E-05 | 4,12656E-05 | nonICU_vs_healthy |
| OID00445 | Dkk-1 | Olink CARDIOVASCULAR II | -0,583469833 | 2,17511E-05 | 4,23473E-05 | nonICU_vs_healthy |
| OID00510 | MMP-1 | Olink INFLAMMATION | 1,276265571 | 2,8183E-05 | 5,43883E-05 | nonICU_vs_healthy |
| OID01233 | C2 | Olink CARDIOMETABOLIC | -0,390485789 | 3,22141E-05 | 6,16269E-05 | nonICU_vs_healthy |
| OID00411 | PIgR | Olink CARDIOVASCULAR II | 0,154197662 | 3,80397E-05 | 7,21442E-05 | nonICU_vs_healthy |
| OID00511 | LIF-R | Olink INFLAMMATION | 0,284882576 | 5,26273E-05 | 9,89574E-05 | nonICU_vs_healthy |
| OID00487 | AXIN1 | Olink INFLAMMATION | 0,920268482 | 7,32322E-05 | 0,000136535 | nonICU_vs_healthy |
| OID00443 | ITGB1BP2 | Olink CARDIOVASCULAR II | 0,886142089 | 8,31954E-05 | 0,000153807 | nonICU_vs_healthy |
| OID01236 | PRSS2 | Olink CARDIOMETABOLIC | 0,591555896 | 9,26018E-05 | 0,00016977 | nonICU_vs_healthy |
| OID00435 | SORT1 | Olink CARDIOVASCULAR II | -0,27579904 | 9,55775E-05 | 0,000173777 | nonICU_vs_healthy |
| OID01218 | PLXNB2 | Olink CARDIOMETABOLIC | 0,175515788 | 9,78475E-05 | 0,000176446 | nonICU_vs_healthy |
| OID00405 | LOX-1 | Olink CARDIOVASCULAR II | -0,686824352 | 0,00017602 | 0,000314832 | nonICU_vs_healthy |
| OID00449 | HB-EGF | Olink CARDIOVASCULAR II | -0,713804203 | 0,000191762 | 0,000340223 | nonICU_vs_healthy |
| OID01234 | GP1BA | Olink CARDIOMETABOLIC | -0,337967852 | 0,000248283 | 0,000436979 | nonICU_vs_healthy |
| OID01243 | MBL2 | Olink CARDIOMETABOLIC | 0,77317421 | 0,000463563 | 0,000809395 | nonICU_vs_healthy |
| OID00440 | CCL3 | Olink CARDIOVASCULAR II | 0,490822566 | 0,000468671 | 0,00081187 | nonICU_vs_healthy |
| OID00452 | THPO | Olink CARDIOVASCULAR II | 0,431604961 | 0,000559382 | 0,000961437 | nonICU_vs_healthy |
| OID00539 | CCL28 | Olink INFLAMMATION | -0,321738175 | 0,000611664 | 0,001043147 | nonICU_vs_healthy |
| OID01298 | TIMD4 | Olink CARDIOMETABOLIC | 0,377475843 | 0,000653597 | 0,001106087 | nonICU_vs_healthy |
| OID00503 | TGF-alpha | Olink INFLAMMATION | 0,400521194 | 0,000708706 | 0,001190193 | nonICU_vs_healthy |
| OID01293 | QPCT | Olink CARDIOMETABOLIC | 0,1971609 | 0,000826219 | 0,001377032 | nonICU_vs_healthy |
| OID01251 | CD46 | Olink CARDIOMETABOLIC | -0,220885548 | 0,000860455 | 0,001423308 | nonICU_vs_healthy |
| OID01247 | NCAM1 | Olink CARDIOMETABOLIC | -0,164549445 | 0,000922072 | 0,001513849 | nonICU_vs_healthy |
| OID01244 | FCGR2A | Olink CARDIOMETABOLIC | -0,418409576 | 0,001052898 | 0,001707037 | nonICU_vs_healthy |
| OID01245 | CDH1 | Olink CARDIOMETABOLIC | -0,231280906 | 0,001055259 | 0,001707037 | nonICU_vs_healthy |
| OID00423 | REN | Olink CARDIOVASCULAR II | 0,49292892 | 0,001287595 | 0,002067671 | nonICU_vs_healthy |
| OID01303 | MEGF9 | Olink CARDIOMETABOLIC | 0,177897303 | 0,001533159 | 0,002444166 | nonICU_vs_healthy |
| OID01223 | CA1 | Olink CARDIOMETABOLIC | 0,667280783 | 0,001594799 | 0,002524142 | nonICU_vs_healthy |
| OID00471 | IL8 | Olink INFLAMMATION | 0,667712554 | 0,001698112 | 0,002668462 | nonICU_vs_healthy |
| OID00444 | DCN | Olink CARDIOVASCULAR II | 0,257490075 | 0,00174236 | 0,002718576 | nonICU_vs_healthy |
| OID00430 | AMBP | Olink CARDIOVASCULAR II | 0,153539453 | 0,002215048 | 0,003431765 | nonICU_vs_healthy |
| OID00480 | LAP TGF-beta-1 | Olink INFLAMMATION | -0,370440123 | 0,002246756 | 0,003456547 | nonICU_vs_healthy |
| OID00545 | FGF-19 | Olink INFLAMMATION | -0,601293451 | 0,002306351 | 0,003523591 | nonICU_vs_healthy |
| OID00520 | CXCL5 | Olink INFLAMMATION | -0,867800171 | 0,003230196 | 0,004900987 | nonICU_vs_healthy |
| OID01270 | TIE1 | Olink CARDIOMETABOLIC | -0,128816633 | 0,004543535 | 0,006846423 | nonICU_vs_healthy |
| OID00506 | TNFSF14 | Olink INFLAMMATION | 0,460978483 | 0,00540269 | 0,008085659 | nonICU_vs_healthy |
| OID00436 | CEACAM8 | Olink CARDIOVASCULAR II | -0,493001592 | 0,005953642 | 0,008829815 | nonICU_vs_healthy |
| OID01246 | CCL5 | Olink CARDIOMETABOLIC | -0,660491548 | 0,005980193 | 0,008829815 | nonICU_vs_healthy |
| OID00380 | ANGPT1 | Olink CARDIOVASCULAR II | -0,601723112 | 0,006501546 | 0,009535601 | nonICU_vs_healthy |
| OID01256 | PAM | Olink CARDIOMETABOLIC | 0,136535958 | 0,008323769 | 0,012127346 | nonICU_vs_healthy |
| OID01287 | SPARCL1 | Olink CARDIOMETABOLIC | 0,155891418 | 0,008426152 | 0,012195746 | nonICU_vs_healthy |
| OID01290 | FCN2 | Olink CARDIOMETABOLIC | 0,281564241 | 0,010543194 | 0,015160149 | nonICU_vs_healthy |
| OID00491 | CST5 | Olink INFLAMMATION | -0,274885319 | 0,010761032 | 0,015372903 | nonICU_vs_healthy |
| OID01269 | SAA4 | Olink CARDIOMETABOLIC | -0,351423741 | 0,012963728 | 0,01840013 | nonICU_vs_healthy |
| OID01238 | MET | Olink CARDIOMETABOLIC | 0,116481454 | 0,013943515 | 0,019663932 | nonICU_vs_healthy |
| OID01225 | CST3 | Olink CARDIOMETABOLIC | 0,260842196 | 0,014732182 | 0,020643822 | nonICU_vs_healthy |
| OID00477 | CD244 | Olink INFLAMMATION | -0,178155239 | 0,017046598 | 0,02373577 | nonICU_vs_healthy |
| OID00478 | IL7 | Olink INFLAMMATION | 0,347070127 | 0,017641241 | 0,024409264 | nonICU_vs_healthy |
| OID01276 | CCL18 | Olink CARDIOMETABOLIC | 0,297937219 | 0,021401162 | 0,029426597 | nonICU_vs_healthy |
| OID00551 | CCL25 | Olink INFLAMMATION | 0,282785174 | 0,023749498 | 0,03245273 | nonICU_vs_healthy |
| OID01227 | F11 | Olink CARDIOMETABOLIC | 0,156375384 | 0,023996618 | 0,032587999 | nonICU_vs_healthy |
| OID00420 | CD84 | Olink CARDIOVASCULAR II | -0,184399653 | 0,039060195 | 0,052719282 | nonICU_vs_healthy |
| OID01239 | F7 | Olink CARDIOMETABOLIC | 0,12545194 | 0,05032601 | 0,067510502 | nonICU_vs_healthy |
| OID00388 | SRC | Olink CARDIOVASCULAR II | -0,384296173 | 0,052266771 | 0,069689028 | nonICU_vs_healthy |
| OID01271 | COL18A1 | Olink CARDIOMETABOLIC | 0,136156172 | 0,056124971 | 0,074382492 | nonICU_vs_healthy |
| OID00409 | IL18 | Olink CARDIOVASCULAR II | 0,353903428 | 0,05890952 | 0,077605355 | nonICU_vs_healthy |
| OID00468 | VEGFD | Olink CARDIOVASCULAR II | 0,160042724 | 0,064085402 | 0,08392136 | nonICU_vs_healthy |
| OID00469 | PARP-1 | Olink CARDIOVASCULAR II | -0,431993107 | 0,071321409 | 0,092844438 | nonICU_vs_healthy |
| OID01217 | NRP1 | Olink CARDIOMETABOLIC | 0,074484594 | 0,07912477 | 0,102396761 | nonICU_vs_healthy |
| OID00533 | Flt3L | Olink INFLAMMATION | -0,219203318 | 0,101027643 | 0,129977085 | nonICU_vs_healthy |
| OID01228 | PROC | Olink CARDIOMETABOLIC | -0,138049509 | 0,104267301 | 0,133365153 | nonICU_vs_healthy |
| OID00395 | PAR-1 | Olink CARDIOVASCULAR II | -0,145330604 | 0,112662598 | 0,143270356 | nonICU_vs_healthy |
| OID01273 | NOTCH1 | Olink CARDIOMETABOLIC | -0,063035578 | 0,116101629 | 0,146795163 | nonICU_vs_healthy |
| OID00382 | CD40-L | Olink CARDIOVASCULAR II | 0,41988294 | 0,123539659 | 0,155307 | nonICU_vs_healthy |
| OID00456 | MMP12 | Olink CARDIOVASCULAR II | 0,238006299 | 0,125462867 | 0,156828584 | nonICU_vs_healthy |
| OID01295 | VASN | Olink CARDIOMETABOLIC | -0,060726077 | 0,140688379 | 0,174866912 | nonICU_vs_healthy |
| OID00515 | IL-10RB | Olink INFLAMMATION | -0,086853795 | 0,14866954 | 0,18374887 | nonICU_vs_healthy |
| OID00461 | TNFRSF13B | Olink CARDIOVASCULAR II | -0,234389148 | 0,152204758 | 0,1870673 | nonICU_vs_healthy |
| OID01262 | GNLY | Olink CARDIOMETABOLIC | 0,102786587 | 0,153686042 | 0,187838496 | nonICU_vs_healthy |
| OID00505 | CCL11 | Olink INFLAMMATION | -0,148686017 | 0,212594482 | 0,258402133 | nonICU_vs_healthy |
| OID00434 | IL16 | Olink CARDIOVASCULAR II | -0,180719592 | 0,214897515 | 0,259464581 | nonICU_vs_healthy |
| OID01219 | FCGR3B | Olink CARDIOMETABOLIC | -0,124992988 | 0,215827356 | 0,259464581 | nonICU_vs_healthy |
| OID01306 | ANGPTL3 | Olink CARDIOMETABOLIC | -0,096702872 | 0,225496156 | 0,269614969 | nonICU_vs_healthy |
| OID00534 | CXCL6 | Olink INFLAMMATION | 0,214256115 | 0,230393082 | 0,273980962 | nonICU_vs_healthy |
| OID00494 | OSM | Olink INFLAMMATION | 0,255227549 | 0,232196972 | 0,27464158 | nonICU_vs_healthy |
| OID01264 | IGFBP6 | Olink CARDIOMETABOLIC | -0,100033711 | 0,262240195 | 0,308517877 | nonICU_vs_healthy |
| OID01232 | SERPINA7 | Olink CARDIOMETABOLIC | 0,074552441 | 0,287521032 | 0,336460782 | nonICU_vs_healthy |
| OID01231 | REG1A | Olink CARDIOMETABOLIC | 0,144014054 | 0,304265688 | 0,3541717 | nonICU_vs_healthy |
| OID00501 | IL18 | Olink INFLAMMATION | 0,182383144 | 0,324222679 | 0,375415734 | nonICU_vs_healthy |
| OID00488 | TRAIL | Olink INFLAMMATION | 0,076573142 | 0,349883046 | 0,401545558 | nonICU_vs_healthy |
| OID00498 | CCL4 | Olink INFLAMMATION | 0,130358299 | 0,350439759 | 0,401545558 | nonICU_vs_healthy |
| OID00561 | TNFB | Olink INFLAMMATION | -0,095152855 | 0,362364559 | 0,413058047 | nonICU_vs_healthy |
| OID01249 | SELL | Olink CARDIOMETABOLIC | 0,072542785 | 0,376350738 | 0,426789497 | nonICU_vs_healthy |
| OID01267 | ICAM3 | Olink CARDIOMETABOLIC | -0,055840823 | 0,392052633 | 0,442315791 | nonICU_vs_healthy |
| OID00392 | STK4 | Olink CARDIOVASCULAR II | 0,198884918 | 0,397391035 | 0,446051162 | nonICU_vs_healthy |
| OID00531 | CD5 | Olink INFLAMMATION | -0,086137441 | 0,406546394 | 0,454011201 | nonICU_vs_healthy |
| OID00467 | NEMO | Olink CARDIOVASCULAR II | 0,177270256 | 0,413565431 | 0,459517145 | nonICU_vs_healthy |
| OID00458 | PD-L2 | Olink CARDIOVASCULAR II | -0,059298493 | 0,433891009 | 0,479678502 | nonICU_vs_healthy |
| OID01300 | OSMR | Olink CARDIOMETABOLIC | -0,033290414 | 0,450416288 | 0,495457916 | nonICU_vs_healthy |
| OID00400 | IL1RL2 | Olink CARDIOVASCULAR II | 0,061297032 | 0,459432451 | 0,502861389 | nonICU_vs_healthy |
| OID00442 | IgG Fc receptor II-b | Olink CARDIOVASCULAR II | 0,084135871 | 0,54541443 | 0,594015715 | nonICU_vs_healthy |
| OID00413 | SOD2 | Olink CARDIOVASCULAR II | 0,012226058 | 0,58813124 | 0,637383609 | nonICU_vs_healthy |
| OID01248 | CD59 | Olink CARDIOMETABOLIC | 0,03100983 | 0,610568231 | 0,658455935 | nonICU_vs_healthy |
| OID00428 | TM | Olink CARDIOVASCULAR II | -0,039849769 | 0,617388494 | 0,662563261 | nonICU_vs_healthy |
| OID00447 | PRSS8 | Olink CARDIOVASCULAR II | 0,037123598 | 0,6292783 | 0,672044787 | nonICU_vs_healthy |
| OID00422 | SERPINA12 | Olink CARDIOVASCULAR II | -0,094038024 | 0,63320081 | 0,672967045 | nonICU_vs_healthy |
| OID00414 | CTRC | Olink CARDIOVASCULAR II | -0,077974497 | 0,649413957 | 0,686880147 | nonICU_vs_healthy |
| OID01235 | CA3 | Olink CARDIOMETABOLIC | 0,055060066 | 0,667114856 | 0,702226164 | nonICU_vs_healthy |
| OID00399 | TF | Olink CARDIOVASCULAR II | -0,03099402 | 0,671599572 | 0,703580504 | nonICU_vs_healthy |
| OID00429 | VSIG2 | Olink CARDIOVASCULAR II | 0,049787361 | 0,677141703 | 0,706024525 | nonICU_vs_healthy |
| OID01305 | FETUB | Olink CARDIOMETABOLIC | -0,041969285 | 0,710762779 | 0,737584015 | nonICU_vs_healthy |
| OID00463 | LEP | Olink CARDIOVASCULAR II | 0,070118558 | 0,740292986 | 0,764621864 | nonICU_vs_healthy |
| OID00496 | CXCL1 | Olink INFLAMMATION | 0,049863905 | 0,774245608 | 0,795953429 | nonICU_vs_healthy |
| OID00553 | TNFRSF9 | Olink INFLAMMATION | 0,032868772 | 0,794381866 | 0,812855863 | nonICU_vs_healthy |
| OID00451 | FABP2 | Olink CARDIOVASCULAR II | 0,037900527 | 0,860767254 | 0,876707389 | nonICU_vs_healthy |
| OID00527 | MMP-10 | Olink INFLAMMATION | -0,012678343 | 0,930570329 | 0,943435357 | nonICU_vs_healthy |
| OID01292 | CCL14 | Olink CARDIOMETABOLIC | 0,004413182 | 0,960670725 | 0,969484218 | nonICU_vs_healthy |
| OID00462 | TGM2 | Olink CARDIOVASCULAR II | 0,005139467 | 0,969284498 | 0,973710455 | nonICU_vs_healthy |
| OID00404 | CXCL1 | Olink CARDIOVASCULAR II | -0,006023329 | 0,974682447 | 0,974682447 | nonICU_vs_healthy |
| OID00555 | TWEAK | Olink INFLAMMATION | 1,161921958 | 2,19165E-22 | 6,6626E-20 | convalescent_vs_healthy |
| OID01281 | EFEMP1 | Olink CARDIOMETABOLIC | 0,71664325 | 4,24517E-17 | 6,45265E-15 | convalescent_vs_healthy |
| OID00441 | MMP7 | Olink CARDIOVASCULAR II | 1,615400907 | 3,60534E-16 | 3,65341E-14 | convalescent_vs_healthy |
| OID00381 | ADM | Olink CARDIOVASCULAR II | 1,618663299 | 5,33359E-15 | 4,05353E-13 | convalescent_vs_healthy |
| OID00404 | CXCL1 | Olink CARDIOVASCULAR II | 0,978452594 | 1,9246E-14 | 1,00511E-12 | convalescent_vs_healthy |
| OID00466 | CD4 | Olink CARDIOVASCULAR II | 0,433591264 | 1,98377E-14 | 1,00511E-12 | convalescent_vs_healthy |
| OID00308 | CD38 | Olink NEUROLOGY | 0,739286737 | 3,03633E-14 | 1,31863E-12 | convalescent_vs_healthy |
| OID00446 | LPL | Olink CARDIOVASCULAR II | 0,651371463 | 4,6557E-14 | 1,76917E-12 | convalescent_vs_healthy |
| OID00496 | CXCL1 | Olink INFLAMMATION | 0,958537332 | 6,08602E-14 | 2,05572E-12 | convalescent_vs_healthy |
| OID00379 | BMP-6 | Olink CARDIOVASCULAR II | 1,462632782 | 1,17971E-13 | 3,58632E-12 | convalescent_vs_healthy |
| OID00378 | KYNU | Olink NEUROLOGY | 1,19661663 | 1,14875E-12 | 3,17474E-11 | convalescent_vs_healthy |
| OID00462 | TGM2 | Olink CARDIOVASCULAR II | -1,036430186 | 1,49343E-11 | 3,78335E-10 | convalescent_vs_healthy |
| OID00443 | ITGB1BP2 | Olink CARDIOVASCULAR II | 1,766091936 | 2,89446E-11 | 6,76857E-10 | convalescent_vs_healthy |
| OID00459 | CTSL1 | Olink CARDIOVASCULAR II | -0,39398347 | 7,53421E-11 | 1,636E-09 | convalescent_vs_healthy |
| OID01301 | C1QTNF1 | Olink CARDIOMETABOLIC | -0,610573032 | 9,70973E-11 | 1,96784E-09 | convalescent_vs_healthy |
| OID00321 | RSPO1 | Olink NEUROLOGY | 0,490296396 | 1,40553E-10 | 2,6705E-09 | convalescent_vs_healthy |
| OID00557 | ST1A1 | Olink INFLAMMATION | 1,363401904 | 2,51484E-10 | 4,49712E-09 | convalescent_vs_healthy |
| OID05548 | TNF | Olink INFLAMMATION | 0,545809433 | 3,53626E-10 | 5,97235E-09 | convalescent_vs_healthy |
| OID00393 | IDUA | Olink CARDIOVASCULAR II | 0,948881146 | 1,89155E-09 | 3,02649E-08 | convalescent_vs_healthy |
| OID01272 | PRCP | Olink CARDIOMETABOLIC | 0,379354278 | 2,87449E-09 | 4,36923E-08 | convalescent_vs_healthy |
| OID01304 | CRTAC1 | Olink CARDIOMETABOLIC | 0,405840219 | 7,41714E-09 | 1,07372E-07 | convalescent_vs_healthy |
| OID00398 | TIE2 | Olink CARDIOVASCULAR II | -0,211552543 | 8,38117E-09 | 1,15813E-07 | convalescent_vs_healthy |
| OID01229 | SERPINA5 | Olink CARDIOMETABOLIC | -0,462331327 | 1,61834E-08 | 2,13903E-07 | convalescent_vs_healthy |
| OID00448 | AGRP | Olink CARDIOVASCULAR II | 0,443159929 | 1,73727E-08 | 2,20055E-07 | convalescent_vs_healthy |
| OID00510 | MMP-1 | Olink INFLAMMATION | 1,24185824 | 2,44018E-08 | 2,96726E-07 | convalescent_vs_healthy |
| OID00488 | TRAIL | Olink INFLAMMATION | 0,294680741 | 4,50841E-08 | 5,27137E-07 | convalescent_vs_healthy |
| OID00389 | IL-1ra | Olink CARDIOVASCULAR II | -0,857029877 | 1,10893E-07 | 1,24858E-06 | convalescent_vs_healthy |
| OID01287 | SPARCL1 | Olink CARDIOMETABOLIC | 0,239363053 | 2,28259E-07 | 2,47824E-06 | convalescent_vs_healthy |
| OID00382 | CD40-L | Olink CARDIOVASCULAR II | 1,070794561 | 2,48807E-07 | 2,60818E-06 | convalescent_vs_healthy |
| OID00409 | IL18 | Olink CARDIOVASCULAR II | -0,759121429 | 2,65332E-07 | 2,6887E-06 | convalescent_vs_healthy |
| OID00339 | GZMA | Olink NEUROLOGY | 0,537096633 | 3,63368E-07 | 3,56335E-06 | convalescent_vs_healthy |
| OID00520 | CXCL5 | Olink INFLAMMATION | 0,768036606 | 5,13578E-07 | 4,87899E-06 | convalescent_vs_healthy |
| OID00501 | IL18 | Olink INFLAMMATION | -0,747088727 | 6,03618E-07 | 5,5606E-06 | convalescent_vs_healthy |
| OID01233 | C2 | Olink CARDIOMETABOLIC | -0,406303046 | 6,82216E-07 | 6,09982E-06 | convalescent_vs_healthy |
| OID00530 | CCL23 | Olink INFLAMMATION | 0,349909991 | 7,7112E-07 | 6,69773E-06 | convalescent_vs_healthy |
| OID00465 | HSP 27 | Olink CARDIOVASCULAR II | -0,35302377 | 1,31226E-06 | 1,10813E-05 | convalescent_vs_healthy |
| OID01269 | SAA4 | Olink CARDIOMETABOLIC | -0,499404816 | 1,76339E-06 | 1,44884E-05 | convalescent_vs_healthy |
| OID00336 | SCARA5 | Olink NEUROLOGY | 0,357680126 | 2,01086E-06 | 1,60869E-05 | convalescent_vs_healthy |
| OID00558 | STAMBP | Olink INFLAMMATION | 1,084860978 | 2,11649E-06 | 1,64978E-05 | convalescent_vs_healthy |
| OID00320 | MATN3 | Olink NEUROLOGY | 0,258297179 | 2,4802E-06 | 1,88495E-05 | convalescent_vs_healthy |
| OID00419 | GLO1 | Olink CARDIOVASCULAR II | 0,777454803 | 2,96218E-06 | 2,19635E-05 | convalescent_vs_healthy |
| OID00431 | PRELP | Olink CARDIOVASCULAR II | 0,138603797 | 4,35786E-06 | 3,15426E-05 | convalescent_vs_healthy |
| OID00494 | OSM | Olink INFLAMMATION | -0,818850574 | 6,47929E-06 | 4,58071E-05 | convalescent_vs_healthy |
| OID00300 | SCARB2 | Olink NEUROLOGY | -0,211187161 | 7,76895E-06 | 5,36764E-05 | convalescent_vs_healthy |
| OID00487 | AXIN1 | Olink INFLAMMATION | 1,022618515 | 8,51389E-06 | 5,7516E-05 | convalescent_vs_healthy |
| OID00534 | CXCL6 | Olink INFLAMMATION | 0,614197749 | 9,29876E-06 | 6,04029E-05 | convalescent_vs_healthy |
| OID00456 | MMP12 | Olink CARDIOVASCULAR II | 0,460019822 | 9,3386E-06 | 6,04029E-05 | convalescent_vs_healthy |
| OID00301 | NCAN | Olink NEUROLOGY | 0,204785238 | 1,6992E-05 | 0,000107616 | convalescent_vs_healthy |
| OID00541 | EN-RAGE | Olink INFLAMMATION | -0,566621627 | 3,72953E-05 | 0,000231383 | convalescent_vs_healthy |
| OID00513 | CCL19 | Olink INFLAMMATION | 0,597599499 | 4,28664E-05 | 0,000260628 | convalescent_vs_healthy |
| OID05124 | CD8A | Olink INFLAMMATION | 0,530575179 | 5,12677E-05 | 0,000305596 | convalescent_vs_healthy |
| OID00360 | CTSS | Olink NEUROLOGY | -0,164060341 | 5,77089E-05 | 0,000337375 | convalescent_vs_healthy |
| OID01223 | CA1 | Olink CARDIOMETABOLIC | -0,643759473 | 0,000107883 | 0,000618803 | convalescent_vs_healthy |
| OID00480 | LAP TGF-beta-1 | Olink INFLAMMATION | 0,429287574 | 0,00012213 | 0,000687549 | convalescent_vs_healthy |
| OID00453 | MARCO | Olink CARDIOVASCULAR II | 0,258947987 | 0,000183491 | 0,001014207 | convalescent_vs_healthy |
| OID01221 | APOM | Olink CARDIOMETABOLIC | 0,167033225 | 0,000188867 | 0,001025279 | convalescent_vs_healthy |
| OID00468 | VEGFD | Olink CARDIOVASCULAR II | 0,211303704 | 0,00020203 | 0,001077494 | convalescent_vs_healthy |
| OID00319 | ADAM 23 | Olink NEUROLOGY | 0,23670274 | 0,00032025 | 0,00167855 | convalescent_vs_healthy |
| OID00357 | CDH6 | Olink NEUROLOGY | -0,133295404 | 0,000346651 | 0,001786133 | convalescent_vs_healthy |
| OID00385 | ADAM-TS13 | Olink CARDIOVASCULAR II | -0,138991474 | 0,000364394 | 0,001836875 | convalescent_vs_healthy |
| OID00417 | GH | Olink CARDIOVASCULAR II | 1,095195136 | 0,000368583 | 0,001836875 | convalescent_vs_healthy |
| OID00463 | LEP | Olink CARDIOVASCULAR II | -0,587162808 | 0,000484583 | 0,002367817 | convalescent_vs_healthy |
| OID00467 | NEMO | Olink CARDIOVASCULAR II | 0,740358915 | 0,000490699 | 0,002367817 | convalescent_vs_healthy |
| OID00425 | MERTK | Olink CARDIOVASCULAR II | 0,193519788 | 0,000535027 | 0,002541379 | convalescent_vs_healthy |
| OID00372 | NTRK3 | Olink NEUROLOGY | 0,126501399 | 0,000548099 | 0,002563418 | convalescent_vs_healthy |
| OID00430 | AMBP | Olink CARDIOVASCULAR II | -0,103924446 | 0,000611141 | 0,002814952 | convalescent_vs_healthy |
| OID00503 | TGF-alpha | Olink INFLAMMATION | -0,346402631 | 0,000641257 | 0,002909583 | convalescent_vs_healthy |
| OID00293 | VWC2 | Olink NEUROLOGY | 0,276131177 | 0,000770091 | 0,00344276 | convalescent_vs_healthy |
| OID00396 | TRAIL-R2 | Olink CARDIOVASCULAR II | -0,141368255 | 0,000782923 | 0,003449398 | convalescent_vs_healthy |
| OID00392 | STK4 | Olink CARDIOVASCULAR II | 0,646915482 | 0,000990273 | 0,004300616 | convalescent_vs_healthy |
| OID00539 | CCL28 | Olink INFLAMMATION | -0,227543251 | 0,001033083 | 0,00442334 | convalescent_vs_healthy |
| OID01256 | PAM | Olink CARDIOMETABOLIC | 0,136690447 | 0,001057239 | 0,004463898 | convalescent_vs_healthy |
| OID00452 | THPO | Olink CARDIOVASCULAR II | 0,251864579 | 0,001300283 | 0,005414878 | convalescent_vs_healthy |
| OID00343 | GDNFR-alpha-3 | Olink NEUROLOGY | 0,146240054 | 0,001425094 | 0,005847571 | convalescent_vs_healthy |
| OID00325 | BCAN | Olink NEUROLOGY | 0,187110651 | 0,001442657 | 0,005847571 | convalescent_vs_healthy |
| OID00350 | GCP5 | Olink NEUROLOGY | 0,355904582 | 0,00171664 | 0,006866561 | convalescent_vs_healthy |
| OID00394 | TNFRSF11A | Olink CARDIOVASCULAR II | -0,167969803 | 0,001765789 | 0,006971425 | convalescent_vs_healthy |
| OID01299 | CNDP1 | Olink CARDIOMETABOLIC | -0,292338435 | 0,00188023 | 0,007328075 | convalescent_vs_healthy |
| OID00311 | Alpha-2-MRAP | Olink NEUROLOGY | 0,367203264 | 0,002040738 | 0,007852968 | convalescent_vs_healthy |
| OID00434 | IL16 | Olink CARDIOVASCULAR II | -0,432894755 | 0,002301365 | 0,008745185 | convalescent_vs_healthy |
| OID00535 | CXCL10 | Olink INFLAMMATION | -0,344874495 | 0,002880437 | 0,01081053 | convalescent_vs_healthy |
| OID01235 | CA3 | Olink CARDIOMETABOLIC | -0,181538094 | 0,003345298 | 0,01240208 | convalescent_vs_healthy |
| OID00481 | uPA | Olink INFLAMMATION | 0,137451974 | 0,003845393 | 0,013954814 | convalescent_vs_healthy |
| OID00297 | SMOC2 | Olink NEUROLOGY | 0,201776528 | 0,003855935 | 0,013954814 | convalescent_vs_healthy |
| OID00406 | Gal-9 | Olink CARDIOVASCULAR II | -0,150795564 | 0,004864769 | 0,017398704 | convalescent_vs_healthy |
| OID00490 | CXCL9 | Olink INFLAMMATION | -0,307021723 | 0,004968376 | 0,017562631 | convalescent_vs_healthy |
| OID01265 | TNC | Olink CARDIOMETABOLIC | -0,217773824 | 0,005592353 | 0,019541096 | convalescent_vs_healthy |
| OID00418 | FS | Olink CARDIOVASCULAR II | 0,247071738 | 0,006186061 | 0,021370031 | convalescent_vs_healthy |
| OID00304 | CRTAM | Olink NEUROLOGY | 0,209301324 | 0,006484626 | 0,022149735 | convalescent_vs_healthy |
| OID00376 | CD200R1 | Olink NEUROLOGY | 0,146665082 | 0,00683685 | 0,023093359 | convalescent_vs_healthy |
| OID00356 | CTSC | Olink NEUROLOGY | -0,246964671 | 0,007301546 | 0,024391978 | convalescent_vs_healthy |
| OID00440 | CCL3 | Olink CARDIOVASCULAR II | -0,299445061 | 0,008724346 | 0,028828273 | convalescent_vs_healthy |
| OID01276 | CCL18 | Olink CARDIOMETABOLIC | -0,253638445 | 0,009056664 | 0,02960458 | convalescent_vs_healthy |
| OID00532 | CCL3 | Olink INFLAMMATION | -0,24595258 | 0,009265221 | 0,029964119 | convalescent_vs_healthy |
| OID00324 | gal-8 | Olink NEUROLOGY | 0,414821512 | 0,010819324 | 0,034621837 | convalescent_vs_healthy |
| OID01300 | OSMR | Olink CARDIOMETABOLIC | -0,07587622 | 0,011090583 | 0,035120179 | convalescent_vs_healthy |
| OID01259 | TCN2 | Olink CARDIOMETABOLIC | 0,106424779 | 0,011604247 | 0,03636795 | convalescent_vs_healthy |
| OID01290 | FCN2 | Olink CARDIOMETABOLIC | 0,185956123 | 0,01173464 | 0,036401332 | convalescent_vs_healthy |
| OID00331 | TMPRSS5 | Olink NEUROLOGY | 0,12891943 | 0,016885546 | 0,051850566 | convalescent_vs_healthy |
| OID00522 | HGF | Olink INFLAMMATION | -0,191871896 | 0,01733396 | 0,052695238 | convalescent_vs_healthy |
| OID00478 | IL7 | Olink INFLAMMATION | 0,265440914 | 0,017677159 | 0,052932316 | convalescent_vs_healthy |
| OID00562 | CSF-1 | Olink INFLAMMATION | -0,090016619 | 0,017760185 | 0,052932316 | convalescent_vs_healthy |
| OID00469 | PARP-1 | Olink CARDIOVASCULAR II | 0,662969771 | 0,017973391 | 0,053047678 | convalescent_vs_healthy |
| OID00420 | CD84 | Olink CARDIOVASCULAR II | 0,172521608 | 0,022409714 | 0,065331905 | convalescent_vs_healthy |
| OID00436 | CEACAM8 | Olink CARDIOVASCULAR II | -0,369833111 | 0,022627328 | 0,065331905 | convalescent_vs_healthy |
| OID00405 | LOX-1 | Olink CARDIOVASCULAR II | -0,38776773 | 0,022780204 | 0,065331905 | convalescent_vs_healthy |
| OID00498 | CCL4 | Olink INFLAMMATION | -0,231002835 | 0,023320146 | 0,066255369 | convalescent_vs_healthy |
| OID00542 | CD40 | Olink INFLAMMATION | 0,202590804 | 0,025971188 | 0,073104085 | convalescent_vs_healthy |
| OID00531 | CD5 | Olink INFLAMMATION | 0,138469188 | 0,026920452 | 0,075080894 | convalescent_vs_healthy |
| OID00369 | Dkk-4 | Olink NEUROLOGY | -0,142568938 | 0,028932053 | 0,079907896 | convalescent_vs_healthy |
| OID00344 | PVR | Olink NEUROLOGY | 0,109597147 | 0,029176896 | 0,079907896 | convalescent_vs_healthy |
| OID00326 | LAYN | Olink NEUROLOGY | -0,096725084 | 0,031484816 | 0,085458787 | convalescent_vs_healthy |
| OID00348 | CPM | Olink NEUROLOGY | 0,113136548 | 0,032954983 | 0,088657654 | convalescent_vs_healthy |
| OID01298 | TIMD4 | Olink CARDIOMETABOLIC | -0,124168001 | 0,03362704 | 0,089250002 | convalescent_vs_healthy |
| OID00447 | PRSS8 | Olink CARDIOVASCULAR II | -0,117330157 | 0,033813878 | 0,089250002 | convalescent_vs_healthy |
| OID00433 | XCL1 | Olink CARDIOVASCULAR II | 0,210501677 | 0,034055922 | 0,089250002 | convalescent_vs_healthy |
| OID00515 | IL-10RB | Olink INFLAMMATION | -0,092503275 | 0,03448583 | 0,089604209 | convalescent_vs_healthy |
| OID01245 | CDH1 | Olink CARDIOMETABOLIC | -0,088661445 | 0,036029408 | 0,092821527 | convalescent_vs_healthy |
| OID00445 | Dkk-1 | Olink CARDIOVASCULAR II | -0,202527792 | 0,036911698 | 0,09429543 | convalescent_vs_healthy |
| OID00374 | MANF | Olink NEUROLOGY | -0,142656066 | 0,039359329 | 0,099710301 | convalescent_vs_healthy |
| OID00471 | IL8 | Olink INFLAMMATION | -0,432344303 | 0,042967627 | 0,107951723 | convalescent_vs_healthy |
| OID00414 | CTRC | Olink CARDIOVASCULAR II | 0,180230124 | 0,043730575 | 0,108967989 | convalescent_vs_healthy |
| OID00371 | LAT | Olink NEUROLOGY | -0,421353422 | 0,047849021 | 0,118260996 | convalescent_vs_healthy |
| OID01268 | THBS4 | Olink CARDIOMETABOLIC | 0,153719473 | 0,04913575 | 0,12046184 | convalescent_vs_healthy |
| OID00552 | CX3CL1 | Olink INFLAMMATION | 0,115935908 | 0,058352301 | 0,140786503 | convalescent_vs_healthy |
| OID00432 | HO-1 | Olink CARDIOVASCULAR II | 0,126781015 | 0,05828128 | 0,140786503 | convalescent_vs_healthy |
| OID00391 | TNFRSF10A | Olink CARDIOVASCULAR II | -0,089120888 | 0,061010067 | 0,146039845 | convalescent_vs_healthy |
| OID01220 | LILRB5 | Olink CARDIOMETABOLIC | -0,192678474 | 0,061690473 | 0,146514873 | convalescent_vs_healthy |
| OID00536 | 4E-BP1 | Olink INFLAMMATION | 0,497109938 | 0,064413743 | 0,151796728 | convalescent_vs_healthy |
| OID00399 | TF | Olink CARDIOVASCULAR II | -0,072891073 | 0,066207639 | 0,154824017 | convalescent_vs_healthy |
| OID00484 | MCP-1 | Olink INFLAMMATION | 0,165942227 | 0,067927891 | 0,157634191 | convalescent_vs_healthy |
| OID00560 | ADA | Olink INFLAMMATION | 0,173764331 | 0,073100885 | 0,168353554 | convalescent_vs_healthy |
| OID01236 | PRSS2 | Olink CARDIOMETABOLIC | 0,106336343 | 0,075143626 | 0,171756858 | convalescent_vs_healthy |
| OID00347 | FLRT2 | Olink NEUROLOGY | -0,068321369 | 0,079888944 | 0,181026292 | convalescent_vs_healthy |
| OID00527 | MMP-10 | Olink INFLAMMATION | 0,168020073 | 0,080426384 | 0,181026292 | convalescent_vs_healthy |
| OID00302 | PRTG | Olink NEUROLOGY | 0,071603479 | 0,080985447 | 0,181026292 | convalescent_vs_healthy |
| OID00451 | FABP2 | Olink CARDIOVASCULAR II | -0,203147009 | 0,082017114 | 0,181994179 | convalescent_vs_healthy |
| OID00437 | PTX3 | Olink CARDIOVASCULAR II | 0,113215926 | 0,085579471 | 0,188522892 | convalescent_vs_healthy |
| OID00342 | SCARF2 | Olink NEUROLOGY | 0,071721298 | 0,09934297 | 0,217268077 | convalescent_vs_healthy |
| OID00365 | TNFRSF21 | Olink NEUROLOGY | 0,047462417 | 0,102649697 | 0,222707167 | convalescent_vs_healthy |
| OID01296 | LILRB2 | Olink CARDIOMETABOLIC | -0,083479785 | 0,103295101 | 0,222707167 | convalescent_vs_healthy |
| OID00358 | DDR1 | Olink NEUROLOGY | 0,051771863 | 0,10776909 | 0,230716925 | convalescent_vs_healthy |
| OID00359 | JAM-B | Olink NEUROLOGY | -0,080998659 | 0,11108754 | 0,236158128 | convalescent_vs_healthy |
| OID00334 | GM-CSF-R-alpha | Olink NEUROLOGY | 0,161184789 | 0,118253286 | 0,249645826 | convalescent_vs_healthy |
| OID00397 | PRSS27 | Olink CARDIOVASCULAR II | -0,104951881 | 0,120563619 | 0,252767864 | convalescent_vs_healthy |
| OID00549 | MCP-2 | Olink INFLAMMATION | 0,18307586 | 0,127970898 | 0,263761794 | convalescent_vs_healthy |
| OID00295 | CLM-6 | Olink NEUROLOGY | 0,056364422 | 0,129435522 | 0,263761794 | convalescent_vs_healthy |
| OID01294 | AOC3 | Olink CARDIOMETABOLIC | 0,063136822 | 0,129683863 | 0,263761794 | convalescent_vs_healthy |
| OID01293 | QPCT | Olink CARDIOMETABOLIC | 0,055251737 | 0,129701201 | 0,263761794 | convalescent_vs_healthy |
| OID01239 | F7 | Olink CARDIOMETABOLIC | 0,07234052 | 0,130145622 | 0,263761794 | convalescent_vs_healthy |
| OID00292 | UNC5C | Olink NEUROLOGY | -0,065449606 | 0,131692955 | 0,265130187 | convalescent_vs_healthy |
| OID00550 | CASP-8 | Olink INFLAMMATION | -0,349774858 | 0,133027085 | 0,266054169 | convalescent_vs_healthy |
| OID00307 | CPA2 | Olink NEUROLOGY | 0,110728552 | 0,139636848 | 0,277448377 | convalescent_vs_healthy |
| OID00290 | CADM3 | Olink NEUROLOGY | 0,084898607 | 0,142388745 | 0,281079081 | convalescent_vs_healthy |
| OID00477 | CD244 | Olink INFLAMMATION | 0,100771165 | 0,146147035 | 0,286636765 | convalescent_vs_healthy |
| OID01248 | CD59 | Olink CARDIOMETABOLIC | -0,044484247 | 0,147401062 | 0,287243095 | convalescent_vs_healthy |
| OID00328 | GDF-8 | Olink NEUROLOGY | 0,09503055 | 0,151828676 | 0,293986735 | convalescent_vs_healthy |
| OID00435 | SORT1 | Olink CARDIOVASCULAR II | -0,080327062 | 0,153855555 | 0,296025878 | convalescent_vs_healthy |
| OID01289 | PCOLCE | Olink CARDIOMETABOLIC | -0,069393621 | 0,156106868 | 0,298468477 | convalescent_vs_healthy |
| OID00449 | HB-EGF | Olink CARDIOVASCULAR II | -0,184745266 | 0,158502023 | 0,301153845 | convalescent_vs_healthy |
| OID00329 | THY 1 | Olink NEUROLOGY | 0,051644249 | 0,16365846 | 0,309019701 | convalescent_vs_healthy |
| OID01270 | TIE1 | Olink CARDIOMETABOLIC | -0,051006893 | 0,165314181 | 0,310219203 | convalescent_vs_healthy |
| OID00296 | EZR | Olink NEUROLOGY | -0,096122824 | 0,167130523 | 0,311703552 | convalescent_vs_healthy |
| OID00426 | KIM1 | Olink CARDIOVASCULAR II | 0,129726279 | 0,171130298 | 0,314991656 | convalescent_vs_healthy |
| OID01225 | CST3 | Olink CARDIOMETABOLIC | -0,065020099 | 0,171898429 | 0,314991656 | convalescent_vs_healthy |
| OID00411 | PIgR | Olink CARDIOVASCULAR II | 0,028722803 | 0,172354781 | 0,314991656 | convalescent_vs_healthy |
| OID01278 | LCN2 | Olink CARDIOMETABOLIC | 0,079693617 | 0,17303818 | 0,314991656 | convalescent_vs_healthy |
| OID00384 | PGF | Olink CARDIOVASCULAR II | -0,051161731 | 0,177188052 | 0,320625998 | convalescent_vs_healthy |
| OID00316 | CNTN5 | Olink NEUROLOGY | 0,08008013 | 0,189903956 | 0,341602382 | convalescent_vs_healthy |
| OID01217 | NRP1 | Olink CARDIOMETABOLIC | 0,034263825 | 0,19829965 | 0,353016716 | convalescent_vs_healthy |
| OID01295 | VASN | Olink CARDIOMETABOLIC | -0,043771534 | 0,198571903 | 0,353016716 | convalescent_vs_healthy |
| OID00450 | GDF-2 | Olink CARDIOVASCULAR II | -0,131087142 | 0,204388742 | 0,361245218 | convalescent_vs_healthy |
| OID01216 | CHL1 | Olink CARDIOMETABOLIC | 0,058801207 | 0,212803555 | 0,373943819 | convalescent_vs_healthy |
| OID01230 | ICAM1 | Olink CARDIOMETABOLIC | -0,059869911 | 0,215442407 | 0,376405125 | convalescent_vs_healthy |
| OID00352 | FcRL2 | Olink NEUROLOGY | -0,07811758 | 0,239451639 | 0,415961705 | convalescent_vs_healthy |
| OID00402 | IL-27 | Olink CARDIOVASCULAR II | -0,074280847 | 0,241083572 | 0,416417078 | convalescent_vs_healthy |
| OID01255 | IGFBP3 | Olink CARDIOMETABOLIC | -0,065206487 | 0,243969992 | 0,418367241 | convalescent_vs_healthy |
| OID01243 | MBL2 | Olink CARDIOMETABOLIC | 0,19326 | 0,244965029 | 0,418367241 | convalescent_vs_healthy |
| OID00476 | CDCP1 | Olink INFLAMMATION | -0,086857714 | 0,248426194 | 0,421621762 | convalescent_vs_healthy |
| OID00521 | TRANCE | Olink INFLAMMATION | -0,106451824 | 0,250201635 | 0,421621762 | convalescent_vs_healthy |
| OID01257 | VCAM1 | Olink CARDIOMETABOLIC | -0,049249338 | 0,251031378 | 0,421621762 | convalescent_vs_healthy |
| OID01260 | TNXB | Olink CARDIOMETABOLIC | -0,041197455 | 0,252616639 | 0,421953067 | convalescent_vs_healthy |
| OID01249 | SELL | Olink CARDIOMETABOLIC | 0,049075165 | 0,25485144 | 0,423359769 | convalescent_vs_healthy |
| OID00386 | BOC | Olink CARDIOVASCULAR II | 0,047412064 | 0,259225466 | 0,428285552 | convalescent_vs_healthy |
| OID00353 | MDGA1 | Olink NEUROLOGY | 0,102992042 | 0,276792934 | 0,450621731 | convalescent_vs_healthy |
| OID00317 | ADAM 22 | Olink NEUROLOGY | -0,061399781 | 0,277582548 | 0,450621731 | convalescent_vs_healthy |
| OID00309 | SMPD1 | Olink NEUROLOGY | 0,079101794 | 0,279202809 | 0,450621731 | convalescent_vs_healthy |
| OID00499 | CD6 | Olink INFLAMMATION | 0,113990823 | 0,280648241 | 0,450621731 | convalescent_vs_healthy |
| OID00306 | PLXNB3 | Olink NEUROLOGY | 0,120804032 | 0,281593751 | 0,450621731 | convalescent_vs_healthy |
| OID00332 | CDH3 | Olink NEUROLOGY | 0,056881724 | 0,281638582 | 0,450621731 | convalescent_vs_healthy |
| OID00412 | RAGE | Olink CARDIOVASCULAR II | -0,054300957 | 0,288013448 | 0,45752572 | convalescent_vs_healthy |
| OID00370 | EDA2R | Olink NEUROLOGY | 0,05806443 | 0,288963613 | 0,45752572 | convalescent_vs_healthy |
| OID00337 | CD200 | Olink NEUROLOGY | 0,044255548 | 0,293630978 | 0,462506825 | convalescent_vs_healthy |
| OID00491 | CST5 | Olink INFLAMMATION | 0,07037792 | 0,301706478 | 0,469682591 | convalescent_vs_healthy |
| OID01238 | MET | Olink CARDIOMETABOLIC | 0,040978772 | 0,302475422 | 0,469682591 | convalescent_vs_healthy |
| OID01226 | ANG | Olink CARDIOMETABOLIC | -0,056526799 | 0,302821671 | 0,469682591 | convalescent_vs_healthy |
| OID01253 | IL7R | Olink CARDIOMETABOLIC | -0,078406573 | 0,306486215 | 0,471041076 | convalescent_vs_healthy |
| OID01307 | LYVE1 | Olink CARDIOMETABOLIC | -0,049381084 | 0,307880142 | 0,471041076 | convalescent_vs_healthy |
| OID00401 | PDGF subunit B | Olink CARDIOVASCULAR II | -0,071690148 | 0,308345967 | 0,471041076 | convalescent_vs_healthy |
| OID00533 | Flt3L | Olink INFLAMMATION | -0,056386653 | 0,319941583 | 0,486311206 | convalescent_vs_healthy |
| OID00303 | ROBO2 | Olink NEUROLOGY | 0,043071056 | 0,322606354 | 0,487922048 | convalescent_vs_healthy |
| OID01286 | GAS6 | Olink CARDIOMETABOLIC | 0,042419578 | 0,335879663 | 0,502992205 | convalescent_vs_healthy |
| OID00444 | DCN | Olink CARDIOVASCULAR II | 0,059258904 | 0,33547002 | 0,502992205 | convalescent_vs_healthy |
| OID00439 | CCL17 | Olink CARDIOVASCULAR II | 0,138503134 | 0,352268985 | 0,52494986 | convalescent_vs_healthy |
| OID01273 | NOTCH1 | Olink CARDIOMETABOLIC | 0,026699108 | 0,37968913 | 0,561556612 | convalescent_vs_healthy |
| OID01241 | KIT | Olink CARDIOMETABOLIC | 0,039144287 | 0,380528494 | 0,561556612 | convalescent_vs_healthy |
| OID01292 | CCL14 | Olink CARDIOMETABOLIC | -0,041564799 | 0,392573647 | 0,576533279 | convalescent_vs_healthy |
| OID00298 | NBL1 | Olink NEUROLOGY | -0,020016423 | 0,398913411 | 0,583027293 | convalescent_vs_healthy |
| OID00454 | GT | Olink CARDIOVASCULAR II | 0,06986175 | 0,406223981 | 0,590871246 | convalescent_vs_healthy |
| OID00373 | LAIR-2 | Olink NEUROLOGY | -0,132637627 | 0,408729118 | 0,591684056 | convalescent_vs_healthy |
| OID00442 | IgG Fc receptor II-b | Olink CARDIOVASCULAR II | 0,078972564 | 0,411080564 | 0,592267732 | convalescent_vs_healthy |
| OID00355 | PDGF-R-alpha | Olink NEUROLOGY | -0,034199232 | 0,422751053 | 0,606209057 | convalescent_vs_healthy |
| OID00505 | CCL11 | Olink INFLAMMATION | 0,064191604 | 0,428408961 | 0,606283095 | convalescent_vs_healthy |
| OID00375 | TN-R | Olink NEUROLOGY | 0,054128951 | 0,426200644 | 0,606283095 | convalescent_vs_healthy |
| OID01234 | GP1BA | Olink CARDIOMETABOLIC | 0,072523532 | 0,428785741 | 0,606283095 | convalescent_vs_healthy |
| OID00315 | SIGLEC1 | Olink NEUROLOGY | -0,048892641 | 0,436882743 | 0,61257871 | convalescent_vs_healthy |
| OID01264 | IGFBP6 | Olink CARDIOMETABOLIC | 0,032881339 | 0,437268356 | 0,61257871 | convalescent_vs_healthy |
| OID00377 | Nr-CAM | Olink NEUROLOGY | 0,020207848 | 0,439833098 | 0,613345237 | convalescent_vs_healthy |
| OID01291 | TGFBI | Olink CARDIOMETABOLIC | 0,04194454 | 0,444837199 | 0,616716454 | convalescent_vs_healthy |
| OID00330 | WFIKKN1 | Olink NEUROLOGY | 0,047019597 | 0,44757433 | 0,616716454 | convalescent_vs_healthy |
| OID01219 | FCGR3B | Olink CARDIOMETABOLIC | -0,049212853 | 0,448336633 | 0,616716454 | convalescent_vs_healthy |
| OID00407 | GIF | Olink CARDIOVASCULAR II | 0,080516044 | 0,453647173 | 0,621210543 | convalescent_vs_healthy |
| OID01251 | CD46 | Olink CARDIOMETABOLIC | 0,052441128 | 0,456210588 | 0,621919367 | convalescent_vs_healthy |
| OID01228 | PROC | Olink CARDIOMETABOLIC | 0,04023179 | 0,462268517 | 0,627364416 | convalescent_vs_healthy |
| OID00506 | TNFSF14 | Olink INFLAMMATION | -0,10580425 | 0,470886034 | 0,636219353 | convalescent_vs_healthy |
| OID01224 | TIMP1 | Olink CARDIOMETABOLIC | -0,033689692 | 0,476571246 | 0,640064496 | convalescent_vs_healthy |
| OID01218 | PLXNB2 | Olink CARDIOMETABOLIC | -0,023563391 | 0,477942897 | 0,640064496 | convalescent_vs_healthy |
| OID00518 | PD-L1 | Olink INFLAMMATION | 0,038629229 | 0,488681774 | 0,646646646 | convalescent_vs_healthy |
| OID00413 | SOD2 | Olink CARDIOVASCULAR II | 0,016300545 | 0,485386631 | 0,646646646 | convalescent_vs_healthy |
| OID01284 | PTPRS | Olink CARDIOMETABOLIC | -0,022489734 | 0,489239239 | 0,646646646 | convalescent_vs_healthy |
| OID01247 | NCAM1 | Olink CARDIOMETABOLIC | -0,02615575 | 0,495297844 | 0,651820539 | convalescent_vs_healthy |
| OID01266 | DPP4 | Olink CARDIOMETABOLIC | -0,030355031 | 0,498137132 | 0,652731414 | convalescent_vs_healthy |
| OID00288 | NRP2 | Olink NEUROLOGY | 0,014984089 | 0,510506511 | 0,666068581 | convalescent_vs_healthy |
| OID00422 | SERPINA12 | Olink CARDIOVASCULAR II | 0,106996636 | 0,519284128 | 0,674625534 | convalescent_vs_healthy |
| OID00486 | CXCL11 | Olink INFLAMMATION | 0,082867354 | 0,541684007 | 0,700731652 | convalescent_vs_healthy |
| OID00512 | FGF-21 | Olink INFLAMMATION | 0,105936813 | 0,553509152 | 0,71299484 | convalescent_vs_healthy |
| OID00427 | THBS2 | Olink CARDIOVASCULAR II | -0,016388839 | 0,561110826 | 0,719737094 | convalescent_vs_healthy |
| OID00346 | SKR3 | Olink NEUROLOGY | -0,020118384 | 0,569939129 | 0,727989475 | convalescent_vs_healthy |
| OID00338 | NTRK2 | Olink NEUROLOGY | -0,017545098 | 0,572486141 | 0,728183208 | convalescent_vs_healthy |
| OID00504 | MCP-4 | Olink INFLAMMATION | 0,063322788 | 0,582751289 | 0,732051206 | convalescent_vs_healthy |
| OID00363 | N2DL-2 | Olink NEUROLOGY | -0,031141388 | 0,581476191 | 0,732051206 | convalescent_vs_healthy |
| OID00314 | RGMB | Olink NEUROLOGY | 0,025095157 | 0,581668104 | 0,732051206 | convalescent_vs_healthy |
| OID00460 | hOSCAR | Olink CARDIOVASCULAR II | -0,017226376 | 0,587514405 | 0,734997444 | convalescent_vs_healthy |
| OID01305 | FETUB | Olink CARDIOMETABOLIC | -0,038283261 | 0,591394292 | 0,735168951 | convalescent_vs_healthy |
| OID00380 | ANGPT1 | Olink CARDIOVASCULAR II | -0,066316915 | 0,592488135 | 0,735168951 | convalescent_vs_healthy |
| OID00553 | TNFRSF9 | Olink INFLAMMATION | -0,029293084 | 0,611103328 | 0,749094402 | convalescent_vs_healthy |
| OID01254 | ENG | Olink CARDIOMETABOLIC | 0,017419766 | 0,607552047 | 0,748130168 | convalescent_vs_healthy |
| OID01267 | ICAM3 | Olink CARDIOMETABOLIC | -0,019318464 | 0,607855762 | 0,748130168 | convalescent_vs_healthy |
| OID00517 | IL-18R1 | Olink INFLAMMATION | -0,030033525 | 0,625033645 | 0,758700939 | convalescent_vs_healthy |
| OID00322 | HAGH | Olink NEUROLOGY | 0,090997508 | 0,628031747 | 0,758700939 | convalescent_vs_healthy |
| OID00428 | TM | Olink CARDIOVASCULAR II | -0,022452051 | 0,626292768 | 0,758700939 | convalescent_vs_healthy |
| OID01258 | CR2 | Olink CARDIOMETABOLIC | -0,034395174 | 0,628923146 | 0,758700939 | convalescent_vs_healthy |
| OID01244 | FCGR2A | Olink CARDIOMETABOLIC | -0,035294925 | 0,646536341 | 0,774924964 | convalescent_vs_healthy |
| OID01246 | CCL5 | Olink CARDIOMETABOLIC | 0,06979577 | 0,652189256 | 0,774924964 | convalescent_vs_healthy |
| OID00408 | SCF | Olink CARDIOVASCULAR II | -0,023929974 | 0,652662668 | 0,774924964 | convalescent_vs_healthy |
| OID00395 | PAR-1 | Olink CARDIOVASCULAR II | 0,048717237 | 0,652841514 | 0,774924964 | convalescent_vs_healthy |
| OID00313 | EPHB6 | Olink NEUROLOGY | -0,018431903 | 0,655117486 | 0,774924964 | convalescent_vs_healthy |
| OID00310 | MSR1 | Olink NEUROLOGY | -0,027329448 | 0,669085026 | 0,788379256 | convalescent_vs_healthy |
| OID00388 | SRC | Olink CARDIOVASCULAR II | 0,047416567 | 0,682328521 | 0,800626795 | convalescent_vs_healthy |
| OID01231 | REG1A | Olink CARDIOMETABOLIC | -0,02663241 | 0,684746601 | 0,800626795 | convalescent_vs_healthy |
| OID00318 | CLEC1B | Olink NEUROLOGY | 0,056528659 | 0,691093229 | 0,8049515 | convalescent_vs_healthy |
| OID00458 | PD-L2 | Olink CARDIOVASCULAR II | 0,035824876 | 0,705671173 | 0,818794032 | convalescent_vs_healthy |
| OID01213 | DNER | Olink INFLAMMATION | 0,015311833 | 0,715148343 | 0,826635347 | convalescent_vs_healthy |
| OID00561 | TNFB | Olink INFLAMMATION | 0,020869718 | 0,738628706 | 0,850542147 | convalescent_vs_healthy |
| OID00551 | CCL25 | Olink INFLAMMATION | -0,025785983 | 0,744859356 | 0,85227041 | convalescent_vs_healthy |
| OID01302 | CFHR5 | Olink CARDIOMETABOLIC | -0,017472244 | 0,745968148 | 0,85227041 | convalescent_vs_healthy |
| OID00361 | N-CDase | Olink NEUROLOGY | 0,028571209 | 0,74854013 | 0,85227041 | convalescent_vs_healthy |
| OID00333 | GFR-alpha-1 | Olink NEUROLOGY | -0,013592716 | 0,772030671 | 0,875736283 | convalescent_vs_healthy |
| OID00457 | ACE2 | Olink CARDIOVASCULAR II | 0,019278139 | 0,777779846 | 0,878977967 | convalescent_vs_healthy |
| OID00429 | VSIG2 | Olink CARDIOVASCULAR II | -0,016425317 | 0,787306786 | 0,886449122 | convalescent_vs_healthy |
| OID00472 | VEGFA | Olink INFLAMMATION | -0,021723014 | 0,811675083 | 0,905725939 | convalescent_vs_healthy |
| OID00511 | LIF-R | Olink INFLAMMATION | 0,009118357 | 0,816183888 | 0,905725939 | convalescent_vs_healthy |
| OID01232 | SERPINA7 | Olink CARDIOMETABOLIC | -0,013030797 | 0,805596029 | 0,903694438 | convalescent_vs_healthy |
| OID00500 | SCF | Olink INFLAMMATION | -0,013464045 | 0,819975841 | 0,905725939 | convalescent_vs_healthy |
| OID00294 | Siglec-9 | Olink NEUROLOGY | 0,011322592 | 0,822303813 | 0,905725939 | convalescent_vs_healthy |
| OID00423 | REN | Olink CARDIOVASCULAR II | 0,0201786 | 0,821221508 | 0,905725939 | convalescent_vs_healthy |
| OID01262 | GNLY | Olink CARDIOMETABOLIC | -0,012462924 | 0,831296094 | 0,910229311 | convalescent_vs_healthy |
| OID01261 | CA4 | Olink CARDIOMETABOLIC | -0,00791249 | 0,832380751 | 0,910229311 | convalescent_vs_healthy |
| OID01274 | COMP | Olink CARDIOMETABOLIC | 0,012074615 | 0,836934665 | 0,911928811 | convalescent_vs_healthy |
| OID00368 | IL12 | Olink NEUROLOGY | 0,013784431 | 0,866371047 | 0,940631422 | convalescent_vs_healthy |
| OID01303 | MEGF9 | Olink CARDIOMETABOLIC | 0,007385595 | 0,873929749 | 0,945461366 | convalescent_vs_healthy |
| OID00556 | CCL20 | Olink INFLAMMATION | 0,017087739 | 0,904586832 | 0,946970466 | convalescent_vs_healthy |
| OID00438 | PSGL-1 | Olink CARDIOVASCULAR II | 0,005207661 | 0,878638124 | 0,946692173 | convalescent_vs_healthy |
| OID01271 | COL18A1 | Olink CARDIOMETABOLIC | -0,006401394 | 0,883909251 | 0,946692173 | convalescent_vs_healthy |
| OID01250 | NID1 | Olink CARDIOMETABOLIC | -0,008864041 | 0,884409793 | 0,946692173 | convalescent_vs_healthy |
| OID00479 | OPG | Olink INFLAMMATION | -0,005513547 | 0,917553284 | 0,946970466 | convalescent_vs_healthy |
| OID00523 | IL-12B | Olink INFLAMMATION | 0,008352319 | 0,918273647 | 0,946970466 | convalescent_vs_healthy |
| OID00345 | TNFRSF12A | Olink NEUROLOGY | 0,006594484 | 0,898391165 | 0,946970466 | convalescent_vs_healthy |
| OID00470 | HAOX1 | Olink CARDIOVASCULAR II | 0,026615204 | 0,890558812 | 0,946970466 | convalescent_vs_healthy |
| OID01306 | ANGPTL3 | Olink CARDIOMETABOLIC | -0,007136567 | 0,897720263 | 0,946970466 | convalescent_vs_healthy |
| OID01240 | IGLC2 | Olink CARDIOMETABOLIC | -0,008373215 | 0,904736376 | 0,946970466 | convalescent_vs_healthy |
| OID00461 | TNFRSF13B | Olink CARDIOVASCULAR II | -0,006950606 | 0,900968705 | 0,946970466 | convalescent_vs_healthy |
| OID01297 | LILRB1 | Olink CARDIOMETABOLIC | -0,00416276 | 0,913090693 | 0,946970466 | convalescent_vs_healthy |
| OID01252 | ST6GAL1 | Olink CARDIOMETABOLIC | -0,00485964 | 0,918935156 | 0,946970466 | convalescent_vs_healthy |
| OID00410 | FGF-21 | Olink CARDIOVASCULAR II | 0,021166577 | 0,912133028 | 0,946970466 | convalescent_vs_healthy |
| OID00351 | BMP-4 | Olink NEUROLOGY | 0,009021564 | 0,926692832 | 0,951585158 | convalescent_vs_healthy |
| OID00545 | FGF-19 | Olink INFLAMMATION | 0,004967397 | 0,972569393 | 0,975779193 | convalescent_vs_healthy |
| OID00366 | CLM-1 | Olink NEUROLOGY | -0,009691766 | 0,929673658 | 0,951585158 | convalescent_vs_healthy |
| OID05547 | IFN-gamma | Olink INFLAMMATION | -0,001259903 | 0,991881886 | 0,991881886 | convalescent_vs_healthy |
| OID00305 | RGMA | Olink NEUROLOGY | 0,002710778 | 0,947778458 | 0,966861246 | convalescent_vs_healthy |
| OID00287 | NMNAT1 | Olink NEUROLOGY | 0,015008502 | 0,960271091 | 0,973074706 | convalescent_vs_healthy |
| OID01227 | F11 | Olink CARDIOMETABOLIC | -0,00150483 | 0,969810386 | 0,975779193 | convalescent_vs_healthy |
| OID00400 | IL1RL2 | Olink CARDIOVASCULAR II | -0,003321493 | 0,956468282 | 0,972462735 | convalescent_vs_healthy |
| OID00416 | SPON2 | Olink CARDIOVASCULAR II | 0,001430195 | 0,966518604 | 0,975779193 | convalescent_vs_healthy |

**Table S6: Overlap between differentially abundant proteins in ICU, non-ICU and post-COVID-19 compared to healthy controls**

|  | **Group** | **Proteins** |
| --- | --- | --- |
| **Up** | ICU | GNLY, ANGPTL3, REG1A, NRP1, CA3, COL18A1, IL1RL2, IL18, OSM, CCL4, IL18, MMP-10 |
|  | non-ICU | CA1, F11, MEGF9, ANG, CCL18, AMBP, IL7, LIF-R, CCL25 |
|  | post-COVID-19 | PRCP, CRTAC1, APOM, CXCL1, VEGFD, LAP TGF-beta-1, TRAIL, CXCL1, CXCL5, CXCL6, ST1A1 |
|  | ICU / non-ICU | ICAM1,TGFBI,CST3,LILRB1,MET,VCAM1,MBL2,TIMP1,TNC,NID1,CFHR5,PLXNB2,ST6GAL1,QPCT,PRSS2,CNDP1,TIMD4,LILRB2,C1QTNF1,GAS6,IGLC2,PGF,IL-1ra,TNFRSF10A,TNFRSF11A,TRAIL-R2,IL-27,Gal-9,FGF-21,PIgR,RAGE,SPON2,REN,KIM1,THBS2,HO-1,XCL1,PTX3,CCL3,DCN,GT,ACE2,CTSL1,hOSCAR,HAOX1,IL8,VEGFA,CDCP1,OPG,MCP-1,CXCL11,CXCL9,TGF-alpha,TNFSF14,FGF-21,IL-18R1,PD-L1,HGF,IL-12B,CCL3,CXCL10,4E-BP1,CD40,IFN-gamma,MCP-2,CX3CL1,CCL20,ADA,CSF-1 |
|  | ICU / post-COVID-19 | CD40-L, STK4, MMP12, NEMO |
|  | non-ICU / post-COVID-19 | PAM, SPARCL1 |
|  | ICU /non-ICU / post-COVID-19 | FCN2, TCN2, EFEMP1, BMP-6, ADM, IDUA, GH, FS, GLO1, MERTK, PRELP, MMP7, ITGB1BP2, LPL, AGRP, THPO, MARCO, CD4, CD8A, uPA, AXIN1, MMP-1, CCL19, TNF, CCL23, TWEAK, STAMBP |
|  |  |  |
| **Down** | ICU | IGFBP6, NOTCH1, PROC, FETUB, CTRC, FABP2, VEGFD, TRAIL, CD5, Flt3L, TNFB |
|  | non-ICU | CCL5, CD46, FCGR2A, TIE1, GP1BA, LYVE1, ANGPT1, LOX-1, CEACAM8, LAP TGF-beta-1, FGF-19 |
|  | post-COVID-19 | CA1, TNC, CNDP1, CA3, OSMR, CCL18, C1QTNF1, IL-1ra, TNFRSF11A, TRAIL-R2, Gal-9, IL18, AMBP, CCL3, CTSL1, LEP, CXCL9, OSM, IL18, TGF-alpha, CCL3, CXCL10 |
|  | ICU / non-ICU | CHL1, ENG, IGFBP3, CR2, CDH1, NCAM1, PCOLCE, IL7R, COMP, PTPRS, DPP4, THBS4, APOM, KIT, AOC3, LILRB5, BOC, PRSS27, PDGF subunit B, GIF, SCF, SORT1, PSGL-1, CCL17, Dkk-1, HB-EGF, GDF-2, CD244, CST5, CD6, SCF, MCP-4, CXCL5, TRANCE, DNER, CASP-8 |
|  | ICU / post-COVID-19 | IL16, TGM2 |
|  | non-ICU / post-COVID-19 | HSP 27, EN-RAGE |
|  | ICU /non-ICU / post-COVID-19 | SERPINA5, SAA4, C2, ADAM-TS13, TIE2, CCL28 |

**Table S7: Clustering and summary of differential abundance results of ICU, non-ICU and post-COVID-19 compared to healthy controls**

| **protein** | **Cluster (Fig. 2D)** | **Sig (ICU)** | **Sig (nonICU)** | **Sig (Postcovid)** |
| --- | --- | --- | --- | --- |
| TGFBI | 1 | TRUE | TRUE | FALSE |
| MEGF9 | 1 | FALSE | TRUE | FALSE |
| FCN2 | 1 | TRUE | TRUE | TRUE |
| ANG | 1 | FALSE | TRUE | FALSE |
| F11 | 1 | FALSE | TRUE | FALSE |
| LEP | 1 | FALSE | FALSE | TRUE |
| IL1RL2 | 1 | TRUE | FALSE | FALSE |
| THPO | 1 | TRUE | TRUE | TRUE |
| MBL2 | 1 | TRUE | TRUE | FALSE |
| DCN | 1 | TRUE | TRUE | FALSE |
| CA3 | 1 | TRUE | FALSE | TRUE |
| PRSS2 | 1 | TRUE | TRUE | FALSE |
| REG1A | 1 | TRUE | FALSE | FALSE |
| MMP-10 | 1 | TRUE | FALSE | FALSE |
| MMP12 | 1 | TRUE | FALSE | TRUE |
| HAOX1 | 1 | TRUE | TRUE | FALSE |
| C1QTNF1 | 1 | TRUE | TRUE | TRUE |
| CFHR5 | 1 | TRUE | TRUE | FALSE |
| CNDP1 | 1 | TRUE | TRUE | TRUE |
| THBS2 | 1 | TRUE | TRUE | FALSE |
| PIgR | 1 | TRUE | TRUE | FALSE |
| hOSCAR | 1 | TRUE | TRUE | FALSE |
| PGF | 1 | TRUE | TRUE | FALSE |
| FS | 1 | TRUE | TRUE | TRUE |
| AGRP | 1 | TRUE | TRUE | TRUE |
| HO-1 | 1 | TRUE | TRUE | FALSE |
| MERTK | 1 | TRUE | TRUE | TRUE |
| CD8A | 1 | TRUE | TRUE | TRUE |
| XCL1 | 1 | TRUE | TRUE | FALSE |
| IDUA | 1 | TRUE | TRUE | TRUE |
| MARCO | 1 | TRUE | TRUE | TRUE |
| CD4 | 1 | TRUE | TRUE | TRUE |
| PRELP | 1 | TRUE | TRUE | TRUE |
| MMP7 | 1 | TRUE | TRUE | TRUE |
| TNF | 1 | TRUE | TRUE | TRUE |
| ADM | 1 | TRUE | TRUE | TRUE |
| EFEMP1 | 1 | TRUE | TRUE | TRUE |
| PLXNB2 | 1 | TRUE | TRUE | FALSE |
| uPA | 1 | TRUE | TRUE | TRUE |
| ICAM1 | 1 | TRUE | TRUE | FALSE |
| LILRB1 | 1 | TRUE | TRUE | FALSE |
| LILRB2 | 1 | TRUE | TRUE | FALSE |
| REN | 1 | TRUE | TRUE | FALSE |
| CD40 | 1 | TRUE | TRUE | FALSE |
| NID1 | 1 | TRUE | TRUE | FALSE |
| ST6GAL1 | 1 | TRUE | TRUE | FALSE |
| CXCL11 | 1 | TRUE | TRUE | FALSE |
| VEGFA | 1 | TRUE | TRUE | FALSE |
| IGLC2 | 1 | TRUE | TRUE | FALSE |
| CCL23 | 1 | TRUE | TRUE | TRUE |
| TNC | 1 | TRUE | TRUE | TRUE |
| ACE2 | 1 | TRUE | TRUE | FALSE |
| KIM1 | 1 | TRUE | TRUE | FALSE |
| SPON2 | 1 | TRUE | TRUE | FALSE |
| FGF-21 | 1 | TRUE | TRUE | FALSE |
| FGF-21 | 1 | TRUE | TRUE | FALSE |
| CCL19 | 1 | TRUE | TRUE | TRUE |
| CCL20 | 1 | TRUE | TRUE | FALSE |
| CXCL9 | 1 | TRUE | TRUE | TRUE |
| OPG | 1 | TRUE | TRUE | FALSE |
| HGF | 1 | TRUE | TRUE | FALSE |
| TIMP1 | 1 | TRUE | TRUE | FALSE |
| TRAIL-R2 | 1 | TRUE | TRUE | TRUE |
| MCP-1 | 1 | TRUE | TRUE | FALSE |
| CTSL1 | 1 | TRUE | TRUE | TRUE |
| CDCP1 | 1 | TRUE | TRUE | FALSE |
| IL-18R1 | 1 | TRUE | TRUE | FALSE |
| PD-L1 | 1 | TRUE | TRUE | FALSE |
| TNFRSF10A | 1 | TRUE | TRUE | FALSE |
| CSF-1 | 1 | TRUE | TRUE | FALSE |
| Gal-9 | 1 | TRUE | TRUE | TRUE |
| TIMD4 | 1 | TRUE | TRUE | FALSE |
| CCL18 | 1 | FALSE | TRUE | TRUE |
| QPCT | 1 | TRUE | TRUE | FALSE |
| COL18A1 | 1 | TRUE | FALSE | FALSE |
| TNFRSF11A | 1 | TRUE | TRUE | TRUE |
| CST3 | 1 | TRUE | TRUE | FALSE |
| LIF-R | 1 | FALSE | TRUE | FALSE |
| MCP-2 | 1 | TRUE | TRUE | FALSE |
| RAGE | 1 | TRUE | TRUE | FALSE |
| TCN2 | 1 | TRUE | TRUE | TRUE |
| CXCL10 | 1 | TRUE | TRUE | TRUE |
| IFN-gamma | 1 | TRUE | TRUE | FALSE |
| CX3CL1 | 1 | TRUE | TRUE | FALSE |
| GAS6 | 1 | TRUE | TRUE | FALSE |
| VCAM1 | 1 | TRUE | TRUE | FALSE |
| PTX3 | 1 | TRUE | TRUE | FALSE |
| IL-27 | 1 | TRUE | TRUE | FALSE |
| IL-12B | 1 | TRUE | TRUE | FALSE |
| AMBP | 1 | FALSE | TRUE | TRUE |
| IL18 | 1 | TRUE | FALSE | TRUE |
| IL18 | 1 | TRUE | FALSE | TRUE |
| IL-1ra | 1 | TRUE | TRUE | TRUE |
| TGF-alpha | 1 | TRUE | TRUE | TRUE |
| OSM | 1 | TRUE | FALSE | TRUE |
| CCL4 | 1 | TRUE | FALSE | FALSE |
| CCL3 | 1 | TRUE | TRUE | TRUE |
| CCL3 | 1 | TRUE | TRUE | TRUE |
| TNFSF14 | 1 | TRUE | TRUE | FALSE |
| IL8 | 1 | TRUE | TRUE | FALSE |
| TGM2 | 1 | TRUE | FALSE | TRUE |
| CA1 | 1 | FALSE | TRUE | TRUE |
| ADA | 1 | TRUE | TRUE | FALSE |
| GLO1 | 1 | TRUE | TRUE | TRUE |
| 4E-BP1 | 1 | TRUE | TRUE | FALSE |
|  |  |  |  |  |
| CCL25 | 2 | FALSE | TRUE | FALSE |
| GT | 2 | TRUE | TRUE | FALSE |
| FABP2 | 2 | TRUE | FALSE | FALSE |
| MMP-1 | 2 | TRUE | TRUE | TRUE |
| TWEAK | 2 | TRUE | TRUE | TRUE |
| SPARCL1 | 2 | FALSE | TRUE | TRUE |
| BMP-6 | 2 | TRUE | TRUE | TRUE |
| LPL | 2 | TRUE | TRUE | TRUE |
| GH | 2 | TRUE | TRUE | TRUE |
| VEGFD | 2 | TRUE | FALSE | TRUE |
|  |  |  |  |  |
| IL7 | 3 | FALSE | TRUE | FALSE |
| CXCL6 | 3 | FALSE | FALSE | TRUE |
| CXCL5 | 3 | TRUE | TRUE | TRUE |
| CXCL1 | 3 | FALSE | FALSE | TRUE |
| CXCL1 | 3 | FALSE | FALSE | TRUE |
| CCL17 | 3 | TRUE | TRUE | FALSE |
| MCP-4 | 3 | TRUE | TRUE | FALSE |
| CD40-L | 3 | TRUE | FALSE | TRUE |
| LAP TGF-beta-1 | 3 | FALSE | TRUE | TRUE |
| SORT1 | 3 | TRUE | TRUE | FALSE |
| HB-EGF | 3 | TRUE | TRUE | FALSE |
| Dkk-1 | 3 | TRUE | TRUE | FALSE |
| CCL5 | 3 | FALSE | TRUE | FALSE |
| PDGF subunit B | 3 | TRUE | TRUE | FALSE |
| ANGPT1 | 3 | FALSE | TRUE | FALSE |
| STAMBP | 3 | TRUE | TRUE | TRUE |
| ITGB1BP2 | 3 | TRUE | TRUE | TRUE |
| AXIN1 | 3 | TRUE | TRUE | TRUE |
| STK4 | 3 | TRUE | FALSE | TRUE |
| NEMO | 3 | TRUE | FALSE | TRUE |
| CD244 | 3 | TRUE | TRUE | FALSE |
| GP1BA | 3 | FALSE | TRUE | FALSE |
| CD46 | 3 | FALSE | TRUE | FALSE |
| FGF-19 | 3 | FALSE | TRUE | FALSE |
| CCL28 | 3 | TRUE | TRUE | TRUE |
| Flt3L | 3 | TRUE | FALSE | FALSE |
| CST5 | 3 | TRUE | TRUE | FALSE |
| PROC | 3 | TRUE | FALSE | FALSE |
| IGFBP3 | 3 | TRUE | TRUE | FALSE |
| FETUB | 3 | TRUE | FALSE | FALSE |
| PSGL-1 | 3 | TRUE | TRUE | FALSE |
| CD6 | 3 | TRUE | TRUE | FALSE |
| CD5 | 3 | TRUE | FALSE | FALSE |
| IL7R | 3 | TRUE | TRUE | FALSE |
| TIE2 | 3 | TRUE | TRUE | TRUE |
| CHL1 | 3 | TRUE | TRUE | FALSE |
| AOC3 | 3 | TRUE | TRUE | FALSE |
| NCAM1 | 3 | TRUE | TRUE | FALSE |
| ENG | 3 | TRUE | TRUE | FALSE |
| NOTCH1 | 3 | TRUE | FALSE | FALSE |
| LILRB5 | 3 | TRUE | TRUE | FALSE |
| GIF | 3 | TRUE | TRUE | FALSE |
| PCOLCE | 3 | TRUE | TRUE | FALSE |
| PTPRS | 3 | TRUE | TRUE | FALSE |
| COMP | 3 | TRUE | TRUE | FALSE |
| THBS4 | 3 | TRUE | TRUE | FALSE |
| TRANCE | 3 | TRUE | TRUE | FALSE |
| CR2 | 3 | TRUE | TRUE | FALSE |
| KIT | 3 | TRUE | TRUE | FALSE |
| DNER | 3 | TRUE | TRUE | FALSE |
| DPP4 | 3 | TRUE | TRUE | FALSE |
| APOM | 3 | TRUE | TRUE | TRUE |
| SCF | 3 | TRUE | TRUE | FALSE |
| SCF | 3 | TRUE | TRUE | FALSE |
| CASP-8 | 3 | TRUE | TRUE | FALSE |
| PRSS27 | 3 | TRUE | TRUE | FALSE |
| BOC | 3 | TRUE | TRUE | FALSE |
| SERPINA5 | 3 | TRUE | TRUE | TRUE |
| GDF-2 | 3 | TRUE | TRUE | FALSE |
| CTRC | 3 | TRUE | FALSE | FALSE |
| TNFB | 3 | TRUE | FALSE | FALSE |
| TRAIL | 3 | TRUE | FALSE | TRUE |
|  |  |  |  |  |
| NRP1 | 4 | TRUE | FALSE | FALSE |
| TIE1 | 4 | FALSE | TRUE | FALSE |
| ANGPTL3 | 4 | TRUE | FALSE | FALSE |
| OSMR | 4 | FALSE | FALSE | TRUE |
| GNLY | 4 | TRUE | FALSE | FALSE |
| MET | 4 | TRUE | TRUE | FALSE |
| LYVE1 | 4 | FALSE | TRUE | FALSE |
| FCGR2A | 4 | FALSE | TRUE | FALSE |
| PAM | 4 | FALSE | TRUE | TRUE |
| IGFBP6 | 4 | TRUE | FALSE | FALSE |
| CDH1 | 4 | TRUE | TRUE | FALSE |
| ADAM-TS13 | 4 | TRUE | TRUE | TRUE |
| SAA4 | 4 | TRUE | TRUE | TRUE |
| C2 | 4 | TRUE | TRUE | TRUE |
| HSP 27 | 4 | FALSE | TRUE | TRUE |
| IL16 | 4 | TRUE | FALSE | TRUE |
| EN-RAGE | 4 | FALSE | TRUE | TRUE |
| LOX-1 | 4 | FALSE | TRUE | FALSE |
| CEACAM8 | 4 | FALSE | TRUE | FALSE |
